# Penetrance and Pleiotropy of Polygenic Risk Scores for Schizophrenia, Bipolar Disorder, and Depression Among Adults in the US Veterans Affairs Health Care System

**DOI:** 10.1001/jamapsychiatry.2022.2742

**Published:** 2022-09-14

**Authors:** Tim B. Bigdeli, Georgios Voloudakis, Peter B. Barr, Bryan R. Gorman, Giulio Genovese, Roseann E. Peterson, David E. Burstein, Vlad I. Velicu, Yuli Li, Rishab Gupta, Manuel Mattheisen, Simone Tomasi, Nallakkandi Rajeevan, Frederick Sayward, Krishnan Radhakrishnan, Sundar Natarajan, Anil K. Malhotra, Yunling Shi, Hongyu Zhao, Thomas R. Kosten, John Concato, Timothy J. O’Leary, Ronald Przygodzki, Theresa Gleason, Saiju Pyarajan, Mary Brophy, Grant D. Huang, Sumitra Muralidhar, J. Michael Gaziano, Mihaela Aslan, Ayman H. Fanous, Philip D. Harvey, Panos Roussos

**Affiliations:** 1 VA New York Harbor Healthcare System, Brooklyn; 2 Institute for Genomics in Health, SUNY Downstate Health Sciences University, Brooklyn, New York; 3 Department of Psychiatry and Behavioral Sciences, SUNY Downstate Health Sciences University, Brooklyn, New York; 4 James J. Peters Veterans Affairs Medical Center, Bronx, New York; 5 Departments of Genetics and Genomic Sciences, Icahn School of Medicine at Mount Sinai, New York; 6 Psychiatry, Icahn School of Medicine at Mount Sinai, New York, New York; 7 Massachusetts Area Veterans Epidemiology, Research, and Information Center (MAVERIC), Jamaica Plain; 8 Stanley Center for Psychiatric Research, Broad Institute of MIT and Harvard, Cambridge, Massachusetts; 9 Harvard Medical School, Boston, Massachusetts; 10 Department of Psychiatry, Virginia Commonwealth University, Richmond; 11 Clinical Epidemiology Research Center (CERC), VA Connecticut Healthcare System, West Haven, Connecticut; 12 Yale University School of Medicine, New Haven, Connecticut; 13 Department of Psychiatry, Brigham & Women’s Hospital, Harvard Medical School, Boston, Massachusetts; 14 Department of Psychiatry, Dalhousie University, Halifax, Nova Scotia, Canada; 15 Department of Community Health, Dalhousie University, Halifax, Nova Scotia, Canada; 16 Department of Epidemiology, Dalhousie University, Halifax, Nova Scotia, Canada; 17 National Mental Health and Substance Use Policy Laboratory, Substance Abuse and Mental Health Services Administration, Rockville, Maryland; 18 Center for Psychiatric Neuroscience, Feinstein Institute for Medical Research, Manhasset, New York; 19 Division of Psychiatry Research, The Zucker Hillside Hospital, Northwell Health, Glen Oaks, New York; 20 Department of Psychiatry, Hofstra Northwell School of Medicine, Hempstead, New York; 21 Michael E. DeBakey VA Medical Center, Houston, Texas; 22 Department of Psychiatry, Neuroscience, Pharmacology, and Immunology and Rheumatology, Baylor College of Medicine, Houston, Texas; 23 Center for Drug Evaluation and Research, Food and Drug Administration, Silver Spring, Maryland; 24 Office of Research and Development, Veterans Health Administration, Washington, DC; 25 Boston University School of Medicine, Boston, Massachusetts; 26 Department of Psychiatry, University of Arizona College of Medicine-Phoenix, Phoenix; 27 Carl T. Hayden Veterans Affairs Medical Center, Phoenix, Arizona; 28 Bruce W. Carter Miami Veterans Affairs (VA) Medical Center, Miami, Florida; 29 University of Miami Miller School of Medicine, Miami, Florida

## Abstract

**Importance:**

Serious mental illnesses, including schizophrenia, bipolar disorder, and depression, are heritable, highly multifactorial disorders and major causes of disability worldwide.

**Objective:**

To benchmark the penetrance of current neuropsychiatric polygenic risk scores (PRSs) in the Veterans Health Administration health care system and to explore associations between PRS and broad categories of human disease via phenome-wide association studies.

**Design, Setting, and Participants:**

Extensive Veterans Health Administration’s electronic health records were assessed from October 1999 to January 2021, and an embedded cohort of 9378 individuals with confirmed diagnoses of schizophrenia or bipolar 1 disorder were found. The performance of schizophrenia, bipolar disorder, and major depression PRSs were compared in participants of African or European ancestry in the Million Veteran Program (approximately 400 000 individuals), and associations between PRSs and 1650 disease categories based on *ICD-9/10* billing codes were explored. Last, genomic structural equation modeling was applied to derive novel PRSs indexing common and disorder-specific genetic factors. Analysis took place from January 2021 to January 2022.

**Main Outcomes and Measures:**

Diagnoses based on in-person structured clinical interviews were compared with *ICD-9/10* billing codes. PRSs were constructed using summary statistics from genome-wide association studies of schizophrenia, bipolar disorder, and major depression.

**Results:**

Of 707 299 enrolled study participants, 459 667 were genotyped at the time of writing; 84 806 were of broadly African ancestry (mean [SD] age, 58 [12.1] years) and 314 909 were of broadly European ancestry (mean [SD] age, 66.4 [13.5] years). Among 9378 individuals with confirmed diagnoses of schizophrenia or bipolar 1 disorder, 8962 (95.6%) were correctly identified using *ICD-9/10* codes (2 or more). Among those of European ancestry, PRSs were robustly associated with having received a diagnosis of schizophrenia (odds ratio [OR], 1.81 [95% CI, 1.76-1.87]; *P* < 10^−257^) or bipolar disorder (OR, 1.42 [95% CI, 1.39-1.44]; *P* < 10^−295^). Corresponding effect sizes in participants of African ancestry were considerably smaller for schizophrenia (OR, 1.35 [95% CI, 1.29-1.42]; *P* < 10^−38^) and bipolar disorder (OR, 1.16 [95% CI, 1.11-1.12]; *P* < 10^−10^). Neuropsychiatric PRSs were associated with increased risk for a range of psychiatric and physical health problems.

**Conclusions and Relevance:**

Using diagnoses confirmed by in-person structured clinical interviews and current neuropsychiatric PRSs, the validity of an electronic health records–based phenotyping approach in US veterans was demonstrated, highlighting the potential of PRSs for disentangling biological and mediated pleiotropy.

## Key Points

**Question:**

What is the penetrance of polygenic risk scores (PRSs) for schizophrenia, bipolar disorder, and major depression among US veterans who use the Veterans Health Administration health care system and what health problems are associated with having a higher polygenic burden?

**Findings:**

In this cross-sectional study of 707 299 individuals, PRSs were associated with having ever received a relevant psychiatric diagnosis and were enriched among more frequently hospitalized patients. Higher PRSs were associated with increased odds for numerous mental and physical health diagnoses, even among individuals who lack a formal diagnosis.

**Meaning:**

Individual-level PRSs informed by large-scale genetic studies are portable across US health care systems and have emergent potential for risk stratification, albeit with disparate specificity across ancestries.

## Teaser

This cross-sectional study benchmarks the penetrance of current neuropsychiatric polygenic risk scores in the Veterans Health Administration health care system, and explores associations between polygenic risk scores and broad categories of human disease via phenome-wide association studies.

## Introduction

Serious mental illnesses such as schizophrenia, bipolar disorder, and major depression are leading causes of disability and public health expenditure, and affected persons disproportionately experience increased morbidity and early mortality. Recent years have seen important advances in our understanding of the complex multifactorial underpinnings of serious mental illnesses, with genome-wide association studies (GWAS) yielding robust and replicable associations with specific loci (270, 64, and 44 for schizophrenia, bipolar disorder, and major depression, respectively).^1,2,3^ However, small effect sizes at individual variants and extreme polygenicity have thwarted the transformative mechanistic insights needed for development of novel therapeutics and prevention strategies.

Polygenic risk scores (PRSs) aggregate genetic associations across the genome, including many variants that do not attain genome-wide significance and can account for more variance in liability than genome-wide significant findings alone, albeit they are typically less predictive than a positive family history^4^ or certain rare copy number variants.^5^ Ever-increasing GWAS sample sizes have seen the variance in liability captured by PRSs climb steadily, from 3% in the first demonstrative application to schizophrenia^6^ to upwards of 10% in recent Psychiatric Genomics Consortium analyses.^1^ As applied to nonpsychiatric traits, the clinical utility of PRSs is emergent,^7^ and potential applications in psychiatry are actively being explored,^8^ including risk stratification and predicting treatment response.

With large biobanks now linking the electronic health records (EHRs) of hundreds of thousands of patients to their individual-level genomic data, there are opportunities to explore the associations of PRSs (or specific variants) with a wide range of clinical phenotypes, ie, a genotype-to-phenotype or reverse genetics paradigm. Also known as phenome-wide association studies (PheWAS),^9^ this unbiased, disease-agnostic approach has the potential to uncover hitherto unrecognized associations between distinct diagnostic entities and to aid in disentangling complex pleiotropic associations. A recent application of PheWAS from the PsycheMERGE Consortium analyzed schizophrenia PRSs in more than 100 000 patients from 4 large health care systems (Geisinger Health System, Mount Sinai Health System, Partners HealthCare System, and Vanderbilt University Medical Center) and uncovered robust associations with both psychiatric and nonpsychiatric diagnoses.^10^

The Veterans Health Administration (VHA) is the largest integrated health care system in the United States, with 171 medical centers and 1112 outpatient clinics serving more than 9 million veterans. Launched in 2010, the Million Veteran Program (MVP) is a landmark endeavor that links genomic laboratory testing, survey-based self-report data, and EHRs spanning decades, with the goal of creating a mega-biobank and novel evidence base for precision medicine initiatives.^11^ Demographically and clinically, the 850 000 enrolled participants reflect the population that uses the VHA, with overrepresentation of older individuals and male individuals, as well as higher rates of multiple, chronic conditions compared with the general population,^12,13^ despite better access to health care.

Cooperative Studies Program (CSP) #572^14^ is a cohort of approximately 9300 veterans with schizophrenia or bipolar 1 disorder who received detailed in-person assessments of clinical diagnosis, functioning, and symptomatology. Within this companion study to the MVP, we evaluated the sensitivity of *ICD-9*/*10* billing codes for schizophrenia, bipolar disorder, and major depression, applying case-control definitions of varying stringency and breadth of clinical phenotype (eg, schizophrenia vs any psychosis). We benchmarked the penetrance of current neuropsychiatric PRSs for schizophrenia,^1^ bipolar disorder,^2^ and major depression^3^ in 400 000 ancestrally diverse MVP participants and explored the broad associations of PRS with physical and mental health conditions via PheWAS. Finally, recognizing the considerable shared genetic basis of these disorders, we applied genomic structural equation modeling to derive common and disorder-specific latent genetic factors for comparative genomic analyses and explored pleiotropic associations of these latent factors with PheWAS.

## Methods

### Study Participants

This study was approved by the Veterans Affairs (VA) Central Institutional Review Board, and all patients provided written informed consent. Additional details of study ascertainment and assessment are described elsewhere.^14^

#### CSP #572

Participants were recruited through their clinicians, posted notices at participating VA hospitals, and through word of mouth from January 2011 to January 2020. All patients received the Structured Clinical Interview for the *DSM*^15^ and met lifetime *DSM-IV* criteria for schizophrenia (n = 3953) or bipolar 1 disorder (n = 5425). Patients with major neurologic illnesses or medical problems that could interfere with central nervous system function were excluded. Information from medical records, patients’ clinicians, or other informants were used, if needed, to confirm diagnoses. Diagnosed substance misuse was not an exclusion criterion, given some concerns about representativeness. Participants received a brief cognitive assessment and the University of California, San Diego, Performance-Based Skills Assessment, Brief version, a performance-based measure of everyday functional skills.^16^

#### MVP

Participants were active users of the VHA health care system and were recruited through invitational mailings or by MVP staff while receiving clinical care. Informed consent and authorization per the Health Insurance Portability and Accountability Act were the only other inclusion criteria. Participants were recruited from January 2011 to January 2020. All participants completed a baseline survey, which includes information on demographic factors, health status, lifestyle habits, military experiences, medical history, and family history of specific illnesses and physical features; many also completed an optional lifestyle survey.^11^

At the time of manuscript submission, 4697 individuals (approximately 50% of CSP #572 and approximately 0.7% of MVP) were dually enrolled in both CSP #572 and MVP. CSP #572 and MVP participants were genotyped on the MVP 1.0 Axiom array^17^ (eMethods in Supplement 1). Participants were classified as being of African or European ancestry using the harmonized ancestry and race and ethnicity method,^18^ which combines information on genetic ancestry with self-identified race and ethnicity.

### EHRs

For 9378 CSP #572 participants and 697 921 nonoverlapping MVP enrollees, we extracted *ICD-9/10* billing codes related to schizophrenia, bipolar disorder, and major depression (eTable 1 in Supplement 1) and prescription records for commonly prescribed antipsychotics, mood stabilizers, and antidepressants (eTable 2 in Supplement 1) from the VHA Corporate Data Warehouse.

We compared CSP #572 participants’ Structured Clinical Interview for the *DSM*–confirmed diagnoses with the *ICD-9*/*10* codes recorded in their EHRs. Given the challenge of the differential diagnosis, for individuals with both schizophrenia and bipolar disorder codes, we took the mode of the 5 most recent entries as the prevailing diagnosis.

### PRS Profiling

We constructed PRSs from published Psychiatric Genomics Consortium GWAS results,^1,2,3^ testing these for association with disease outcomes in the MVP cohort. Variants that met quality control filtering in both the training and target data sets were clumped in the appropriate 1000 Genomes Project phase 3 population (*r*^2^ > 0.1; 500-kb window), excluding the major histocompatibility complex. For varying *P *value thresholds in each training data set (eMethods in Supplement 1), scores were constructed by summing the number of tested alleles weighted by their effect estimates (ie, the log of the allelic odds ratio). To better facilitate comparison of our results with those based on civilian cohorts, we used a similar approach to Zheutlin et al,^10^ including comparing results based on a recently developed bayesian framework that applies continuous shrinkage to test statistics, PRS–continuous shrinkage.^19^

### Genomic Structural Equation Modeling

We used genomic structural equation modeling^20^ to model the genetic covariance structure underlying schizophrenia, bipolar disorder, and major depression. Briefly, genomic structural equation modeling models the multivariate genetic architecture of complex traits by estimating individual single-nucleotide variants (SNVs; formerly, single-nucleotide polymorphisms or SNPs) associations on latent constructs, is robust to sample overlap and sample-size imbalance, and does not require individual-level genotypes. We estimated the SNV associations with a common factor (shared across disorders) as well as associations specific to each disorder (eMethods in Supplement 1).

### PheWAS

We used PheWAS to explore the associations between neuropsychiatric PRSs and phecodes representing groupings of associated *ICD-9/10* billing codes.^9^ When testing individual phecodes, we required individuals with these disorders and controls to have 2 or more and zero codes, respectively. We applied logistic regression to test scaled PRSs (mean [SD], 0 [1]) for association with phecodes within ancestry groups, covarying for age, age^2^, sex, and 6 ancestry principal components.

We performed a series of sensitivity analyses, covarying for selected diagnoses or treatment with antipsychotics, mood stabilizers, and antidepressants, or removing individuals with any lifetime diagnosis of psychotic, mood, or substance disorders (eMethods in Supplement 1). Two-sided *P* values were statistically significant at 10^−5^. Analysis took place from January 2021 to January 2022.

## Results

### Participants

Of 707 299 enrolled study participants, 459 667 were genotyped at the time of writing; 84 806 were of broadly African ancestry (mean [SD] age, 58 [12.1] years), and 314 909 were of broadly European ancestry (mean [SD] age, 66.4 [13.5] years). Of 9378 individuals in CSP #572, 3953 (42%) had schizophrenia (median [SD] age, 56 [10.1] years; 289 [7.3%] female) and 5425 (58%) had bipolar 1 disorder (median [SD] age, 53 [11.5] years; 1005 [18.5%] female). There were 697 921 individuals in MVP (median [SD] age, 61 [14.2] years; 62 749 [9.0%] female). The Table and eTable 3 in Supplement 1 include descriptives for CSP #572 and MVP and display the number of participants meeting various case inclusion criteria based on phecodes and medications.

**Table.** Cooperative Studies Program (CSP) #572 and Million Veteran Program (MVP) Participants Meeting Varying Electronic Health Records–Based Criteria for Schizophrenia, Bipolar Disorder, and Major Depression

Diagnosis	Phecode	CSP #572						MVP^a^		
		Schizophrenia			Bipolar 1 disorder			≥1 *ICD-9/10*	≥2 *ICD-9/10*	Inpatient
		≥1 *ICD-9/10*	≥2 *ICD-9/10*	Inpatient	≥1 *ICD-9/10*	≥2 *ICD-9/10*	Inpatient			
Schizophrenia	295.1	3803 (96.2)	3770 (95.4)	2978 (75.3)	1821 (33.6)	1368 (25.2)	848 (15.6)	24 698 (3.5)	18 023 (2.6)	11 532 (1.7)
Paranoid	295.2	+8 (0.2)	+11 (0.3)	+9 (0.2)	+132 (2.4)	+99 (1.8)	+60 (1.1)	+4787 (0.69)	+2806 (0.4)	+1388 (0.2)
Psychosis	295.3	+26 (0.66)	+39 (1.0)	+64 (1.6)	+524 (9.7)	+402 (7.4)	+208 (3.8)	+19 905 (2.9)	+11 200 (1.6)	+5402 (0.8)
Total	NA	3837 (97.1)	3820 (96.6)	3051 (77.2)	2477 (45.7)	1869 (34.5)	1116 (20.6)	49 390 (7.1)	32 029 (4.6)	18 322 (2.6)
Bipolar disorder (mania)	296.1	610 (15.4)	462 (11.7)	189 (4.8)	3381 (62.3)	3056 (56.3)	1134 (20.9)	16 819 (2.4)	10 432 (1.5)	3919 (0.6)
Bipolar disorder (any)	296.1	+1063 (26.9)	691 (17.5)	+613 (15.5)	+1858 (34.2)	+2136 (39.4)	+2660 (49.0)	+62 394 (8.9)	+43 343 (6.2)	+20 228 (2.9)
Total	NA	1673 (42.3)	1153 (29.2)	802 (20.3)	5239 (96.6)	5192 (95.7)	3794 (69.9)	79 213 (11.3)	53 775 (7.7)	24 147 (3.5)
Depression	296.2	2997 (75.8)	2506 (63.4)	1529 (38.7)	4490 (82.8)	4048 (74.6)	2139 (39.4)	332 104 (47.6)	288 561 (41.3)	120 713 (17.3)
**Treatment**		**≥1 Rx**	**≥2 Trials**		**≥1 Rx**	**≥2 Trials**		**≥1 Rx**	**≥2 Trials**	
Antipsychotics		3784 (95.7)	3413 (86.3)		4793 (88.4)	3816 (70.3)		121 339 (17.4)	56 276 (8.1)	
Mood stabilizers		1834 (46.4)	706 (17.9)		4854 (89.5)	3319 (61.2)		102 498 (14.7)	33 340 (4.8)	
Antidepressants		2928 (74.1)	1921 (48.6)		4529 (83.5)	3446 (63.5)		357 795 (51.3)	218 864 (31.4)	

Abbreviations: NA, not applicable; Rx, prescription.

^a^ Participants dually enrolled in CSP #572 and MVP were excluded.

### Validation of EHR-Derived Phenotypes

We first sought to evaluate the precision and accuracy of EHR-derived phenotypes to capture caseness based on the Structured Clinical Interview for the *DSM*–based diagnoses available in CSP #572. Among 9378 patients, 8962 (95.6%) were correctly assigned using 2 or more relevant phecodes.

Overall, 1153 of 3953 confirmed patients with schizophrenia (29.2%) had 2 or more bipolar disorder–related phecodes, and 1869 of 5425 confirmed patients with bipolar 1 disorder (34.5%) had multiple codes for schizophrenia. Taking the prevailing diagnosis resulted in 111 of 3953 individuals with schizophrenia (2.8%) and 507 of 5425 (9.3%) with bipolar 1 disorder being misclassified. For most individuals with misclassified bipolar 1 disorder, the prevailing diagnosis was schizoaffective disorder; these patients were more often of African ancestry (odds ratio [OR], 2.24 [95% CI, 1.83-2.74]; *P* < 10^−14^), male (OR, 1.60 [95% CI, 1.22-2.11]; *P* < .01), and had lower University of California, San Diego, Performance-Based Skills Assessment, Brief version scores (β = –0.09 [95% CI, –0.13 to –0.06]; *P* < 10^−6^) (eTables 4-5 in Supplement 1).

**Figure 1.**
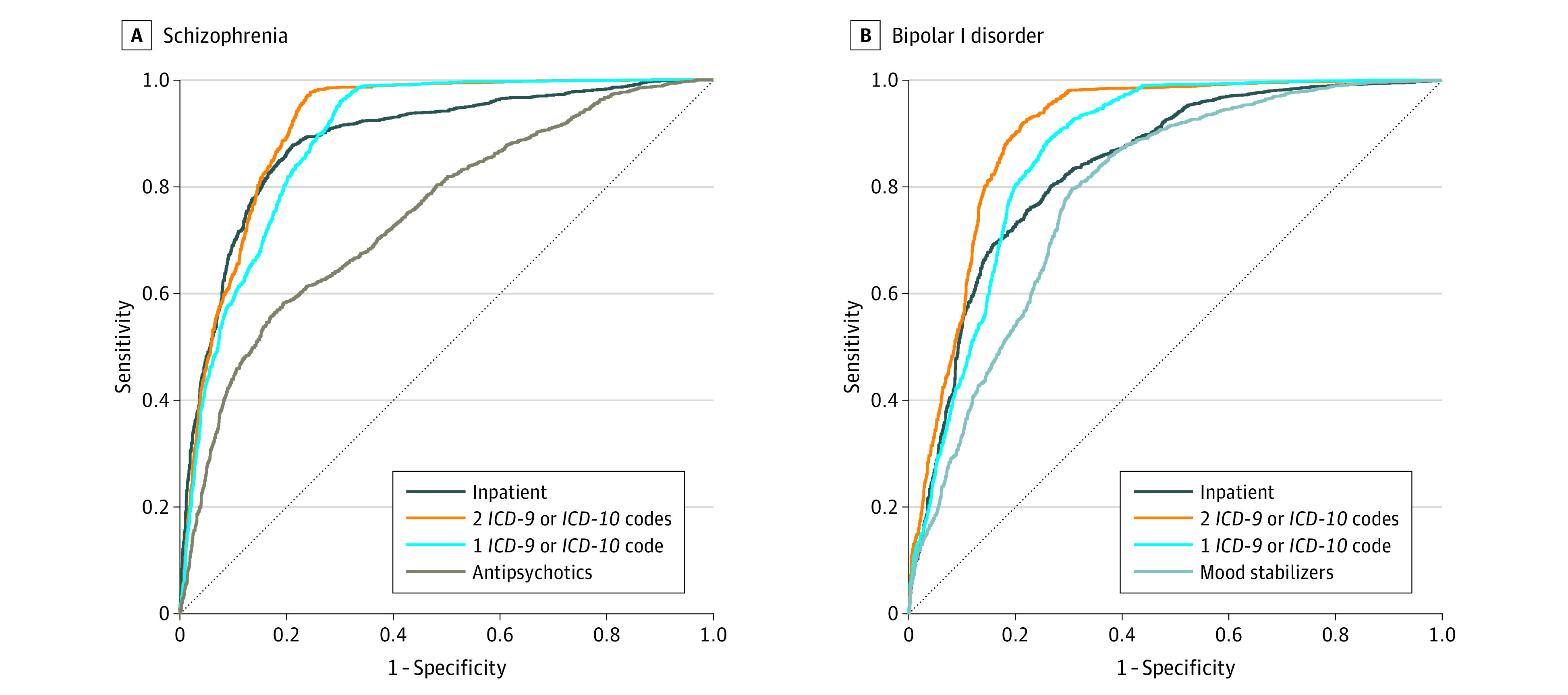
Prediction of Structured Clinical Interview for the *DSM–*Confirmed Diagnoses From Electronic Health Record–Based Criteria For varying schizophrenia (A) and bipolar 1 disorder (B) case criteria displayed in the Table, sensitivity and specificity estimates for a split-half cross-validation experiment are displayed. In each panel, the dashed line indicates a random (50/50) prediction*.*

Comparing receiver operating characteristic curves for predictive models based on the varying criteria displayed in the Table, we concluded that a minimum of 2 phecodes offered the best overall balance of sensitivity vs specificity (Figure 1; eTable 6 in Supplement 1).

### Penetrance of Neuropsychiatric PRS in the VA Health Care System

**Figure 2.**
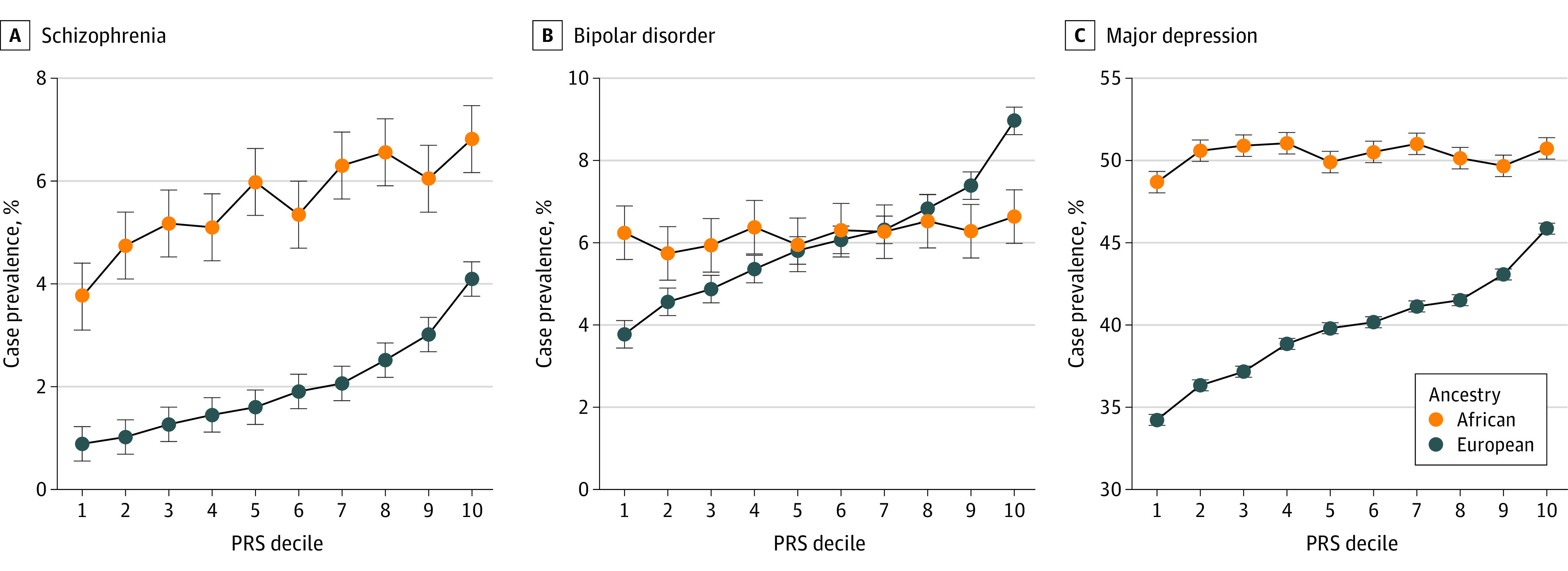
Case Prevalence by Neuropsychiatric Polygenic Risk Score (PRS) Decile in the Million Veteran Program For PRSs constructed from current Psychiatric Genomics Consortium summary statistics for schizophrenia, bipolar disorder, and major depression, the estimated prevalence of that diagnosis in each PRS decile is displayed separately for populations of African and European ancestries. Ascertained patients enrolled in Cooperative Studies Program #572 were excluded.

Benchmarking results for neuropsychiatric PRSs based on varying *P *value thresholds are given in eTables 7 to 9 in Supplement 1. Case prevalence estimates for each decile of PRS, representing the absolute disease prevalence, are displayed in Figure 2. As expected, the prevalence of serious mental illnesses was higher among veterans treated at VHA facilities than in the general population.^21,22^

The prevalence of schizophrenia among participants of European ancestry in the top and bottom deciles of schizophrenia PRS was 4% and 0.9%, respectively, corresponding to 4.8-fold higher odds (95% CI, 4.22-5.43; *P* < 10^−131^). Individuals in the top decile had approximately 2.4-fold higher odds of being diagnosed with schizophrenia than those below the 90% percentile (95% CI, 2.26-2.55; *P* < 10^−183^); 2-fold higher odds for psychosis (phecode 295.3) (95% CI, 1.85-2.09; *P* < 10^−101^); 1.6-fold higher odds for bipolar disorder (95% CI, 1.53-1.66; *P* < 10^−106^); and 1.2-fold (95% CI, 1.15-1.21; *P* < 10^−35^) for major depression.

Prevalence of bipolar disorder in the top and bottom bipolar disorder PRS deciles were 8.7% and 3.7%, equivalent to a 2.5-fold (95% CI, 2.34-2.71; *P* < 10^−135^) increase in risk. Compared with the bottom 90%, individuals in the top 10% had 1.7 times (95% CI, 1.61-1.75; *P* < 10^−126^) higher likelihood to be diagnosed with bipolar disorder.

Prevalence of major depression were 45% in the top PRS decile and 34% in the bottom decile, corresponding to 1.7-fold difference in risk (95% CI, 1.55-1.94; *P* < 10^−21^). Comparing the top 10% with the individuals in the remaining 90%, we observed an approximately 1.4-fold increase in risk (95% CI, 1.26-1.45; *P* < 10^−16^).

### Cross-Ancestry Portability of Neuropsychiatric PRS

The higher prevalence of schizophrenia and major depression among individuals of African ancestry was largely not associated with individuals’ risk strata (Figure 2) and as high as 6.8% and 51%, respectively, in the top 10%. In contrast, across bipolar disorder PRS deciles, the absolute prevalence of bipolar disorder was between 5.8% and 6.8%.

Individuals of African ancestry in the top decile of schizophrenia PRSs had approximately 1.4-fold higher risk of diagnoses of schizophrenia than those below the 90% percentile (95% CI, 1.34-1.57; *P* < 10^−18^) and 2.2-fold higher risk than those in bottom decile (95% CI, 1.89-2.65; *P* < 10^−19^). At extremes, odds of bipolar disorder and major depression were increased 1.6-fold (95% CI, 1.33-1.84; *P* < 10^−7^) and 1.3-fold (95% CI, 1.17-1.39; *P* < 10^−7^), respectively.

### Schizophrenia PRS Have Equivalent Relative Penetrance in Civilian and Veteran Health Care Systems

The demographics of the US veteran population differ from cohorts recruited from civilian health care systems. For instance, the VA population is composed of mostly male individuals (approximately 90%) and has higher prevalence of neuropsychiatric illnesses.^23^ Using the same training GWAS and bayesian PRS^19^ approach as used by the PsycheMERGE consortium,^10^ we see a robust association of schizophrenia PRSs with schizophrenia diagnosis (OR per SD-unit increase, 1.56 [95% CI, 1.52-1.61]; *P* = 2.7 × 10^−222^) in the European ancestry subset of our cohort, which is within the confidence interval estimated in the PsycheMERGE study (OR per SD-unit increase, 1.55 [95% CI, 1.39-1.72]).^10^ That is, despite stark differences in absolute prevalence between these cohorts, estimates of relative risks did not differ substantively.

### Higher Loadings of Neuropsychiatric PRS in More Chronic Illness

We observed a trend of increased polygenic loading in more chronic illness presentations.^1,24^ Patients who received inpatient treatment for schizophrenia had significantly higher PRS than those who did not (OR per SD-unit increase, 1.25 [95% CI, 1.18-1.34]; *P* < 10^−11^); among these individuals, schizophrenia PRS was positively associated with number of hospitalizations (β = 0.198 [95% CI, 0.11-0.28]; *P* < 10^−5^), including after adjusting for individuals’ total number of comorbidities (eMethods and eTables 10-13 in Supplement 1).

We observed similar patterns of results for bipolar disorder PRSs and inpatient treatment (OR per SD-unit increase, 1.14 [95% CI, 1.11-1.19]; *P* < 10^−17^) and number of hospitalizations (β = 0.18 [95% CI, 0.11-0.26]; *P* < 10^−5^) (eTables 14-15 in Supplement 1) and between major depression PRS and inpatient treatment (OR per SD-unit increase, 1.04 [95% CI, 1.03-1.06]; *P* < 10^−8^) and number of hospitalizations (β = 0.04 [95% CI, 0.01-0.07]; *P* = .00764) (eTables 16-17 in Supplement 1).

Associations between schizophrenia and bipolar disorder PRSs with inpatient treatment and number of hospitalizations were replicable in participants of African ancestry (eTable 14-15 in Supplement 1).

### Pleiotropic Influences of Neuropsychiatric PRS

Higher polygenic loading for schizophrenia, bipolar disorder, and major depression was associated with increased odds for numerous psychiatric diagnoses and physical health conditions (eTables 18-20 and eFigures 1-3 in Supplement 1). Associations between schizophrenia and respiratory symptoms and infections and between major depression and cardiovascular disease, hypertension, diabetes, somatic symptoms, and respiratory problems were robust among veterans without a lifetime diagnosis of psychotic, mood, or substance use disorders or who received relevant pharmacological treatment. Comparing PheWAS results, we observed relative enrichments of schizophrenia polygenic risk in mental health problems and of major depression PRSs in circulatory, respiratory, and endocrine problems, among others (eFigures 4-7 in Supplement 1).

Associations between schizophrenia PRSs and dental problems, respiratory symptoms, skin infections, substance use disorders, and suicide were replicable among veterans of African ancestry (eTables 21-23 and eFigures 8-10 in Supplement 1) and remained significant after adjusting for lifetime diagnoses and medications.

**Figure 3.**
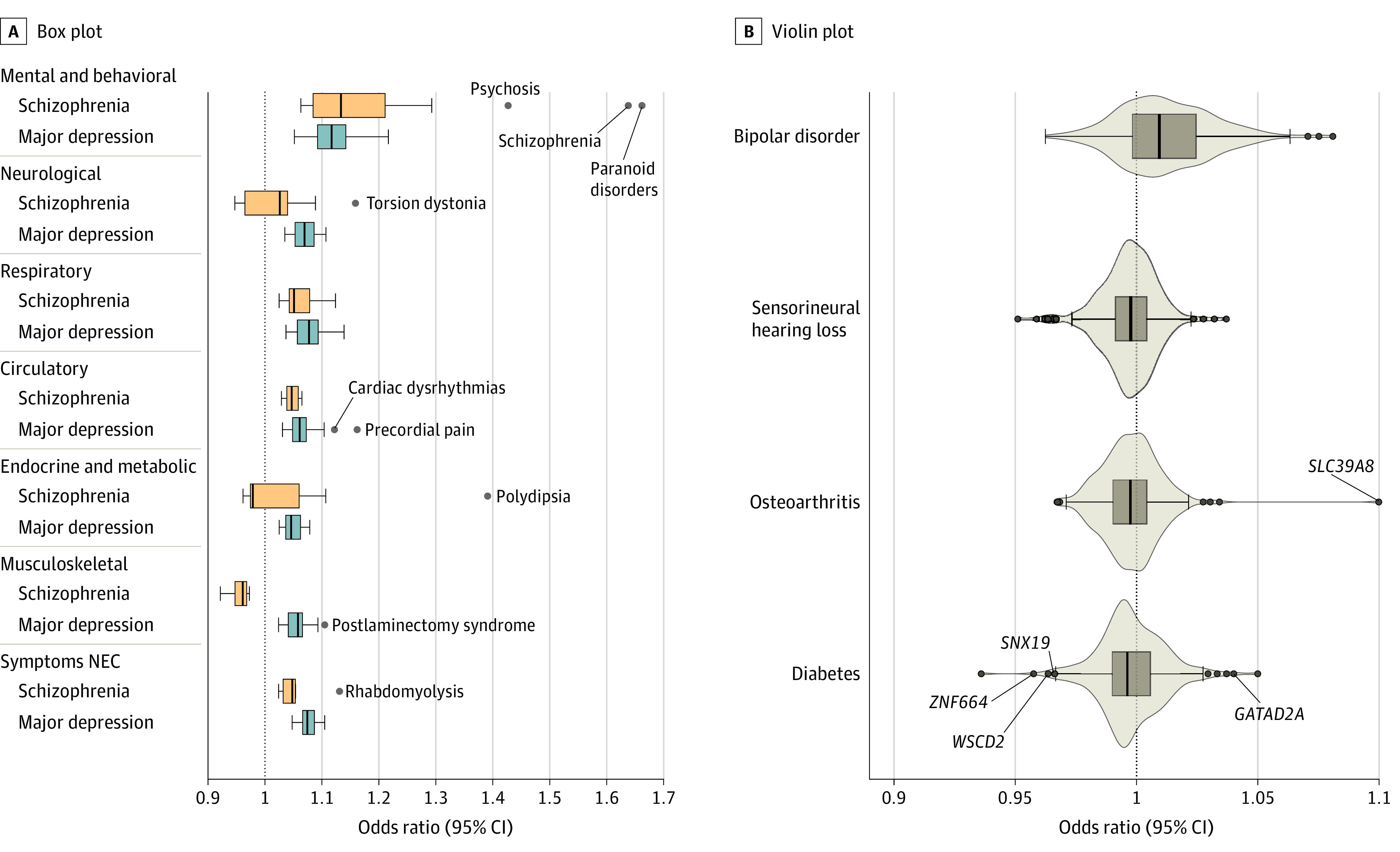
Phenome-Wide Association Studies Results for Neuropsychiatric Polygenic Risk Scores A, For significant results in phenome-wide association studies of schizophrenia and major depression polygenic risk scores, the distribution of effect sizes within each disease category is displayed as a boxplot. A null effect (ie, odds ratio, 1) is denoted as a dotted line. B, Effect sizes of Psychiatric Genomics Consortium^3^ schizophrenia loci on bipolar disorder (phecode 296.1), sensorineural hearing loss (phecode 389.1), osteoarthritis (phecode 740.1), and diabetes (phecode 250) in Million Veteran Program participants of European ancestry. A null effect is denoted as a dotted line. Labeled loci achieved genome-wide significance in tests of the target phenotype. NEC indicates not elsewhere classified.

We investigated the apparent protective effects of schizophrenia PRSs^10^ by examining associations between individual genome-wide significant schizophrenia loci^1^ and selected phecodes (Figure 3), based on the same linkage disequilibrium–independent SNVs and analytic framework as used for PheWAS. Using a simple binomial test, we found that significantly more schizophrenia-associated SNVs than expected by chance (ie, 50%) had reversed directions of associations with diabetes (168 of 270 [62.2%]; 95% CI, 0.56-0.68]; *P* = 7.07 × 10^−5^), hearing loss (165 of 270 [61.1%]; 95% CI, 0.55-0.67; *P* = .000313), and osteoarthritis (157 of 270 [58.1%]; 95% CI, 0.52-0.64; *P* = .00875). By comparison, we observed more SNVs than expected with convergent associations with bipolar disorder (191 of 270 [70.7%]; 95% CI, 0.65-0.76; *P* < 10^−11^).

### Genomic Structural Equation Modeling and Latent Factor PRS

Comparing our primary results with those based on latent genomic factors, we found that both schizophrenia-specific and common factor PRSs were associated with increased odds of psychosis-spectrum diagnoses (eTables 24-30 in Supplement 1). Observed protective associations of schizophrenia PRSs for sleep apnea, osteoarthritis, and hearing loss appear to be driven by schizophrenia-specific influences (eTables 24-25 in Supplement 1). The majority of associations between PRSs and broader psychiatric diagnoses and physical health problems were driven by a shared genetic liability (eTables 29-30 in Supplement 1).

### Polygenic Validation of the Psychosis-Affective Spectrum

**Figure 4.**
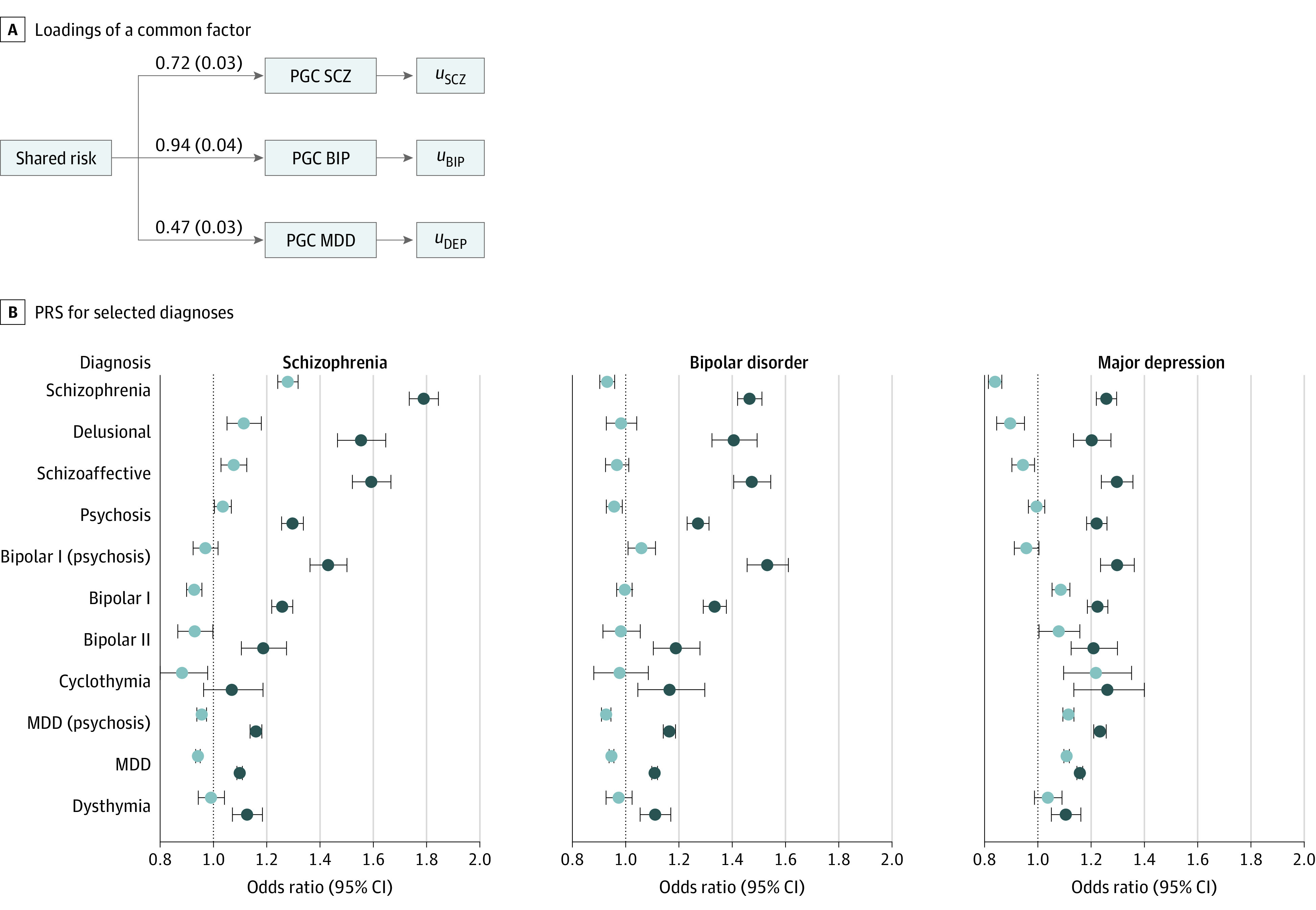
Association of Neuropsychiatric Polygenic Risk Scores (PRSs) With Psychotic and Affective Diagnoses A, Loadings of a common factor on schizophrenia (SCZ), bipolar disorder (BIP), and major depression (MDD) results and residual variances corresponding to disorder-specific effects. B, Odds ratios per SD unit increase in PRS for selected diagnoses compared against a common set of controls; analogous results based on latent, disorder-specific PRS appear are plotted in lighter hues. Case assignments were hierarchical and nonoverlapping. DEP indicates depression; PGC, Psychiatric Genomics Consortium.

We further explored the transdiagnostic spectrum concept via hierarchical assignments of participants to schizophrenia, bipolar disorder, major depression, or related diagnoses; schizoaffective disorders, bipolar II disorder, cyclothymia, and dysthymia were considered as intermediate categories of illness and were included in analyses given adequate sample sizes (eTables 31-32 in Supplement 1). Figure 4 displays estimated PRSs estimates across disorders, comparing individuals with these disorders to a common set of screened controls.

## Discussion

Building on our previous reports that published GWAS results are robustly generalizable to the US veteran population,^25^ we have demonstrated that the penetrance of schizophrenia PRSs is equivalent across VA and civilian health care systems, despite marked differences in absolute prevalence.^10^ Leveraging the VA’s extensive EHR, we confirm and extend reported associations between neuropsychiatric PRSs and broad disease categories in approximately 400 000 individuals. We derived novel, latent factors indexing disorder-specific and shared cross-disorder risk and attempted to disentangle widespread pleiotropy from confounding through extensive secondary modeling.

We first validated an EHR-based phenotyping approach in an embedded, well-characterized cohort with confirmed diagnoses of schizophrenia or bipolar 1 disorder and found that a simple approach^10^ requiring 2 or more *ICD-9/10* codes correctly identified approximately 95% of cases. Applying this phenotyping strategy to the full MVP cohort, we detected prevalence of schizophrenia, bipolar disorder, and major depression that are several-fold higher than in the general population, although representative of the US veteran population at large.^23^ Rates of schizophrenia and major depression among veterans of African ancestry were markedly elevated compared with veterans of European ancestry, which may be suggestive of implicit bias in diagnosis,^26^ self-selection bias for VHA utilization, limited alternatives for health care, or other structural issues.

Critically, despite markedly higher prevalence of schizophrenia, bipolar disorder, and major depression in the VA health care system, we did not find predictive values to be meaningfully attenuated. Schizophrenia PRSs yielded effect sizes for participants of European ancestry that were within the 95% CI of those reported by the PsychEMERGE consortium.^10^ The latter suggests that the penetrance of within-population relative PRSs in the US veteran population is equivalent to that of civilian cohorts despite nonrandom recruitment and exposure to distinct environmental factors and experiences.

Current neuropsychiatric PRSs were robustly associated with a range of psychiatric problems. Higher schizophrenia PRSs also increased risk of suicide, obsessive compulsive disorder and personality disorders, anxiety, and substance use behaviors, as well as a host of physical and somatic symptoms, recapitulating recent findings based on 4 US civilian health care systems.^10^ Notably, increased risks of certain infections and dental problems were detectable even when adjusting for psychotic and affective diagnoses and treatment, and excluding diagnosed substance use disorders, suggesting that neuropsychiatric liability may be penetrant even in individuals who lack a formal diagnosis. Other associations, such as those observed with erectile dysfunction and polydipsia, were explained by relevant diagnoses and adverse reactions of prescribed medications.^27^

We observed protective associations of schizophrenia PRSs with hearing loss, osteoarthritis, and diabetes, in contrast to widely documented, risk-increasing iatrogenic effects of second-generation antipsychotics.^28^ Post hoc analyses of schizophrenia-associated loci^1^ revealed an enrichment of SNVs at which the schizophrenia risk allele is protective for these conditions, suggestive of antagonistic pleiotropic effects.

Schizophrenia-specific PRSs were primarily associated with diagnoses of schizophrenia, paranoid disorders, psychosis, and schizoid personality disorder but only modestly with bipolar disorder, evincing some fidelity of published GWAS to Kraepelinian dichotomy. Intriguingly, major depression-specific and common factor PRSs, but not schizophrenia-specific PRSs, were strongly associated with increased number of comorbidities, suggesting that underlying confounding may be driving many of the observed pleiotropic associations. Future studies using mendelian randomization and within-family approaches may help to determine whether these findings are due to some likely causal influence or accounted for by other shared environmental risk factors (eg, socioeconomic status).

Strikingly, the higher prevalence of schizophrenia and major depression among veterans of African ancestry were largely not associated with individuals’ risk strata; for example, only veterans of European ancestry in the uppermost schizophrenia PRS decile had absolute risk comparable with those of African ancestry in the lowest decile. Despite lower cross-population generalizability of bipolar disorder and major depression PRSs, comparisons of individuals of African ancestry at extremes of PRSs yielded significant associations.

### Limitations

We did not attempt to model environmental or experiential differences associated with participants’ military service, which may partially explain increased rates of some illnesses. We did not specifically investigate the implications of predominantly male ascertainment in MVP. CSP #572 participants largely served in the period between the Vietnam War and Gulf War conflicts, while MVP participants’ service eras were more broadly distributed.

Because available EHR data are restricted to treatment received at VA facilities, any relevant medical history outside the VHA health system, including before or during participants’ military service, is limited to self-report.

We focused on populations of African and European ancestry in the current study because these broadly defined ancestries comprised the majority of the CSP #572 and MVP cohorts. Our ongoing work in this area will extend these findings to diverse Asian, Hispanic, and Latino populations.

## Conclusions

Application of current neuropsychiatric PRSs to the MVP yielded results consistent with multiple, continuous liability distributions underlying schizophrenia, bipolar disorder, and major depression,^29,30^ underscoring the advantages of multivariate and transdiagnostic approaches for studying these complex, heterogeneous clinical presentations.

## Notes

Supplement 1. eMethods. eTable 1. ICD-9/10 billing codes queried from VHA Corporate Data Warehouse eTable 2. Medications queried from VHA prescription records eTable 3. CSP #572 and MVP participants meeting varying EHR-based criteria (females only) eTable 4. Factors associated with diagnostic misclassification of BPI cases (as SCZ) in CSP #572 eTable 5. Factors associated with diagnostic misclassification of SCZ cases (as BPI) in CSP #572 eTable 6. Predictive models for SCZ or BPI diagnosis based on EHR eTable 7. Associations of neuropsychiatric PRSs with SCZ eTable 8. Associations of neuropsychiatric PRSs with BIP eTable 9. Associations of neuropsychiatric PRSs with DEP eTable 10. Associations of neuropsychiatric PRSs with total comorbidity burden among EA participants eTable 11. Associations of neuropsychiatric PRSs with total comorbidity burden among AA participants eTable 12. Associations of neuropsychiatric PRSs with inpatient treatment for SCZ eTable 13. Associations of neuropsychiatric PRSs with number of hospitalizations for SCZ eTable 14. Associations of neuropsychiatric PRSs with inpatient treatment for BIP eTable 15. Associations of neuropsychiatric PRSs with number of hospitalizations for BIP eTable 16. Associations of neuropsychiatric PRSs with inpatient treatment for DEP eTable 17. Associations of neuropsychiatric PRSs with number of hospitalizations for DEP eTable 18. Significant findings (p<10^-25^) in PheWAS of SCZ PRS in EA participants eTable 19. Significant findings (p<10^-25^) in PheWAS of BIP PRS in EA participants eTable 20. Significant findings (p<10^-25^) in PheWAS of DEP PRS in EA participants eTable 21. Significant findings (p<10^-5^) in PheWAS of SCZ PRS in AA participants eTable 22. Significant findings (p<10^-5^) in PheWAS of BIP PRS in AA participants eTable 23. Significant findings (p<10^-5^) in PheWAS of DEP PRS in AA participants eTable 24. Significant findings (p<10^-25^) in PheWAS of SCZ-specific PRS in EA participants eTable 25. Significant findings (p<10^-5^) in PheWAS of SCZ-specific PRS in AA participants eTable 26. Significant findings (p<10^-25^) in PheWAS of BIP-specific PRS in EA participants eTable 27. Significant findings (p<10^-25^) in PheWAS of DEP-specific PRS in EA participants eTable 28. Significant findings (p<10^-5^) in PheWAS of DEP-specific PRS in AA participants eTable 29. Significant findings (p<10^-25^) in PheWAS of common factor PRS in EA participants eTable 30. Significant findings (p<10^-5^) in PheWAS of common factor PRS in AA participants eTable 31. Associations of neuropsychiatric PRSs with hierarchical diagnoses in EA participants eTable 32. Associations of neuropsychiatric PRSs with hierarchical diagnoses in AA participants eFigure 1. Odds ratios for top decile of SCZ PRS in EA participants eFigure 2. Odds ratios for top deciles of BIP PRS in EA participants eFigure 3. Odds ratios for top decile of DEP PRS in EA participants eFigure 4. PheWAS results for neuropsychiatric PRS eFigure 5. Relative enrichments of SCZ versus BIP PheWAS results across disease categories eFigure 6. Relative enrichments of SCZ and DEP PheWAS results across disease categories eFigure 7. Relative enrichments of BIP and DEP PheWAS results across disease categories eFigure 8. Odds ratios for top deciles of SCZ PRS in AA participants eFigure 9. Odds ratios for top deciles of BIP PRS in AA participants eFigure 10. Odds ratios for top deciles of DEP PRS in AA participants

Supplement 2. **Nonauthor collaborators. **The Cooperative Studies Program (CSP) #572 and Million Veteran Program (MVP) investigators
