# Supplementary material for: Penetrance and Pleiotropy of Polygenic Risk Scores for Schizophrenia, Bipolar Disorder, and Depression Among Adults in the US Veterans Affairs Health Care System
Source: JAMA Psychiatry. 2022 Sep 14;79(11):1092–101. doi: 10.1001/jamapsychiatry.2022.2742 (PMC9475441; doi:10.1001/jamapsychiatry.2022.2742)
Supplement: Supplement 1. — eMethods. eTable 1. ICD-9/10 billing codes queried from VHA Corporate Data Warehouse eTable 2. Medications queried from VHA prescription records eTable 3. CSP #572 and MVP participants meeting varying EHR-based criteria (females only) eTable 4. Factors associated with diagnostic misclassification of BPI cases (as SCZ) in CSP #572 eTable 5. Factors associated with diagnostic misclassification of SCZ cases (as BPI) in CSP #572 eTable 6. Predictive models for SCZ or BPI diagnosis based on EHR eTable 7. Associations of neuropsychiatric PRSs with SCZ eTable 8. Associations of neuropsychiatric PRSs with BIP eTable 9. Associations of neuropsychiatric PRSs with DEP eTable 10. Associations of neuropsychiatric PRSs with total comorbidity burden among EA participants eTable 11. Associations of neuropsychiatric PRSs with total comorbidity burden among AA participants eTable 12. Associations of neuropsychiatric PRSs with inpatient treatment for SCZ eTable 13. Associations of neuropsychiatric PRSs with number of hospitalizations for SCZ eTable 14. Associations of neuropsychiatric PRSs with inpatient treatment for BIP eTable 15. Associations of neuropsychiatric PRSs with number of hospitalizations for BIP eTable 16. Associations of neuropsychiatric PRSs with inpatient treatment for DEP eTable 17. Associations of neuropsychiatric PRSs with number of hospitalizations for DEP eTable 18. Significant findings (p<10-25) in PheWAS of SCZ PRS in EA participants eTable 19. Significant findings (p<10-25) in PheWAS of BIP PRS in EA participants eTable 20. Significant findings (p<10-25) in PheWAS of DEP PRS in EA participants eTable 21. Significant findings (p<10-5) in PheWAS of SCZ PRS in AA participants eTable 22. Significant findings (p<10-5) in PheWAS of BIP PRS in AA participants eTable 23. Significant findings (p<10-5) in PheWAS of DEP PRS in AA participants eTable 24. Significant findings (p<10-25) in PheWAS of SCZ-specific PRS in EA participants eTable 25. Significant findings (p<10-5 [file jamapsychiatry-e222742-s001.pdf]

Supplemental Online Content

Bigdeli TB, Voloudakis G, Barr PB, et al; Cooperative Studies Program (CSP) #572 and Million Veteran Program (MVP). Penetrance and pleiotropy of polygenic risk scores for schizophrenia, bipolar disorder, and depression among adults in the US Veterans Affairs health care system. *JAMA Psychiatry*. Published online September 14, 2022. doi:10.1001/jamapsychiatry.2022.2742

eMethods.
eTable 1. ICD-9/10 billing codes queried from VHA Corporate Data Warehouse
eTable 2. Medications queried from VHA prescription records
eTable 3. CSP #572 and MVP participants meeting varying EHR-based criteria (females only**)**
eTable 4. Factors associated with diagnostic misclassification of BPI cases (as SCZ) in CSP #572
eTable 5. Factors associated with diagnostic misclassification of SCZ cases (as BPI) in CSP #572
eTable 6. Predictive models for SCZ or BPI diagnosis based on EHR
eTable 7. Associations of neuropsychiatric PRSs with SCZ
eTable 8. Associations of neuropsychiatric PRSs with BIP
eTable 9. Associations of neuropsychiatric PRSs with DEP
eTable 10. Associations of neuropsychiatric PRSs with total comorbidity burden among EA participants
eTable 11. Associations of neuropsychiatric PRSs with total comorbidity burden among AA participants
eTable 12. Associations of neuropsychiatric PRSs with inpatient treatment for SCZ
eTable 13. Associations of neuropsychiatric PRSs with number of hospitalizations for SCZ
eTable 14. Associations of neuropsychiatric PRSs with inpatient treatment for BIP
eTable 15. Associations of neuropsychiatric PRSs with number of hospitalizations for BIP
eTable 16. Associations of neuropsychiatric PRSs with inpatient treatment for DEP
eTable 17. Associations of neuropsychiatric PRSs with number of hospitalizations for DEP
eTable 18. Significant findings ($p<10^{-25}$) in PheWAS of SCZ PRS in EA participants
eTable 19. Significant findings ($p<10^{-25}$) in PheWAS of BIP PRS in EA participants
eTable 20. Significant findings ($p<10^{-25}$) in PheWAS of DEP PRS in EA participants
eTable 21. Significant findings ($p<10^{-5}$) in PheWAS of SCZ PRS in AA participants
eTable 22. Significant findings ($p<10^{-5}$) in PheWAS of BIP PRS in AA participants
eTable 23. Significant findings ($p<10^{-5}$) in PheWAS of DEP PRS in AA participants
eTable 24. Significant findings ($p<10^{-25}$) in PheWAS of SCZ-specific PRS in EA participants
eTable 25. Significant findings ($p<10^{-5}$) in PheWAS of SCZ-specific PRS in AA participants
eTable 26. Significant findings ($p<10^{-25}$) in PheWAS of BIP-specific PRS in EA participants
eTable 27. Significant findings ($p<10^{-25}$) in PheWAS of DEP-specific PRS in EA participants
eTable 28. Significant findings ($p<10^{-5}$) in PheWAS of DEP-specific PRS in AA participants
eTable 29. Significant findings ($p<10^{-25}$) in PheWAS of common factor PRS in EA participants
eTable 30. Significant findings ($p<10^{-5}$) in PheWAS of common factor PRS in AA participants
eTable 31. Associations of neuropsychiatric PRSs with hierarchical diagnoses in EA participants
eTable 32. Associations of neuropsychiatric PRSs with hierarchical diagnoses in AA participants
eFigure 1. Odds ratios for top decile of SCZ PRS in EA participants
eFigure 2. Odds ratios for top deciles of BIP PRS in EA participants
eFigure 3. Odds ratios for top decile of DEP PRS in EA participants
eFigure 4. PheWAS results for neuropsychiatric PRS

© 2022 Bigdeli TB et al. *JAMA Psychiatry.*

eFigure 5. Relative enrichments of SCZ versus BIP PheWAS results across disease categories
eFigure 6. Relative enrichments of SCZ and DEP PheWAS results across disease categories
eFigure 7. Relative enrichments of BIP and DEP PheWAS results across disease categories
eFigure 8. Odds ratios for top deciles of SCZ PRS in AA participants
eFigure 9. Odds ratios for top deciles of BIP PRS in AA participants
eFigure 10. Odds ratios for top deciles of DEP PRS in AA participants

This supplemental material has been provided by the authors to give readers additional information about their work.

© 2022 Bigdeli TB et al. *JAMA Psychiatry*.

eMethods

Quality Control

Samples were required to have call rate >98.5%, heterozygosity which deviates less than 3 SD from the mean, and concordance between imputed genetic sex matches self-reported gender. A “greedy” algorithm was used to remove individuals with kinship coefficient ≥0.0884. Variants with MAF >0.01, call rate >98%, Hardy-Weinberg equilibrium *p*-value $>10^{-10}$, and imputation R^2 >0.9 were retained for analysis.

Relatedness checking

A greedy algorithm was used for excluding related individuals: related samples and samples with cryptic relationship were removed with a kinship coefficient cut-off of ≥ 0.0884 estimated by the KING v.2.0 software (PMID: 20926424) as previously described (PMID: 31358989, PMID: 31676865). First samples with multiple relationships (≥1) were removed, then individuals were removed from relationship pairs by keeping the individuals with the lowest variant missingness rates to maximize the quality of the remaining samples.

Screened control participants

We selected psychiatric control participants by leveraging the EHR, after exclusion of individuals with any EHR-recorded history of psychotic or affective disorders or past treatment with antipsychotic, mood-stabilizing, or antidepressant medications, or who self-endorsed a personal or family history of any of these conditions on the MVP core survey. Psychiatrically screened controls were utilized for benchmarking PRSs and for ease of interpretation in the cross-diagnostic analyses presented in Figure 4. We did not utilize screened controls in PheWAS analyses, as comparison “controls” were defined on a per-phecode basis.

Benchmarking PRS performance

We compared PRSs based on varying p-value thresholds (0.5, 0.05, and $5x10^{-8}$) between cases and screened controls. For SCZ, we observed the best predictive performance for PRSs based on SNPs with p<0.05 (eTable 7); and for BIP and DEP, the best-performing PRSs were based on SNPs with p<0.5 (eTables 8 and 9). For consistency and simplicity of explanation, we opted to highlight gSEM-based PRSs based on SNPs with p<0.5.

© 2022 Bigdeli TB et al. *JAMA Psychiatry*.

Genomic SEM

Our analyses were limited to high quality, common SNPs (minor allele frequency >0.01, imputation information>0.6) available across all three of the input GWAS and the 1000G Phase 3 reference data, resulting in a final set of 5,929,464 SNPs

The factor loadings for the shared factor are presented in Figure 4. Because this model is just-identified, there is no way to determine overall fit. However each of the phenotypes shows strong-to-modest loading on this latent factor (SCZ = 0.72; BIP = 0.94; DEP = 0.47). Genomic SEM corrects for inflation in test statistics due to population stratification by default (multiplying input GWAS standard errors by the square root of their LDSC intercept), so the inflation of the test-statistics in QQ-plots is indicative of polygenic signals in the resulting multivariate GWAS.

Of the genome wide significant SNPs ($p<5x10^{-8}$), we identified 83 independent lead SNPS (LD $r^2 < .1$) associated with the shared latent factor, 36 associated with SCZ-specific variance, 29 associated with DEP-specific variance, and 11 associated with BIP-specific variance. Of the SNPs associated with the shared latent factor, only 10 SNPs showed evidence of significant heterogeneity, using a liberal threshold of $p < 6.02x10^{-4}$, and the vast majority of SNPs seem robustly associated with the shared latent factor.

Sensitivity analyses

We followed-up our primary PheWAS by investigating whether our results remained significant after excluding individuals with a SCZ-related (phecode 295.*) or affective (phecode 296.*) diagnosis, or after covarying for any lifetime diagnosis of either, or for lifetime treatment with antipsychotics, mood-stabilizers, or antidepressants. Briefly, covariate adjustment was performed by inclusion of a “dummy” variable (0/1) indicating whether an individual had ever received a relevant diagnosis (eTable 1), or had any prescription record for any of the compiled medications listed in eTable 2.

We also created a proxy for comorbidity burden by tabulating the number of unique phecode terms for which each individual met our basic case/control criteria (i.e. 2 or more codes for cases, and zero for controls). We restricted this list to 600 “top codes”, excluding child terms or “leaf codes”. For example, we counted any two entries for phecode 295 (“schizophrenia and related conditions”) as meeting case criteria for a single

© 2022 Bigdeli TB et al. *JAMA Psychiatry*.

condition, but not separately consider phecodes 295.1 (“Schizophrenia”) and 295.3 (“Psychosis”); similarly, an individual could be classified as a case for phecode 296 (“mood disorders”) but not also 296.1 (“Bipolar”) and 296.2 (“Depression”). Our rationale here was that this approach would limit over-representation of ontologically related terms. We tested for main effects of PRSs on this comorbidity index using negative binomial regression, adjusting for the same covariates used in the primary PheWAS.

In case-only analyses examining associations between PRSs and number of hospitalizations, we additionally considered secondary models including binary covariates for having ever received a diagnosis of substance addiction and disorders (phecode 316) or alcoholism (phecode 317).

© 2022 Bigdeli TB et al. *JAMA Psychiatry*.

eTable 1. ICD-9/10 billing codes queried from VHA Corporate Data Warehouse.

ICD-9/10 codes for SCZ, BIP, DEP, and other psychotic and mood diagnoses, with corresponding phecodes.

Phenotype	Phecode	Type	Code	Description	Note
SCZ	295.1	ICD-9	295	Simple type schizophrenia	
SCZ	295.1	ICD-9	295	Simple type schizophrenia, unspecified state	
SCZ	295.1	ICD-9	295.01	Simple type schizophrenia, subchronic state	
SCZ	295.1	ICD-9	295.02	Simple type schizophrenia, chronic state	
SCZ	295.1	ICD-9	295.03	Simple type schizophrenia, subchronic state with acute exacerbation	
SCZ	295.1	ICD-9	295.04	Simple type schizophrenia, chronic state with acute exacerbation	
SCZ	295.1	ICD-9	295.05	Simple type schizophrenia, in remission	
SCZ	295.1	ICD-9	295.1	Disorganized type schizophrenia	
SCZ	295.1	ICD-9	295.1	Disorganized type schizophrenia, unspecified state	
SCZ	295.1	ICD-9	295.11	Disorganized type schizophrenia, subchronic state	
SCZ	295.1	ICD-9	295.12	Disorganized type schizophrenia, chronic state	
SCZ	295.1	ICD-9	295.13	Disorganized type schizophrenia, subchronic state with acute exacerbation	
SCZ	295.1	ICD-9	295.14	Disorganized type schizophrenia, chronic state with acute exacerbation	
SCZ	295.1	ICD-9	295.15	Disorganized type schizophrenia, in remission	
SCZ	295.1	ICD-9	295.2	Catatonic type schizophrenia	
SCZ	295.1	ICD-9	295.2	Catatonic type schizophrenia, unspecified state	
SCZ	295.1	ICD-9	295.21	Catatonic type schizophrenia, subchronic state	
SCZ	295.1	ICD-9	295.22	Catatonic type schizophrenia, chronic state	
SCZ	295.1	ICD-9	295.23	Catatonic type schizophrenia, subchronic state with acute exacerbation	
SCZ	295.1	ICD-9	295.24	Catatonic type schizophrenia, chronic state with acute exacerbation	
SCZ	295.1	ICD-9	295.25	Catatonic type schizophrenia, in remission	
SCZ	295.1	ICD-9	295.3	Paranoid type schizophrenia	
SCZ	295.1	ICD-9	295.3	Paranoid type schizophrenia, unspecified state	
SCZ	295.1	ICD-9	295.31	Paranoid type schizophrenia, subchronic state	
SCZ	295.1	ICD-9	295.32	Paranoid type schizophrenia, chronic state	
SCZ	295.1	ICD-9	295.33	Paranoid type schizophrenia, subchronic state with acute exacerbation	
SCZ	295.1	ICD-9	295.34	Paranoid type schizophrenia, chronic state with acute exacerbation	
SCZ	295.1	ICD-9	295.35	Paranoid type schizophrenia, in remission	
SCZ	295.1	ICD-9	295.4	Acute schizophrenic episode, unspecified state	
SCZ	295.1	ICD-9	295.41	Acute schizophrenic episode, subchronic state	
SCZ	295.1	ICD-9	295.42	Acute schizophrenic episode, chronic state	
SCZ	295.1	ICD-9	295.43	Acute schizophrenic episode, subchronic state with acute exacerbation	
SCZ	295.1	ICD-9	295.44	Acute schizophrenic episode, chronic state with acute exacerbation	
SCZ	295.1	ICD-9	295.45	Acute schizophrenic episode, in remission	
SCZ	295.1	ICD-9	295.5	Latent schizophrenia	
SCZ	295.1	ICD-9	295.5	Latent schizophrenia, unspecified state	
SCZ	295.1	ICD-9	295.51	Latent schizophrenia, subchronic state	
SCZ	295.1	ICD-9	295.52	Latent schizophrenia, chronic state	
SCZ	295.1	ICD-9	295.53	Latent schizophrenia, subchronic state with acute exacerbation	
SCZ	295.1	ICD-9	295.54	Latent schizophrenia, chronic state with acute exacerbation	
SCZ	295.1	ICD-9	295.55	Latent schizophrenia, in remission	
SCZ	295.1	ICD-9	295.6	Residual schizophrenia, unspecified state	
SCZ	295.1	ICD-9	295.6	Residual type schizophrenic disorders	
SCZ	295.1	ICD-9	295.61	Residual schizophrenia, subchronic state	
SCZ	295.1	ICD-9	295.62	Residual schizophrenia, chronic state	
SCZ	295.1	ICD-9	295.63	Residual schizophrenia, subchronic state with acute exacerbation	
SCZ	295.1	ICD-9	295.64	Residual schizophrenia, chronic state with acute exacerbation	
SCZ	295.1	ICD-9	295.65	Residual schizophrenia, in remission	
SCZ	295.1	ICD-9	295.7	Schizo-affective type schizophrenia, unspecified state	
SCZ	295.1	ICD-9	295.7	Schizoaffective disorder	
SCZ	295.1	ICD-9	295.71	Schizo-affective type schizophrenia, subchronic state	
SCZ	295.1	ICD-9	295.72	Schizo-affective type schizophrenia, chronic state	
SCZ	295.1	ICD-9	295.73	Schizo-affective type schizophrenia, subchronic state with acute exacerbation	
SCZ	295.1	ICD-9	295.74	Schizo-affective type schizophrenia, chronic state with acute exacerbation	
SCZ	295.1	ICD-9	295.75	Schizo-affective type schizophrenia, in remission	
SCZ	295.1	ICD-9	295.8	Other specified types of schizophrenia	
SCZ	295.1	ICD-9	295.8	Other specified types of schizophrenia, unspecified state	
SCZ	295.1	ICD-9	295.81	Other specified types of schizophrenia, subchronic state	

© 2022 Bigdeli TB et al. *JAMA Psychiatry.*

SCZ	295.1	ICD-9	295.82	Other specified types of schizophrenia, chronic state	
SCZ	295.1	ICD-9	295.83	Other specified types of schizophrenia, subchronic state with acute exacerbation	
SCZ	295.1	ICD-9	295.84	Other specified types of schizophrenia, chronic state with acute exacerbation	
SCZ	295.1	ICD-9	295.85	Other specified types of schizophrenia, in remission	
SCZ	295.1	ICD-9	295.9	Unspecified schizophrenia	
SCZ	295.1	ICD-9	295.9	Unspecified schizophrenia, unspecified state	
SCZ	295.1	ICD-9	295.91	Unspecified schizophrenia, subchronic state	
SCZ	295.1	ICD-9	295.92	Unspecified schizophrenia, chronic state	
SCZ	295.1	ICD-9	295.93	Unspecified schizophrenia, subchronic state with acute exacerbation	
SCZ	295.1	ICD-9	295.94	Unspecified schizophrenia, chronic state with acute exacerbation	
SCZ	295.1	ICD-9	295.95	Unspecified schizophrenia, in remission	
SCZ	295.1	ICD-9	V11.0	Personal history of schizophrenia	
SCZ	295.1	ICD-10	F20	Schizophrenia	
SCZ	295.1	ICD-10	F20.0	Paranoid schizophrenia	
SCZ	295.1	ICD-10	F20.1	Disorganized schizophrenia	
SCZ	295.1	ICD-10	F20.1	Hebephrenic schizophrenia	
SCZ	295.1	ICD-10	F20.2	Catatonic schizophrenia	
SCZ	295.1	ICD-10	F20.3	Undifferentiated schizophrenia	
SCZ	295.1	ICD-10	F20.5	Residual schizophrenia	
SCZ	295.1	ICD-10	F20.6	Simple schizophrenia	
SCZ	295.1	ICD-10	F20.8	Other schizophrenia	
SCZ	295.1	ICD-10	F20.81	Schizophreniform disorder	
SCZ	295.1	ICD-10	F20.89	Other schizophrenia	
SCZ	295.1	ICD-10	F20.9	Schizophrenia, unspecified	
SCZ	295.1	ICD-10	F25	Schizoaffective disorders	
SCZ	295.1	ICD-10	F25.0	Schizoaffective disorder, bipolar type	
SCZ	295.1	ICD-10	F25.0	Schizoaffective disorder, manic type	
SCZ	295.1	ICD-10	F25.1	Schizoaffective disorder, depressive type	
SCZ	295.1	ICD-10	F25.2	Schizoaffective disorder, mixed type	
SCZ	295.1	ICD-10	F25.8	Other schizoaffective disorders	
SCZ	295.1	ICD-10	F25.9	Schizoaffective disorder, unspecified	
Psychosis	295.3	ICD-9	293.81	Psychotic disorder with delusions in conditions classified elsewhere	
Psychosis	295.3	ICD-9	293.82	Psychotic disorder with hallucinations in conditions classified elsewhere	
Psychosis	295.3	ICD-9	298	Depressive type psychosis	
Psychosis	295.3	ICD-9	298	Other nonorganic psychoses	
Psychosis	295.3	ICD-9	298.1	Excitative type psychosis	
Psychosis	295.3	ICD-9	298.8	Other and unspecified reactive psychosis	
Psychosis	295.3	ICD-9	298.9	Unspecified psychosis	
Psychosis	295.3	ICD-9	298.9	Unspecified psychosis **	
Psychosis	295.3	ICD-10	F06.0	Organic hallucinosis	
Psychosis	295.3	ICD-10	F06.0	Psychotic disorder with hallucinations due to known physiological condition	
Psychosis	295.3	ICD-10	F06.2	Organic delusional [schizophrenia-like] disorder	
Psychosis	295.3	ICD-10	F06.2	Psychotic disorder with delusions due to known physiological condition	
Psychosis	295.3	ICD-10	F23.2	Acute schizophrenia-like psychotic disorder	
Psychosis	295.3	ICD-10	F28	Other psychotic disorder not due to a substance or known physiological condition	
Psychosis	295.3	ICD-10	F29	Unspecified nonorganic psychosis	
Psychosis	295.3	ICD-10	F29	Unspecified psychosis not due to a substance or known physiological condition	
Paranoid disorders	295.2	ICD-9	297	Paranoid state, simple	
Paranoid disorders	295.2	ICD-9	297	Paranoid states (Delusional disorders)	
Paranoid disorders	295.2	ICD-9	297.1	Delusional disorder	
Paranoid disorders	295.2	ICD-9	297.2	Paraphrenia	
Paranoid disorders	295.2	ICD-9	297.3	Shared psychotic disorder	
Paranoid disorders	295.2	ICD-9	297.8	Other specified paranoid states	
Paranoid disorders	295.2	ICD-9	297.9	Paranoid state NOS	
Paranoid disorders	295.2	ICD-9	298.3	Acute paranoid reaction	
Paranoid disorders	295.2	ICD-9	298.4	Psychogenic paranoid psychosis	
Paranoid disorders	295.2	ICD-10	F22	Delusional disorders	
Paranoid disorders	295.2	ICD-10	F22	Persistent delusional disorders	
Paranoid disorders	295.2	ICD-10	F22.0	Delusional disorder	
Paranoid disorders	295.2	ICD-10	F22.8	Other persistent delusional disorders	
Paranoid disorders	295.2	ICD-10	F22.9	Persistent delusional disorder, unspecified	
Paranoid disorders	295.2	ICD-10	F23	Brief psychotic disorder	

© 2022 Bigdeli TB et al. *JAMA Psychiatry*.

Paranoid disorders	295.2	ICD-10	F23.3	Other acute predominantly delusional psychotic disorders	
Paranoid disorders	295.2	ICD-10	F24	Induced delusional disorder	
Paranoid disorders	295.2	ICD-10	F24	Shared psychotic disorder	
BIP	296.1	ICD-9	296	Bipolar I disorder, single manic episode	
BIP	296.1	ICD-9	296	Manic disorder, single episode, unspecified degree	
BIP	296.1	ICD-9	296.01	Manic disorder, single episode, mild degree	
BIP	296.1	ICD-9	296.02	Manic disorder, single episode, moderate degree	
BIP	296.1	ICD-9	296.03	Manic disorder, single episode, severe, without mention of psychotic behavior	
BIP	296.1	ICD-9	296.04	Manic disorder, single episode, severe, specified as with psychotic behavior	
BIP	296.1	ICD-9	296.05	Manic disorder, single episode, in partial or unspecified remission	
BIP	296.1	ICD-9	296.06	Manic disorder, single episode, in full remission	
BIP	296.1	ICD-9	296.1	Manic disorder, recurrent episode	
BIP	296.1	ICD-9	296.1	Manic disorder, recurrent episode, unspecified degree	
BIP	296.1	ICD-9	296.11	Manic disorder, recurrent episode, mild degree	
BIP	296.1	ICD-9	296.12	Manic disorder, recurrent episode, moderate degree	
BIP	296.1	ICD-9	296.13	Manic disorder, recurrent episode, severe, without mention of psychotic behavior	
BIP	296.1	ICD-9	296.14	Manic disorder, recurrent episode, severe, specified as with psychotic behavior	
BIP	296.1	ICD-9	296.15	Manic disorder, recurrent episode, in partial or unspecified remission	
BIP	296.1	ICD-9	296.16	Manic disorder, recurrent episode, in full remission	
BIP	296.1	ICD-9	296.4	Bipolar affective disorder, manic, unspecified degree	
BIP	296.1	ICD-9	296.4	Bipolar I disorder, most recent episode (or current) manic	
BIP	296.1	ICD-9	296.41	Bipolar affective disorder, manic, mild degree	
BIP	296.1	ICD-9	296.42	Bipolar affective disorder, manic, moderate degree	
BIP	296.1	ICD-9	296.43	Bipolar affective disorder, manic, severe, without mention of psychotic behavior	
BIP	296.1	ICD-9	296.44	Bipolar affective disorder, manic, severe, specified as with psychotic behavior	
BIP	296.1	ICD-9	296.45	Bipolar affective disorder, manic, in partial or unspecified remission	
BIP	296.1	ICD-9	296.46	Bipolar affective disorder, manic, in full remission	
BIP	296.1	ICD-9	296.5	Bipolar affective disorder, depressed, unspecified degree	
BIP	296.1	ICD-9	296.5	Bipolar I disorder, most recent episode (or current) depressed	
BIP	296.1	ICD-9	296.51	Bipolar affective disorder, depressed, mild degree	
BIP	296.1	ICD-9	296.52	Bipolar affective disorder, depressed, moderate degree	
BIP	296.1	ICD-9	296.53	Bipolar affective disorder, depressed, severe, without mention of psychotic behavior	
BIP	296.1	ICD-9	296.54	Bipolar affective disorder, depressed, severe, specified as with psychotic behavior	
BIP	296.1	ICD-9	296.55	Bipolar affective disorder, depressed, in partial or unspecified remission	
BIP	296.1	ICD-9	296.56	Bipolar affective disorder, depressed, in full remission	
BIP	296.1	ICD-9	296.6	Bipolar affective disorder, mixed, unspecified degree	
BIP	296.1	ICD-9	296.6	Bipolar I disorder, most recent episode (or current) mixed	
BIP	296.1	ICD-9	296.61	Bipolar affective disorder, mixed, mild degree	
BIP	296.1	ICD-9	296.62	Bipolar affective disorder, mixed, moderate degree	
BIP	296.1	ICD-9	296.63	Bipolar affective disorder, mixed, severe, without mention of psychotic behavior	
BIP	296.1	ICD-9	296.64	Bipolar affective disorder, mixed, severe, specified as with psychotic behavior	
BIP	296.1	ICD-9	296.65	Bipolar affective disorder, mixed, in partial or unspecified remission	
BIP	296.1	ICD-9	296.66	Bipolar affective disorder, mixed, in full remission	
BIP	296.1	ICD-9	296.7	Bipolar I disorder, most recent episode (or current) unspecified	
BIP	296.1	ICD-9	296.7	Bipolar I disorder, most recent episode (or current) unspecified **	
BIP	296.1	ICD-9	296.8	Bipolar disorder NOS	
BIP	296.1	ICD-9	296.8	Other and unspecified bipolar disorders	
BIP	296.1	ICD-9	296.81	Atypical manic disorder	
BIP	296.1	ICD-9	296.82	Atypical depressive disorder	
BIP	296.1	ICD-9	296.89	Bipolar disorder NEC	
BIP	296.1	ICD-10	F30	Manic episode	
BIP	296.1	ICD-10	F30.0	Hypomania	
BIP	296.1	ICD-10	F30.1	Mania without psychotic symptoms	
BIP	296.1	ICD-10	F30.1	Manic episode without psychotic symptoms	
BIP	296.1	ICD-10	F30.10	Manic episode without psychotic symptoms, unspecified	
BIP	296.1	ICD-10	F30.11	Manic episode without psychotic symptoms, mild	
BIP	296.1	ICD-10	F30.12	Manic episode without psychotic symptoms, moderate	
BIP	296.1	ICD-10	F30.13	Manic episode, severe, without psychotic symptoms	
BIP	296.1	ICD-10	F30.2	Mania with psychotic symptoms	
BIP	296.1	ICD-10	F30.2	Manic episode, severe with psychotic symptoms	
BIP	296.1	ICD-10	F30.3	Manic episode in partial remission	

© 2022 Bigdeli TB et al. *JAMA Psychiatry*.

BIP	296.1	ICD-10	F30.4	Manic episode in full remission	
BIP	296.1	ICD-10	F30.8	Other manic episodes	
BIP	296.1	ICD-10	F30.9	Manic episode, unspecified	
BIP	296.1	ICD-10	F31	Bipolar affective disorder	
BIP	296.1	ICD-10	F31	Bipolar disorder	
BIP	296.1	ICD-10	F31.0	Bipolar affective disorder, current episode hypomanic	
BIP	296.1	ICD-10	F31.0	Bipolar disorder, current episode hypomanic	
BIP	296.1	ICD-10	F31.1	Bipolar affective disorder, current episode manic without psychotic symptoms	
BIP	296.1	ICD-10	F31.1	Bipolar disorder, current episode manic without psychotic features	
BIP	296.1	ICD-10	F31.10	Bipolar disorder, current episode manic without psychotic features, unspecified	
BIP	296.1	ICD-10	F31.11	Bipolar disorder, current episode manic without psychotic features, mild	
BIP	296.1	ICD-10	F31.12	Bipolar disorder, current episode manic without psychotic features, moderate	
BIP	296.1	ICD-10	F31.13	Bipolar disorder, current episode manic without psychotic features, severe	
BIP	296.1	ICD-10	F31.2	Bipolar affective disorder, current episode manic with psychotic symptoms	
BIP	296.1	ICD-10	F31.2	Bipolar disorder, current episode manic severe with psychotic features	
BIP	296.1	ICD-10	F31.3	Bipolar affective disorder, current episode mild or moderate depression	
BIP	296.1	ICD-10	F31.3	Bipolar disorder, current episode depressed, mild or moderate severity	
BIP	296.1	ICD-10	F31.30	Bipolar disorder, current episode depressed, mild or moderate severity, unspecified	
BIP	296.1	ICD-10	F31.31	Bipolar disorder, current episode depressed, mild	
BIP	296.1	ICD-10	F31.32	Bipolar disorder, current episode depressed, moderate	
BIP	296.1	ICD-10	F31.4	Bipolar affective disorder, current episode severe depression without psychosis	
BIP	296.1	ICD-10	F31.4	Bipolar disorder, current episode depressed, severe, without psychotic features	
BIP	296.1	ICD-10	F31.5	Bipolar affective disorder, current episode severe depression with psychosis	
BIP	296.1	ICD-10	F31.5	Bipolar disorder, current episode depressed, severe, with psychotic features	
BIP	296.1	ICD-10	F31.6	Bipolar affective disorder, current episode mixed	
BIP	296.1	ICD-10	F31.6	Bipolar disorder, current episode mixed	
BIP	296.1	ICD-10	F31.60	Bipolar disorder, current episode mixed, unspecified	
BIP	296.1	ICD-10	F31.61	Bipolar disorder, current episode mixed, mild	
BIP	296.1	ICD-10	F31.62	Bipolar disorder, current episode mixed, moderate	
BIP	296.1	ICD-10	F31.63	Bipolar disorder, current episode mixed, severe, without psychotic features	
BIP	296.1	ICD-10	F31.64	Bipolar disorder, current episode mixed, severe, with psychotic features	
BIP	296.1	ICD-10	F31.7	Bipolar affective disorder, currently in remission	
BIP	296.1	ICD-10	F31.7	Bipolar disorder, currently in remission	
BIP	296.1	ICD-10	F31.70	Bipolar disorder, currently in remission, most recent episode unspecified	
BIP	296.1	ICD-10	F31.71	Bipolar disorder, in partial remission, most recent episode hypomanic	
BIP	296.1	ICD-10	F31.72	Bipolar disorder, in full remission, most recent episode hypomanic	
BIP	296.1	ICD-10	F31.73	Bipolar disorder, in partial remission, most recent episode manic	
BIP	296.1	ICD-10	F31.74	Bipolar disorder, in full remission, most recent episode manic	
BIP	296.1	ICD-10	F31.75	Bipolar disorder, in partial remission, most recent episode depressed	
BIP	296.1	ICD-10	F31.76	Bipolar disorder, in full remission, most recent episode depressed	
BIP	296.1	ICD-10	F31.77	Bipolar disorder, in partial remission, most recent episode mixed	
BIP	296.1	ICD-10	F31.78	Bipolar disorder, in full remission, most recent episode mixed	
BIP	296.1	ICD-10	F31.8	Other bipolar affective disorders	
BIP	296.1	ICD-10	F31.8	Other bipolar disorders	
BIP	296.1	ICD-10	F31.81	Bipolar II disorder	
BIP	296.1	ICD-10	F31.89	Other bipolar disorder	
BIP	296.1	ICD-10	F31.9	Bipolar affective disorder, unspecified	
BIP	296.1	ICD-10	F31.9	Bipolar disorder, unspecified	
BIP	296.1	ICD-10	F32.8	Other depressive episodes	
BIP	296.1	ICD-10	F32.81	Premenstrual dysphoric disorder	
DEP	296.22	ICD-9	296.2	Major depressive disorder, single episode	
DEP	296.22	ICD-9	296.2	Major depressive disorder, single episode, unspecified degree	
DEP	296.22	ICD-9	296.22	Major depressive disorder, single episode, moderate degree	
DEP	296.22	ICD-9	296.23	Major depressive disorder, single episode, severe degree, without psychosis	
DEP	296.22	ICD-9	296.24	Major depressive disorder, single episode, severe degree, with psychotic behavior	
DEP	296.22	ICD-9	296.25	Major depressive disorder, single episode, in partial or unspecified remission	
DEP	296.22	ICD-9	296.26	Major depressive disorder, single episode in full remission	
DEP	296.22	ICD-9	296.3	Major depressive disorder, recurrent episode	
DEP	296.22	ICD-9	296.3	Major depressive disorder, recurrent episode, unspecified degree	
DEP	296.22	ICD-9	296.32	Major depressive disorder, recurrent episode, moderate degree	
DEP	296.22	ICD-9	296.33	Major depressive disorder, recurrent episode, severe degree, without psychosis	

© 2022 Bigdeli TB et al. *JAMA Psychiatry*.

DEP	296.22	ICD-9	296.34	Major depressive disorder, recurrent episode, severe degree, with psychosis	
DEP	296.22	ICD-9	296.35	Major depressive disorder, recurrent episode, in partial or unspecified remission	
DEP	296.22	ICD-9	296.36	Major depressive disorder, recurrent episode, in full remission	
DEP	296.22	ICD-10	F20.4	Post-schizophrenic depression	
DEP	296.22	ICD-10	F32	Major depressive disorder, single episode	
DEP	296.22	ICD-10	F32.0	Major depressive disorder, single episode, mild	
DEP	296.22	ICD-10	F32.1	Major depressive disorder, single episode, moderate	
DEP	296.22	ICD-10	F32.2	Major depressive disorder, single episode, severe without psychotic features	
DEP	296.22	ICD-10	F32.3	Major depressive disorder, single episode, severe with psychotic features	
DEP	296.22	ICD-10	F32.4	Major depressive disorder, single episode, in partial remission	
DEP	296.22	ICD-10	F32.5	Major depressive disorder, single episode, in full remission	
DEP	296.22	ICD-10	F32.8	Other depressive episodes	
DEP	296.22	ICD-10	F32.81	Premenstrual dysphoric disorder	
DEP	296.22	ICD-10	F32.89	Single episode of 'masked' depression NOS	
DEP	296.22	ICD-10	F32.9	Major depressive disorder, single episode, unspecified	
DEP	296.22	ICD-10	F33	Major depressive disorder, recurrent	
DEP	296.22	ICD-10	F33.0	Major depressive disorder, recurrent, mild	
DEP	296.22	ICD-10	F33.0	Recurrent depressive disorder, current episode mild	
DEP	296.22	ICD-10	F33.1	Major depressive disorder, recurrent, moderate	
DEP	296.22	ICD-10	F33.1	Recurrent depressive disorder, current episode moderate	
DEP	296.22	ICD-10	F33.2	Major depressive disorder, recurrent severe without psychotic features	
DEP	296.22	ICD-10	F33.2	Recurrent depressive disorder, current episode severe without psychosis	
DEP	296.22	ICD-10	F33.3	Major depressive disorder, recurrent, severe with psychotic symptoms	
DEP	296.22	ICD-10	F33.3	Recurrent depressive disorder, current episode severe with psychotic symptoms	
DEP	296.22	ICD-10	F33.4	Major depressive disorder, recurrent, in remission	
DEP	296.22	ICD-10	F33.4	Recurrent depressive disorder, currently in remission	
DEP	296.22	ICD-10	F33.40	Major depressive disorder, recurrent, in remission, unspecified	
DEP	296.22	ICD-10	F33.41	Major depressive disorder, recurrent, in partial remission	
DEP	296.22	ICD-10	F33.42	Major depressive disorder, recurrent, in full remission	
DEP	296.22	ICD-10	F33.8	Other recurrent depressive disorders	
DEP	296.22	ICD-10	F33.9	Major depressive disorder, recurrent, unspecified	
DEP	296.2	ICD-9	296.21	Major depressive disorder, single episode, mild degree	
DEP	296.2	ICD-9	296.31	Major depressive disorder, recurrent episode, mild degree	
DEP	296.2	ICD-9	311	Depressive disorder NEC	
SCZ and other psychotic disorders	295	ICD-9	295	Schizophrenic disorders	Used for control screening
SCZ and other psychotic disorders	295	ICD-9	295.4	Schizophreniform disorder	Used for control screening
SCZ and other psychotic disorders	295	ICD-9	298.2	Reactive confusion	Used for control screening
SCZ and other psychotic disorders	295	ICD-10	F44.3	Trance and possession disorders	Used for control screening
SCZ and other psychotic disorders	295	ICD-10	F44.8	Other dissociative [conversion] disorders	Used for control screening
SCZ and other psychotic disorders	295	ICD-10	F44.89	Other dissociative and conversion disorders	Used for control screening
Mood disorders	296	ICD-9	293.83	Mood disorder in conditions classified elsewhere	Used for control screening
Mood disorders	296	ICD-9	296	Episodic mood disorders	Used for control screening
Mood disorders	296	ICD-9	296.9	Other and unspecified episodic mood disorder	Used for control screening
Mood disorders	296	ICD-9	296.9	Unspecified episodic mood disorder	Used for control screening
Mood disorders	296	ICD-9	296.99	Other specified episodic mood disorder	Used for control screening
Mood disorders	296	ICD-9	V11.1	Personal history of affective disorders	Used for control screening
Mood disorders	296	ICD-10	F06.3	Mood disorder due to known physiological condition	Used for control screening
Mood disorders	296	ICD-10	F06.3	Organic mood [affective] disorders	Used for control screening
Mood disorders	296	ICD-10	F06.30	Mood disorder due to known physiological condition, unspecified	Used for control screening
Mood disorders	296	ICD-10	F06.31	Mood disorder due to known physiological condition with depressive features	Used for control screening
Mood disorders	296	ICD-10	F06.32	Mood disorder due to known physiological condition with major depressive-like episode	Used for control screening
Mood disorders	296	ICD-10	F06.33	Mood disorder due to known physiological condition with manic features	Used for control screening
Mood disorders	296	ICD-10	F06.34	Mood disorder due to known physiological condition with mixed features	Used for control screening
Mood disorders	296	ICD-10	F34	Persistent mood [affective] disorders	Used for control screening
Mood disorders	296	ICD-10	F34.8	Other persistent mood [affective] disorders	Used for control screening
Mood disorders	296	ICD-10	F34.81	Disruptive mood dysregulation disorder	Used for control screening
Mood disorders	296	ICD-10	F34.89	Other specified persistent mood disorders	Used for control screening
Mood disorders	296	ICD-10	F34.9	Persistent mood [affective] disorder, unspecified	Used for control screening
Mood disorders	296	ICD-10	F38.0	Other single mood [affective] disorders	Used for control screening

© 2022 Bigdeli TB et al. *JAMA Psychiatry*.

Mood disorders	296	ICD-10	F38.1	Other recurrent mood [affective] disorders	Used for control screening
Mood disorders	296	ICD-10	F38.8	Other specified mood [affective] disorders	Used for control screening
Mood disorders	296	ICD-10	F39	Unspecified mood [affective] disorder	Used for control screening

eTable 2. Medications queried from VHA prescription records.

We compiled lists of commonly prescribed antipsychotics, mood stabilizers, and antidepressants from the VHA national formulary and prescription records for CSP #572, which we subsequently extracted from all MVP participants' prescription records.

Antipsychotics		Mood stabilizers	Antidepressants	
aripiprazole	ziprasidone	carbamazepine	Amitriptyline	Sertraline
asenapine	olanzapine	divalproex	Buproprion	Trazadone
cariprazine	loxapine	lamotrigine	Citalopram	Venlafaxine
chlorpromazine	molindone	lithium	Desipramine	Vortioxetine
clozapine	thioridazine	valproate	Desvenlafaxine	Doxepin
fluphenazine	trifluoperazine	gabapentin	Duloxetine	Mirtazapine
haloperidol	brexpiprazole	lacosamide	Escitalopram	Nortriptyline
Iloperidone	zuclopentixol	topiramate	Fluoxetine	Tranylcypromine
lurasidone	paliperidone	zonisamide	Fluvoxamine	trimipramine
perphenazine	sertindole	riluzole	Imipramine	Bupropion
pimozide	mesoridazine	oxcarbazepine	Paroxetine	Nefazodone
quetiapine	thiothixene	valproic acid	Phenelzine	Selegiline
risperidone	zuclopenthixol		Selegilene	Trazodone

© 2022 Bigdeli TB et al. *JAMA Psychiatry*.

eTable 3. CSP #572 and MVP participants meeting varying EHR-based criteria (females only).

		Cooperative Studies Program (CSP) #572						Million Veteran Program (MVP)*		
		Schizophrenia			Bipolar I disorder					
No. Female participants		289			1,005			62,749		
Median age (SD)		52 (10.7)			49 (10.7)			52 (13.6)		
Diagnosis	Phecode	No. Participants (%)								
		≥1 ICD-9/10	≥2 ICD-9/10	Inpatient	≥1 ICD-9/10	≥2 ICD-9/10	Inpatient	≥1 ICD-9/10	≥2 ICD-9/10	Inpatient
Schizophrenia	295.1	270 (93.4)	268 (92.7)	214 (74.0)	294 (29.3)	221 (22.0)	128 (12.7)	2,669 (4.3)	1,925 (3.1)	1,150 (1.8)
Paranoid	*295.2*	*+1 (0.3)*	*+1 (0.3)*	*+0 (0.0)*	*+31 (3.1)*	*+19 (1.9)*	*+12 (1.2)*	*+452 (0.7)*	*+260 (0.4)*	*+127 (0.2)*
Psychosis	*295.3*	*+3 (1.0)*	*+4 (1.4)*	*+7 (2.4)*	*+80 (8.0)*	*+59 (5.9)*	*+38 (3.8)*	*+1,761 (2.8)*	*+1,009 (1.6)*	*+436 (0.7)*
		274 (94.8)	273 (94.5)	221 (76.5)	405 (40.3)	299 (29.8)	178 (17.7)	4,882 (7.8)	3,194 (5.1)	1,713 (2.7)
Bipolar disorder (mania)	296.1	61 (21.1)	50 (17.3)	26 (9.0)	590 (58.7)	533 (53.0)	179 (17.8)	2,814 (4.5)	1,781 (2.8)	599 (1.0)
Bipolar disorder (any)	*296.1*	*+94 (32.5)*	*+67 (23.2)*	*+64 (22.1)*	*+384 (38.2)*	*+438 (43.6)*	*+501 (49.9)*	*+9,892 (15.8)*	*+7,473 (11.9)*	*+3,372 (5.4)*
		155 (53.6)	117 (40.5)	90 (31.1)	974 (96.9)	971 (96.6)	680 (67.7)	12,706 (20.2)	9,254 (14.7)	3,971 (6.3)
Depression	296.2	240 (83.0)	210 (72.7)	143 (49.5)	857 (85.3)	784 (78.0)	382 (38.0)	42,253 (67.3)	38,489 (61.3)	14,803 (23.6)
Treatment		≥1 Rx	≥2 trials		≥1 Rx	≥2 trials		≥1 Rx	≥2 trials	
antipsychotics		271 (93.8)	248 (85.8)		900 (89.6)	751 (74.7)		13,999 (22.3)	7,236 (11.5)	
mood stabilizers		200 (69.2)	114 (39.4)		951 (94.6)	806 (80.2)		33,423 (53.3)	13,467 (21.5)	
antidepressants		223 (77.2)	175 (60.6)		871 (86.7)	719 (71.5)		43,527 (69.4)	30,353 (48.4)	

*Participants dually enrolled in CSP #572 and MVP were excluded.

© 2022 Bigdeli TB et al. *JAMA Psychiatry*.

eTable 4. Factors associated with diagnostic misclassification of BPI cases (as SCZ) in CSP #572.

For each predictor, *β* and *SE* are the regression coefficient and its standard error; OR is the corresponding odds ratio with its 95% CI displayed, and *p*-value is the significance of each predictor in the logistic model.

Predictor	*β*	SE	OR (95% CI)	*p*-value
race:AA	0.809	0.103	2.25 (1.84, 2.74)	3.18E-15
race:other non-EA	0.518	0.195	1.68 (1.13, 2.43)	7.98E-03
Sex	-0.469	0.139	0.63 (0.47, 0.82)	7.43E-04
Age	-0.006	0.004	0.99 (0.99, 1.00)	2.01E-01
AUD	-0.251	0.213	0.78 (0.52, 1.20)	2.38E-01
SUD (any)	0.219	0.199	1.25 (0.83, 1.81)	2.71E-01
UPSA-Brief	-0.094	0.019	0.91 (0.88, 0.94)	4.42E-07

eTable 5. Factors associated with diagnostic misclassification of SCZ cases (as BPI) in CSP #572.

For each predictor, *β* and *SE* are the regression coefficient and its standard error; OR is the corresponding odds ratio with its 95% CI displayed, and *p*-value is the significance of each predictor in the logistic model.

Predictor	*β*	SE	OR (95% CI)	*p*-value
race:AA	0.139	0.211	1.15 (0.76, 1.75)	5.11E-01
race:other non-EA	0.699	0.342	1.15 (0.76, 1.75)	4.12E-02
Sex	0.728	0.278	1.15 (0.76, 1.75)	8.88E-03
Age	-0.008	0.009	1.15 (0.76, 1.75)	3.88E-01
AUD	-0.530	0.435	1.15 (0.76, 1.75)	2.23E-01
SUD (any)	0.169	0.380	1.15 (0.76, 1.75)	6.57E-01
UPSA-Brief	0.080	0.035	1.15 (0.76, 1.75)	2.34E-02

eTable 6. Predictive models for SCZ or BPI diagnosis based on EHR.

For split-half, training/testing experiments in CSP #572, predictive models for SCZ (the outcome) versus BPI, and for BPI (the outcome) versus SCZ are shown. Sensitivity, specific, and area-under-the-curve (AUC) estimates are reported for varying probability thresholds for assigning case status.

Outcome	Predictor	Probability threshold for assigning case status								
		p(case)>0.5			p(case)>0.7			p(case)>0.9		
		Sensitivity	Specificity	AUC	Sensitivity	Specificity	AUC	Sensitivity	Specificity	AUC
SCZ	1 ICD-9/10	0.697	0.961	0.829	0.914	0.565	0.740	1.000	0.000	0.500
	2 ICD-9/10	0.772	0.955	0.864	0.889	0.666	0.777	1.000	0.000	0.500
	Inpatient code	0.868	0.766	0.817	0.913	0.651	0.782	0.997	0.051	0.524
	Antipsychotics	0.820	0.564	0.692	0.960	0.218	0.589	1.000	0.000	0.500
BPI	1 ICD-9/10	0.989	0.562	0.775	0.990	0.560	0.775	0.991	0.549	0.770
	2 ICD-9/10	0.981	0.699	0.840	0.981	0.699	0.840	0.983	0.674	0.828
	Inpatient code	0.763	0.763	0.763	0.949	0.481	0.715	1.000	0.000	0.500

© 2022 Bigdeli TB et al. *JAMA Psychiatry*.

	mood-stabilizers	0.813	0.666	0.739	0.971	0.302	0.636	0.991	0.171	0.581

© 2022 Bigdeli TB et al. *JAMA Psychiatry*.

eTable 7. Associations of neuropsychiatric PRSs with SCZ.
For each training dataset and target population in MVP, p_T is the p-value threshold applied to its results; $R^2_{\text{Nagelkerke}}$ and $R^2_{\text{liability}}$ are the variance explained in terms of Nagelkerke's R^2 and on the liability scale (for prevalences, K, of 0.01, 0.02, and 0.04), respectively; β and *SE* are the regression coefficient and its standard error; OR is the corresponding odds ratio and its 95% CI, and *p*-value is the significance of the PRS in the logistic model.

Training Data	Population	*p*T	$R^2_{\text{Nagelkerke}}$	$R^2_{\text{liability}}$			β	SE	OR (95% CI)	*p*-value
				K=0.01	K=0.02	K=0.04				
PGC3-SCZ	EA	0.5	0.041	0.042	0.050	0.060	0.595	0.016	1.813 (1.757, 1.871)	3.7E-298
PGC3-SCZ	EA	0.05	0.043	0.044	0.052	0.062	0.602	0.016	1.825 (1.769, 1.884)	<1E-300
PGC3-SCZ	EA	5E-08	0.014	0.014	0.016	0.020	0.330	0.015	1.392 (1.35 , 1.434)	1.3E-101
PGC3-BIP	EA	0.5	0.016	0.016	0.019	0.023	0.375	0.016	1.456 (1.41 , 1.503)	4.9E-119
PGC3-BIP	EA	0.05	0.015	0.016	0.018	0.022	0.370	0.016	1.448 (1.402, 1.495)	8.8E-115
PGC3-BIP	EA	5E-08	0.010	0.011	0.013	0.015	0.298	0.016	1.348 (1.307, 1.39)	5.1E-79
PGC-MDD(2018)	EA	0.5	0.007	0.007	0.009	0.011	0.246	0.016	1.279 (1.24 , 1.318)	8.0E-56
PGC-MDD(2018)	EA	0.05	0.006	0.007	0.008	0.009	0.230	0.016	1.259 (1.221, 1.298)	1.4E-49
PGC-MDD(2018)	EA	5E-08	0.000	0.000	0.000	0.000	0.046	0.015	1.047 (1.016, 1.079)	2.5E-03
PGC3-SCZ	AA	0.5	0.012	0.006	0.008	0.009	0.303	0.023	1.354 (1.294, 1.417)	5.7E-39
PGC3-SCZ	AA	0.05	0.012	0.006	0.008	0.009	0.286	0.022	1.331 (1.276, 1.389)	2.4E-39
PGC3-SCZ	AA	5E-08	0.003	0.002	0.002	0.002	0.120	0.018	1.127 (1.089, 1.167)	1.2E-11
PGC3-BIP	AA	0.5	0.004	0.002	0.003	0.003	0.170	0.022	1.185 (1.136, 1.236)	3.4E-15
PGC3-BIP	AA	0.05	0.004	0.002	0.002	0.003	0.143	0.020	1.153 (1.109, 1.199)	6.4E-13
PGC3-BIP	AA	5E-08	0.000	0.000	0.000	0.000	0.040	0.018	1.04 (1.005, 1.077)	2.4E-02
PGC-MDD(2018)	AA	0.5	0.000	0.000	0.000	0.000	0.053	0.025	1.054 (1.005, 1.106)	3.1E-02
PGC-MDD(2018)	AA	0.05	0.000	0.000	0.000	0.000	0.055	0.025	1.056 (1.006, 1.108)	2.7E-02
PGC-MDD(2018)	AA	5E-08	0.000	0.000	0.000	0.000	0.012	0.018	1.012 (0.977, 1.047)	5.1E-01

© 2022 Bigdeli TB et al. *JAMA Psychiatry*.

eTable 8. Associations of neuropsychiatric PRSs with BIP.

For each training dataset and target population in MVP, p_T is the p-value threshold applied to its results; $R^2_{\text{Nagelkerke}}$ and $R^2_{\text{liability}}$ are the variance explained in terms of Nagelkerke's R^2 and on the liability scale (for prevalences, K, of 0.01, 0.02, and 0.04), respectively; β and *SE* are the regression coefficient and its standard error; OR is the corresponding odds ratio and its 95% CI, and *p*-value is the significance of the PRS in the logistic model.

Training Data	Population	pT	$R^2_{\text{Nagelkerke}}$	$R^2_{\text{liability}}$			β	SE	OR (95% CI)	*p*-value
				K=0.01	K=0.02	K=0.04				
PGC3-SCZ	EA	0.5	0.016	0.010	0.012	0.014	0.340	0.009	1.405 (1.38, 1.431)	7.8E-295
PGC3-SCZ	EA	0.05	0.017	0.011	0.012	0.015	0.347	0.009	1.415 (1.389, 1.44)	<1E-300
PGC3-SCZ	EA	5E-08	0.005	0.003	0.004	0.005	0.189	0.009	1.208 (1.187, 1.23)	2.3E-99
PGC3-BIP	EA	0.5	0.016	0.010	0.012	0.014	0.349	0.009	1.417 (1.391, 1.444)	5.0E-296
PGC3-BIP	EA	0.05	0.015	0.009	0.011	0.013	0.338	0.010	1.403 (1.377, 1.429)	4.0E-277
PGC3-BIP	EA	5E-08	0.011	0.007	0.008	0.010	0.288	0.009	1.334 (1.31, 1.359)	7.6E-211
PGC-MDD(2018)	EA	0.5	0.008	0.005	0.006	0.007	0.243	0.009	1.275 (1.252, 1.298)	8.7E-156
PGC-MDD(2018)	EA	0.05	0.007	0.004	0.005	0.006	0.225	0.009	1.253 (1.231, 1.275)	8.5E-136
PGC-MDD(2018)	EA	5E-08	0.000	0.000	0.000	0.000	0.046	0.009	1.047 (1.029, 1.065)	2.2E-07
PGC3-SCZ	AA	0.5	0.003	0.001	0.002	0.002	0.167	0.024	1.181 (1.127, 1.238)	3.2E-12
PGC3-SCZ	AA	0.05	0.004	0.002	0.002	0.003	0.172	0.022	1.187 (1.136, 1.241)	1.9E-14
PGC3-SCZ	AA	5E-08	0.001	0.001	0.001	0.001	0.085	0.018	1.089 (1.051, 1.128)	1.9E-06
PGC3-BIP	AA	0.5	0.003	0.001	0.002	0.002	0.145	0.022	1.156 (1.107, 1.207)	5.2E-11
PGC3-BIP	AA	0.05	0.002	0.001	0.001	0.002	0.118	0.020	1.125 (1.082, 1.171)	4.7E-09
PGC3-BIP	AA	5E-08	0.001	0.000	0.000	0.001	0.065	0.018	1.067 (1.03, 1.105)	2.8E-04
PGC-MDD(2018)	AA	0.5	0.000	0.000	0.000	0.000	0.000	0.026	1.000 (0.951, 1.051)	9.9E-01
PGC-MDD(2018)	AA	0.05	0.000	0.000	0.000	0.000	0.023	0.025	1.023 (0.973, 1.075)	3.8E-01
PGC-MDD(2018)	AA	5E-08	0.000	0.000	0.000	0.000	0.046	0.018	1.047 (1.011, 1.084)	9.6E-03

© 2022 Bigdeli TB et al. *JAMA Psychiatry*.

eTable 9. Associations of neuropsychiatric PRSs with DEP.

For each training dataset and target population in MVP, p_T is the p-value threshold applied to its results; R^2 Nagelkerke and $R^2_{liability}$ are the variance explained in terms of Nagelkerke's R^2 and on the liability scale (for prevalences, K, of 0.1, 0.4, and 0.5), respectively; *β* and *SE* are the regression coefficient and its standard error; OR is the corresponding odds ratio and its 95% CI, and *p* is the significance of the PRS in the logistic model.

Training Data	Population	pT	R^2 Nagelkerke	R^2 liability			*β*	SE	OR (95% CI)	*p*-value
				K=0.1	K=0.4	K=0.5				
PGC3-SCZ	EA	0.5	0.004	0.003	0.005	0.005	0.134	0.005	1.143 (1.133, 1.154)	7.2E-173
PGC3-SCZ	EA	0.05	0.004	0.003	0.005	0.005	0.131	0.005	1.14 (1.13 , 1.151)	1.2E-169
PGC3-SCZ	EA	5E-08	0.001	0.001	0.001	0.001	0.068	0.005	1.07 (1.061, 1.08)	1.0E-48
PGC3-BIP	EA	0.5	0.004	0.003	0.004	0.004	0.123	0.005	1.131 (1.120, 1.141)	5.3E-142
PGC3-BIP	EA	0.05	0.003	0.003	0.004	0.004	0.121	0.005	1.129 (1.118, 1.139)	7.6E-137
PGC3-BIP	EA	5E-08	0.002	0.002	0.003	0.003	0.101	0.005	1.106 (1.096, 1.117)	8.4E-99
PGC-MDD(2018)	EA	0.5	0.007	0.005	0.008	0.008	0.163	0.005	1.178 (1.167, 1.189)	9.4E-261
PGC-MDD(2018)	EA	0.05	0.006	0.005	0.007	0.007	0.156	0.005	1.169 (1.158, 1.18)	4.3E-240
PGC-MDD(2018)	EA	5E-08	0.000	0.000	0.000	0.000	0.034	0.005	1.034 (1.025, 1.044)	3.3E-13
PGC3-SCZ	AA	0.5	0.002	0.001	0.002	0.002	0.106	0.014	1.112 (1.082, 1.142)	6.6E-15
PGC3-SCZ	AA	0.05	0.002	0.001	0.002	0.002	0.098	0.013	1.103 (1.076, 1.131)	1.3E-14
PGC3-SCZ	AA	5E-08	0.000	0.000	0.000	0.000	0.029	0.010	1.03 (1.010, 1.05)	3.7E-03
PGC3-BIP	AA	0.5	0.001	0.001	0.001	0.001	0.059	0.013	1.061 (1.035, 1.087)	2.7E-06
PGC3-BIP	AA	0.05	0.000	0.000	0.000	0.001	0.044	0.012	1.045 (1.022, 1.069)	1.2E-04
PGC3-BIP	AA	5E-08	0.000	0.000	0.000	0.000	0.018	0.010	1.018 (0.998, 1.039)	7.2E-02
PGC-MDD(2018)	AA	0.5	0.000	0.000	0.000	0.000	0.026	0.014	1.026 (0.998, 1.055)	7.2E-02
PGC-MDD(2018)	AA	0.05	0.000	0.000	0.000	0.000	0.023	0.014	1.023 (0.995, 1.052)	1.1E-01
PGC-MDD(2018)	AA	5E-08	0.000	0.000	0.000	0.000	0.023	0.010	1.023 (1.003, 1.043)	2.5E-02

© 2022 Bigdeli TB et al. *JAMA Psychiatry*.

eTable 10. Associations of neuropsychiatric PRSs with total comorbidity burden among EA participants.

Effect sizes and significance from negative binomial regression are reported for six models: (i) all individuals, irrespective of diagnostic status, and adjusting for sex, age, age^2, and ancestry PCs; (ii) excluding individuals with psychotic disorders; (iii) excluding individuals with affective disorders; (iv) excluding individuals who were treated with antipsychotics; (v) individuals who were treated with mood stabilizers; and (vi) individuals who were treated with antidepressants. Odds ratios are per SD unit increase in PRSs.

PRS	All participants			Exclude phecode 295.*			Exclude phecode 296.*			Exclude antipsychotics			Exclude mood-stabilizers			Exclude antidepressants		
	β	SE	*p*-value	*β*	SE	*p*-value	*β*	SE	*p*-value	*β*	SE	p	*β*	SE	*p*-value	*β*	SE	*p*-value
PGC3-SCZ	0.019	0.001	1.6E-59	0.013	0.001	3.5E-25	0.007	0.002	2.8E-05	0.009	0.001	4.9E-12	0.015	0.001	1.4E-30	0.013	0.002	1.5E-13
PGC3-BIP	0.021	0.001	3.2E-66	0.016	0.001	5.1E-37	0.005	0.002	7.0E-03	0.012	0.001	9.6E-19	0.015	0.001	3.3E-29	0.010	0.002	2.7E-08
PGC-MDD	0.036	0.001	3.4E-196	0.033	0.001	8.8E-155	0.021	0.002	3.1E-35	0.029	0.001	8.0E-105	0.030	0.001	9.8E-120	0.022	0.002	5.7E-35
gSEM-BIP	-0.013	0.001	6.3E-29	-0.013	0.001	2.9E-26	-0.012	0.002	7.3E-13	-0.011	0.001	2.3E-18	-0.013	0.001	2.2E-24	-0.009	0.002	1.0E-07
gSEM-MDD	0.045	0.001	5.4E-308	0.040	0.001	3.8E-228	0.022	0.002	3.0E-37	0.034	0.001	4.5E-142	0.037	0.001	1.7E-177	0.024	0.002	3.6E-41
gSEM-GEN	0.024	0.001	1.1E-91	0.026	0.001	3.0E-103	0.018	0.002	1.1E-25	0.024	0.001	2.0E-76	0.022	0.001	1.0E-66	0.012	0.002	2.4E-11
gSEM-SCZ	-0.013	0.001	1.0E-29	-0.016	0.001	8.5E-40	-0.008	0.002	1.2E-05	-0.014	0.001	1.2E-28	-0.011	0.001	7.6E-19	-0.003	0.002	9.3E-02

eTable 11. Associations of neuropsychiatric PRSs with total comorbidity burden among AA participants.

Effect sizes and significance from negative binomial regression are reported for six models: (i) all individuals, irrespective of diagnostic status, and adjusting for sex, age, age^2, and ancestry PCs; (ii) excluding individuals with psychotic disorders; (iii) excluding individuals with affective disorders; (iv) excluding individuals who were treated with antipsychotics; (v) individuals who were treated with mood stabilizers; and (vi) individuals who were treated with antidepressants. Odds ratios are per SD unit increase in PRSs.

PRS	All participants			Exclude phecode 295.*			Exclude phecode 296.*			Exclude antipsychotics			Exclude mood-stabilizers			Exclude antidepressants		
	β	SE	*p*-value	*β*	SE	*p*-value	*β*	SE	*p*-value	*β*	SE	*p*-value	*β*	SE	*p*-value	*β*	SE	*p*-value
PGC3-SCZ	0.011	0.003	9.6E-06	0.010	0.003	5.0E-04	0.009	0.004	3.2E-02	0.008	0.003	8.6E-03	0.010	0.003	2.3E-04	0.010	0.004	1.9E-02
PGC3-BIP	0.016	0.003	6.6E-10	0.013	0.003	1.2E-06	0.013	0.004	1.8E-03	0.010	0.003	6.9E-04	0.013	0.003	2.0E-06	0.017	0.004	7.2E-05
PGC-MDD	0.004	0.003	1.5E-01	0.004	0.003	1.8E-01	0.007	0.005	1.7E-01	0.005	0.003	1.2E-01	0.003	0.003	3.8E-01	0.004	0.005	4.4E-01
gSEM-BIP	0.001	0.002	6.1E-01	0.000	0.002	9.0E-01	0.002	0.003	6.1E-01	-0.001	0.002	7.4E-01	0.000	0.002	9.7E-01	0.005	0.003	1.2E-01
gSEM-MDD	0.025	0.002	3.6E-24	0.022	0.003	4.5E-16	0.017	0.004	1.1E-04	0.018	0.003	2.1E-09	0.020	0.003	6.2E-13	0.015	0.004	3.9E-04
gSEM-GEN	0.006	0.002	1.3E-02	0.005	0.002	4.7E-02	0.001	0.004	8.5E-01	0.006	0.003	3.6E-02	0.003	0.003	2.2E-01	-0.006	0.004	1.4E-01
gSEM-SCZ	-0.006	0.002	9.9E-03	-0.005	0.003	7.3E-02	0.001	0.004	9.0E-01	-0.004	0.003	1.3E-01	-0.003	0.003	2.2E-01	0.003	0.004	5.1E-01

© 2022 Bigdeli TB et al. *JAMA Psychiatry*.

eTable 12. Associations of neuropsychiatric PRSs with inpatient treatment for SCZ.

Among EA or AA participants with a diagnosis of SCZ, primary and gSEM-derived PRSs were tested for association with ever having received inpatient treatment for SCZ (i.e. by logistic regression). Effect sizes and significance are displayed for two models: (i) adjusting for total number of comorbidities, sex, age, age^2, and ancestry PCs; (ii) adjusting for any lifetime diagnosis of SUD (phecode 316) or AUD (phecode 317) (separately), sex, age, age-squared, and ancestry PCs. Odds ratios are per SD unit increase in PRSs.

Training Data	Population	Comorbidity-adjusted		SUD-adjusted	
		OR (95% CI)	*p*-value	OR (95% CI)	*p*-value
PGC3-SCZ	EA	1.252 (1.175, 1.336)	7.20E-12	1.239 (1.161, 1.323)	1.20E-10
PGC3-BIP	EA	1.157 (1.085, 1.234)	9.20E-06	1.164 (1.09, 1.243)	5.30E-06
PGC-MDD(2018)	EA	0.984 (0.924, 1.048)	6.10E-01	0.972 (0.911, 1.036)	3.80E-01
gSEM-BIP	EA	1.024 (0.962, 1.09)	4.60E-01	1.041 (0.977, 1.109)	2.20E-01
gSEM-MDD	EA	1.089 (1.022, 1.16)	8.20E-03	1.086 (1.018, 1.158)	1.20E-02
gSEM-GEN	EA	0.865 (0.812, 0.922)	8.60E-06	0.86 (0.806, 0.918)	4.90E-06
gSEM-SCZ	EA	1.163 (1.091, 1.24)	3.40E-06	1.154 (1.082, 1.231)	1.40E-05
PGC3-SCZ	AA	1.148 (1.063, 1.241)	4.80E-04	1.172 (1.083, 1.268)	8.10E-05
PGC3-BIP	AA	1.07 (0.99, 1.156)	8.80E-02	1.066 (0.986, 1.154)	1.10E-01
PGC-MDD(2018)	AA	1.023 (0.937, 1.117)	6.20E-01	1.025 (0.937, 1.121)	5.90E-01
gSEM-BIP	AA	0.968 (0.907, 1.033)	3.30E-01	0.962 (0.9, 1.029)	2.60E-01
gSEM-MDD	AA	1.057 (0.98, 1.141)	1.50E-01	1.063 (0.984, 1.149)	1.20E-01
gSEM-GEN	AA	0.964 (0.898, 1.034)	3.00E-01	0.956 (0.89 , 1.028)	2.20E-01
gSEM-SCZ	AA	1.066 (0.99, 1.148)	9.30E-02	1.071 (0.993, 1.155)	7.50E-02

eTable 13. Associations of neuropsychiatric PRSs with number of hospitalizations for SCZ.

Among EA or AA participants with an inpatient ICD-9/10 code for SCZ, primary and gSEM-derived PRSs were tested for association with the number of discharge diagnoses (i.e. by negative binomial regression). Effect sizes and significance are displayed for two models: (i) adjusting for total number of comorbidities, sex, age, age^2, and ancestry PCs; (ii) adjusting for any lifetime diagnosis of SUD (phecode 316) or AUD (phecode 317) (separately), sex, age, age-squared, and ancestry PCs. Odds ratios are per SD unit increase in PRSs.

Training Data	Population	Comorbidity-adjusted			SUD-adjusted		
		β	SE	*p*-value	*β*	SE	*p*-value
PGC3-SCZ	EA	0.198	0.043	4.20E-06	0.202	0.051	6.50E-05
PGC3-BIP	EA	0.178	0.042	2.20E-05	0.184	0.05	2.10E-04
PGC-MDD(2018)	EA	0.005	0.043	9.10E-01	-0.004	0.052	9.40E-01
gSEM-BIP	EA	0.098	0.042	2.00E-02	0.104	0.05	3.70E-02
gSEM-MDD	EA	0.045	0.044	3.10E-01	0.035	0.052	5.00E-01
gSEM-GEN	EA	-0.206	0.04	3.10E-07	-0.216	0.047	4.90E-06
gSEM-SCZ	EA	0.144	0.043	8.80E-04	0.152	0.051	2.90E-03
PGC3-SCZ	AA	0.093	0.03	1.90E-03	0.088	0.031	5.50E-03
PGC3-BIP	AA	0.052	0.03	8.10E-02	0.043	0.031	1.60E-01
PGC-MDD(2018)	AA	0.029	0.034	4.00E-01	0.029	0.035	4.00E-01
gSEM-BIP	AA	-0.022	0.026	3.90E-01	-0.02	0.026	4.50E-01
gSEM-MDD	AA	0.075	0.029	1.10E-02	0.059	0.03	5.30E-02
gSEM-GEN	AA	-0.01	0.028	7.20E-01	-0.004	0.028	8.80E-01
gSEM-SCZ	AA	0.005	0.029	8.70E-01	0.005	0.03	8.50E-01

© 2022 Bigdeli TB et al. *JAMA Psychiatry*.

eTable 14. Associations of neuropsychiatric PRSs with inpatient treatment for BIP.

Among EA or AA participants with a diagnosis of BIP, primary and gSEM-derived PRSs were tested for association with ever having received inpatient treatment for BIP (i.e. by logistic regression). Effect sizes and significance are displayed for two models: (i) adjusting for total number of comorbidities, sex, age, age^2, and ancestry PCs; (ii) adjusting for any lifetime diagnosis of SUD (phecode 316) or AUD (phecode 317) (separately), sex, age, age-squared, and ancestry PCs. Odds ratios are per SD unit increase in PRSs.

Training Data	Population	Comorbidity-adjusted		SUD-adjusted	
		OR (95% CI)	*p*-value	OR (95% CI)	*p*-value
PGC3-SCZ	EA	1.131 (1.097, 1.166)	3.60E-15	1.118 (1.083, 1.153)	3.20E-12
PGC3-BIP	EA	1.149 (1.114, 1.186)	2.60E-18	1.14 (1.104, 1.177)	8.90E-16
PGC-MDD(2018)	EA	1.029 (0.998, 1.061)	6.40E-02	1.017 (0.986, 1.049)	2.90E-01
gSEM-BIP	EA	1.052 (1.02, 1.084)	1.10E-03	1.054 (1.022, 1.087)	8.40E-04
gSEM-MDD	EA	1.122 (1.088, 1.158)	1.90E-13	1.109 (1.075, 1.145)	9.70E-11
gSEM-GEN	EA	0.93 (0.902, 0.959)	3.10E-06	0.933 (0.905, 0.963)	1.50E-05
gSEM-SCZ	EA	1.031 (1.001, 1.063)	4.50E-02	1.027 (0.995, 1.059)	9.50E-02
PGC3-SCZ	AA	1.12 (1.043, 1.204)	2.00E-03	1.13 (1.049, 1.216)	1.20E-03
PGC3-BIP	AA	1.11 (1.034, 1.191)	4.00E-03	1.1 (1.023, 1.183)	1.00E-02
PGC-MDD(2018)	AA	1.026 (0.945, 1.114)	5.40E-01	1.029 (0.946, 1.119)	5.10E-01
gSEM-BIP	AA	1.063 (1.003, 1.126)	4.00E-02	1.065 (1.004, 1.131)	3.70E-02
gSEM-MDD	AA	1.089 (1.015, 1.168)	1.70E-02	1.075 (1.0, 1.154)	4.90E-02
gSEM-GEN	AA	0.922 (0.864, 0.983)	1.40E-02	0.915 (0.856, 0.977)	8.40E-03
gSEM-SCZ	AA	1.028 (0.96, 1.101)	4.30E-01	1.036 (0.965, 1.111)	3.30E-01

eTable 15. Associations of neuropsychiatric PRSs with number of hospitalizations for BIP.

Among EA or AA participants with an inpatient ICD-9/10 code for BIP, primary and gSEM-derived PRSs were tested for association with the number of discharge diagnoses (i.e. by negative binomial regression). Effect sizes and significance are displayed for two models: (i) adjusting for total number of comorbidities, sex, age, age^2, and ancestry PCs; (ii) adjusting for any lifetime diagnosis of SUD (phecode 316) or AUD (phecode 317) (separately), sex, age, age-squared, and ancestry PCs. Odds ratios are per SD unit increase in PRSs.

Training Data	Population	Comorbidity-adjusted			SUD-adjusted		
		β	SE	*p*-value	β	SE	*p*-value
PGC3-SCZ	EA	0.191	0.037	1.90E-07	0.188	0.036	2.40E-07
PGC3-BIP	EA	0.185	0.038	1.10E-06	0.168	0.038	1.00E-05
PGC-MDD	EA	-0.021	0.038	5.70E-01	-0.044	0.038	2.50E-01
gSEM-BIP	EA	0.044	0.038	2.40E-01	0.048	0.038	2.10E-01
gSEM-MDD	EA	0.135	0.037	2.70E-04	0.109	0.038	3.70E-03
gSEM-GEN	EA	-0.168	0.038	1.10E-05	-0.182	0.038	1.30E-06
gSEM-SCZ	EA	0.105	0.038	5.10E-03	0.119	0.038	1.50E-03
PGC3-SCZ	AA	0.276	0.074	1.80E-04	0.289	0.071	5.00E-05
PGC3-BIP	AA	0.225	0.074	2.20E-03	0.222	0.074	2.60E-03
PGC-MDD	AA	0.085	0.088	3.40E-01	0.103	0.087	2.30E-01
gSEM-BIP	AA	0.156	0.061	1.10E-02	0.168	0.06	4.90E-03
gSEM-MDD	AA	0.161	0.074	2.90E-02	0.152	0.073	3.80E-02
gSEM-GEN	AA	-0.184	0.068	6.70E-03	-0.192	0.066	3.90E-03
gSEM-SCZ	AA	0.111	0.073	1.30E-01	0.127	0.072	7.50E-02

© 2022 Bigdeli TB et al. *JAMA Psychiatry*.

eTable 16. Associations of neuropsychiatric PRSs with inpatient treatment for DEP.
Among EA or AA participants with a diagnosis of DEP, primary and gSEM-derived PRSs were tested for association with ever having received inpatient treatment for DEP (i.e. by logistic regression). Effect sizes and significance are displayed for two models: (i) adjusting for total number of comorbidities, sex, age, age^2, and ancestry PCs; (ii) adjusting for any lifetime diagnosis of SUD (phecode 316) or AUD (phecode 317) (separately), sex, age, age-squared, and ancestry PCs. Odds ratios are per SD unit increase in PRSs.

Training Data	Population	Comorbidity-adjusted		SUD-adjusted	
		OR (95% CI)	*p*-value	OR (95% CI)	*p*-value
PGC3-SCZ	EA	1.05 (1.036, 1.065)	2.20E-12	1.031 (1.017, 1.046)	1.60E-05
PGC3-BIP	EA	1.03 (1.016, 1.045)	3.00E-05	1.016 (1.002, 1.031)	3.00E-02
PGC-MDD (2018)	EA	1.043 (1.029, 1.057)	1.80E-09	1.036 (1.021, 1.05)	9.30E-07
gSEM-BIP	EA	0.979 (0.965, 0.992)	1.90E-03	0.979 (0.966, 0.993)	3.00E-03
gSEM-MDD	EA	1.049 (1.034, 1.063)	1.40E-11	1.034 (1.019, 1.048)	4.00E-06
gSEM-GEN	EA	1.0 (0.987, 1.014)	9.90E-01	1.007 (0.993, 1.021)	3.10E-01
gSEM-SCZ	EA	1.012 (0.998, 1.026)	9.20E-02	1.003 (0.989, 1.017)	6.50E-01
PGC3-SCZ	AA	1.042 (1.012, 1.072)	5.50E-03	1.032 (1.002, 1.063)	3.70E-02
PGC3-BIP	AA	1.015 (0.986, 1.044)	3.20E-01	1.009 (0.98, 1.039)	5.50E-01
PGC-MDD(2018)	AA	1.022 (0.99, 1.056)	1.80E-01	1.02 (0.986, 1.054)	2.50E-01
gSEM-BIP	AA	0.986 (0.963, 1.01)	2.40E-01	0.988 (0.964, 1.012)	3.20E-01
gSEM-MDD	AA	1.022 (0.993, 1.051)	1.30E-01	1.019 (0.989, 1.049)	2.10E-01
gSEM-GEN	AA	1.005 (0.979, 1.032)	7.00E-01	1.012 (0.986, 1.04)	3.70E-01
gSEM-SCZ	AA	1.016 (0.989, 1.044)	2.60E-01	1.009 (0.981, 1.038)	5.20E-01

eTable 17. Associations of neuropsychiatric PRSs with number of hospitalizations for DEP.
Among EA or AA participants with an inpatient ICD-9/10 code for DEP, primary and gSEM-derived PRSs were tested for association with the number of discharge diagnoses (i.e. by negative binomial regression). Effect sizes and significance are displayed for two models: (i) adjusting for total number of comorbidities, sex, age, age^2, and ancestry PCs; (ii) adjusting for any lifetime diagnosis of SUD (phecode 316) or AUD (phecode 317) (separately), sex, age, age-squared, and ancestry PCs. Odds ratios are per SD unit increase in PRSs.

Training Data	Population	Comorbidity-adjusted			SUD-adjusted		
		β	SE	*p*-value	*β*	SE	*p*-value
PGC3-SCZ	EA	0.066	0.015	1.90E-05	0.042	0.016	8.90E-03
PGC3-BIP	EA	0.049	0.016	1.70E-03	0.035	0.016	3.20E-02
PGC-MDD (2018)	EA	0.041	0.015	7.60E-03	0.034	0.016	3.30E-02
gSEM-BIP	EA	-0.024	0.015	1.10E-01	-0.021	0.016	1.90E-01
gSEM-MDD	EA	0.068	0.015	1.10E-05	0.06	0.016	2.50E-04
gSEM-GEN	EA	-0.01	0.015	5.10E-01	0.003	0.016	8.60E-01
gSEM-SCZ	EA	0.028	0.015	6.60E-02	0.014	0.016	4.00E-01
PGC3-SCZ	AA	0.032	0.033	3.30E-01	0.02	0.035	5.70E-01
PGC3-BIP	AA	0.013	0.033	6.90E-01	0.027	0.035	4.40E-01
PGC-MDD (2018)	AA	0.006	0.037	8.60E-01	0.009	0.039	8.30E-01
gSEM-BIP	AA	-0.028	0.027	2.90E-01	-0.021	0.028	4.60E-01
gSEM-MDD	AA	-0.006	0.033	8.60E-01	0.004	0.034	9.10E-01
gSEM-GEN	AA	-0.006	0.03	8.30E-01	0.004	0.031	9.10E-01
gSEM-SCZ	AA	0.035	0.031	2.70E-01	0.021	0.033	5.20E-01

© 2022 Bigdeli TB et al. *JAMA Psychiatry*.

eTable 18. Significant findings (p<10^{-25}) in PheWAS of SCZ PRS in EA participants.

Results are grouped by disease category, and numbers of controls and cases are displayed. Effect sizes and significance are reported for four models: (i) the primary analysis, adjusting for sex, age, age^2, and ancestry PCs; (ii) following exclusion individuals with SCZ-related diagnoses; (iii) adjusting for any SCZ-related diagnosis; or (iv) adjusting for lifetime treatment with antipsychotics. Odds ratios are per SD unit increase in PRS.

Group	Phecode	Description	Controls	Cases	Primary analysis		Exclude Phecode 295*		Phecode-adjusted		Antipsychotic-adjusted	
					OR (95% CI)	*p*-value	OR (95% CI)	*p*-value	OR (95% CI)	*p*-value	OR (95% CI)	*p*-value
circulatory system	418	Nonspecific chest pain	178521	91397	1.06 (1.05,1.07)	6.8E-50	1.05 (1.04,1.06)	2.0E-32	1.03 (1.03,1.04)	5.5E-16	1.04 (1.03,1.04)	3.0E-17
digestive	521	Diseases of hard tissues of teeth	233809	60055	1.09 (1.08,1.1)	6.5E-79	1.06 (1.05,1.08)	6.9E-36	1.06 (1.05,1.07)	1.0E-33	1.05 (1.04,1.06)	5.5E-26
digestive	521.1	Dental caries	236323	58432	1.09 (1.08,1.1)	3.3E-76	1.06 (1.05,1.07)	4.8E-34	1.06 (1.05,1.07)	2.8E-32	1.05 (1.04,1.06)	9.4E-25
digestive	523	Gingival and periodontal diseases	246246	49998	1.09 (1.08,1.1)	1.4E-61	1.06 (1.05,1.07)	5.7E-27	1.05 (1.04,1.07)	1.8E-25	1.05 (1.04,1.06)	8.8E-20
digestive	523.3	Periodontitis (acute or chronic)	271618	25654	1.08 (1.07,1.1)	4.2E-33	1.05 (1.04,1.07)	4.4E-13	1.05 (1.04,1.06)	5.3E-13	1.04 (1.03,1.06)	8.8E-10
digestive	525	Other diseases of the teeth and supporting structures	230287	60680	1.09 (1.08,1.1)	4.5E-75	1.06 (1.05,1.07)	3.2E-30	1.05 (1.04,1.06)	4.7E-29	1.05 (1.04,1.06)	9.2E-21
digestive	525.1	Loss of teeth or edentulism	261829	38073	1.07 (1.05,1.08)	4.2E-31	1.04 (1.03,1.05)	7.6E-10	1.03 (1.02,1.04)	5.1E-08	1.02 (1.01,1.03)	9.8E-05
digestive	578	Gastrointestinal hemorrhage	250313	35111	1.07 (1.06,1.08)	5.4E-31	1.06 (1.05,1.07)	2.5E-21	1.05 (1.04,1.06)	8.5E-16	1.04 (1.03,1.06)	1.2E-13
Endocrine /metabolic	276	Disorders of fluid, electrolyte, and acid-base balance	221908	54151	1.06 (1.05,1.07)	3.3E-30	1.03 (1.02,1.04)	5.1E-10	1.03 (1.02,1.04)	3.9E-10	1.02 (1.01,1.03)	6.7E-05
genitourinary	599	Other symptoms/disorders or the urinary system	215307	59968	1.05 (1.04,1.06)	2.4E-29	1.04 (1.03,1.05)	4.6E-18	1.03 (1.02,1.04)	8.2E-11	1.03 (1.02,1.04)	1.1E-11
infectious diseases	70.3	Viral hepatitis C	294978	13935	1.17 (1.15,1.19)	4.2E-70	1.15 (1.12,1.17)	4.8E-44	1.14 (1.12,1.16)	1.8E-50	1.13 (1.11,1.15)	1.6E-42
injuries & poisonings	969	Poisoning by psychotropic agents	306220	1944	1.3 (1.25,1.36)	1.2E-30	1.27 (1.19,1.36)	2.3E-13	1.25 (1.19,1.31)	6.5E-22	1.18 (1.12,1.23)	3.2E-12
mental disorders	290.3	Other persistent mental disorders due to conditions classified elsewhere	288225	14474	1.11 (1.09,1.13)	6.9E-32	1.07 (1.04,1.09)	2.9E-10	1.06 (1.04,1.08)	2.2E-11	1.05 (1.03,1.06)	3.1E-07
mental disorders	292	Neurological disorders	246745	40721	1.08 (1.06,1.09)	7.5E-42	1.05 (1.04,1.06)	1.0E-17	1.04 (1.03,1.05)	3.0E-11	1.03 (1.02,1.04)	1.7E-06
mental disorders	296	Mood disorders	161370	132442	1.12 (1.11,1.13)	1.3E-184	1.09 (1.08,1.1)	2.1E-102	0.87 (0.84,0.91)	2.8E-11	1.08 (1.07,1.08)	5.5E-66
mental disorders	296.1	Bipolar	285708	20399	1.29 (1.27,1.31)	1.0E-253	1.21 (1.19,1.23)	7.6E-93	1.24 (1.23,1.26)	5.5E-171	1.2 (1.18,1.22)	9.7E-99
mental disorders	296.2	Depression	167868	124718	1.1 (1.09,1.11)	1.4E-134	1.08 (1.07,1.09)	4.5E-83	0.9 (0.88,0.92)	2.5E-22	1.06 (1.05,1.07)	1.1E-42
mental disorders	296.22	Major depressive disorder	200712	92217	1.08 (1.08,1.09)	2.7E-84	1.07 (1.06,1.08)	1.4E-50	0.98 (0.97,1)	5.4E-03	1.04 (1.03,1.05)	7.6E-16
mental disorders	297	Suicidal ideation or attempt	285894	14832	1.19 (1.17,1.21)	9.4E-92	1.11 (1.09,1.14)	3.9E-23	1.14 (1.12,1.16)	8.8E-51	1.09 (1.07,1.11)	3.4E-22
mental disorders	297.1	Suicidal ideation	291719	12317	1.21 (1.19,1.23)	4.1E-90	1.13 (1.1,1.16)	1.6E-24	1.16 (1.14,1.18)	6.9E-52	1.11 (1.08,1.13)	5.6E-24
mental disorders	297.2	Suicide or self-inflicted injury	304207	3948	1.24 (1.2,1.28)	6.4E-40	1.17 (1.12,1.22)	6.0E-13	1.19 (1.15,1.23)	9.1E-26	1.12 (1.08,1.16)	8.4E-12
mental disorders	300	Anxiety disorders	166912	123000	1.13 (1.12,1.14)	8.6E-188	1.1 (1.09,1.11)	2.2E-116	1.07 (1.06,1.09)	1.4E-47	1.08 (1.07,1.09)	5.9E-76

© 2022 Bigdeli TB et al. *JAMA Psychiatry*.

mental disorders	300.1	Anxiety disorder	206217	79142	1.13 (1.12,1.14)	1.8E-176	1.11 (1.1,1.12)	3.8E-115	1.09 (1.08,1.1)	9.5E-65	1.09 (1.08,1.1)	5.4E-77
mental disorders	300.11	Generalized anxiety disorder	278052	21853	1.13 (1.12,1.15)	3.7E-67	1.12 (1.11,1.14)	1.6E-49	1.09 (1.07,1.1)	6.3E-30	1.08 (1.07,1.1)	3.0E-28
mental disorders	300.12	Agorophobia, social phobia, and panic disorder	292966	12188	1.14 (1.12,1.16)	6.8E-43	1.13 (1.1,1.15)	1.9E-29	1.09 (1.07,1.11)	4.9E-21	1.08 (1.06,1.1)	4.9E-15
mental disorders	300.3	Obsessive-compulsive disorders	306240	3142	1.24 (1.2,1.29)	3.1E-33	1.19 (1.14,1.24)	6.2E-16	1.19 (1.15,1.24)	4.7E-22	1.16 (1.12,1.21)	1.1E-16
mental disorders	300.4	Dysthymic disorder	274401	23556	1.08 (1.07,1.1)	3.7E-30	1.06 (1.04,1.08)	3.1E-14	1.02 (1.01,1.04)	5.4E-04	1.03 (1.02,1.04)	3.1E-05
mental disorders	300.9	Posttraumatic stress disorder	230809	69537	1.07 (1.07,1.08)	8.7E-57	1.06 (1.05,1.07)	3.2E-33	1.02 (1.01,1.03)	1.6E-06	1.03 (1.02,1.04)	9.0E-08
mental disorders	301	Personality disorders	290676	13909	1.21 (1.19,1.24)	1.3E-103	1.14 (1.12,1.17)	3.1E-33	1.17 (1.14,1.19)	1.8E-62	1.12 (1.1,1.14)	1.1E-32
mental disorders	301.2	Antisocial/borderline personality disorder	303440	5191	1.22 (1.19,1.25)	5.7E-43	1.16 (1.11,1.2)	2.4E-14	1.17 (1.14,1.21)	1.2E-27	1.11 (1.07,1.14)	1.8E-11
mental disorders	304	Adjustment reaction	238220	47151	1.06 (1.05,1.07)	6.8E-32	1.05 (1.04,1.06)	7.0E-20	1.02 (1.01,1.03)	2.6E-03	1.03 (1.02,1.04)	2.0E-09
mental disorders	306	Other mental disorder	257665	23904	1.11 (1.09,1.12)	3.2E-51	1.06 (1.05,1.08)	8.9E-15	1.07 (1.05,1.08)	5.8E-20	1.06 (1.04,1.07)	1.4E-15
mental disorders	316	Substance addiction and disorders	261155	36576	1.17 (1.15,1.18)	1.1E-148	1.12 (1.1,1.13)	8.6E-64	1.12 (1.11,1.14)	4.2E-81	1.1 (1.09,1.12)	5.8E-55
mental disorders	317.1	Alcoholism	255939	39754	1.15 (1.13,1.16)	2.4E-130	1.11 (1.1,1.13)	1.7E-71	1.11 (1.1,1.12)	7.7E-76	1.1 (1.09,1.11)	6.3E-61
mental disorders	318	Tobacco use disorder	175890	103781	1.07 (1.06,1.07)	1.9E-56	1.05 (1.04,1.05)	2.9E-26	1.04 (1.03,1.05)	3.1E-25	1.04 (1.03,1.05)	3.3E-21
respiratory	465	Acute upper respiratory infections of multiple or unspecified sites	204993	53253	1.09 (1.08,1.1)	8.5E-72	1.08 (1.07,1.09)	7.8E-46	1.07 (1.06,1.08)	9.4E-38	1.07 (1.05,1.08)	5.5E-36
sense organs	389	Hearing loss	121761	165684	0.94 (0.93,0.95)	9.6E-51	0.95 (0.94,0.95)	3.4E-41	0.93 (0.92,0.94)	4.2E-73	0.94 (0.93,0.94)	1.0E-62
sense organs	389.1	Sensorineural hearing loss	143787	132938	0.93 (0.93,0.94)	3.3E-60	0.94 (0.93,0.95)	7.0E-50	0.92 (0.91,0.93)	1.1E-83	0.93 (0.92,0.93)	1.1E-72
symptoms	785	Abdominal pain	206085	63494	1.05 (1.04,1.06)	6.0E-30	1.04 (1.03,1.05)	4.2E-19	1.03 (1.02,1.04)	1.5E-08	1.03 (1.02,1.04)	6.0E-08

eTable 19. Significant findings ($p<10^{-25}$) in PheWAS of BIP PRS in EA participants.

Results are grouped by disease category, and numbers of controls and cases are displayed. Effect sizes and significance are reported for four models: (i) the primary analysis, adjusting for sex, age, age^2, and ancestry PCs; (ii) following exclusion individuals with mood disorders; (iii) adjusting for any mood-disorder diagnosis; or (iv) adjusting for lifetime treatment with mood-stabilizers. Odds ratios are per SD unit increase in PRS.

Group	Phecode	Description	Controls	Case	Base		Exclude Phecode 296*		Phecode-adjusted		Mood stabilizer-adjusted	
					OR (95% CI)	*p*-value	OR (95% CI)	*p*-value	OR (95% CI)	*p*-value	OR (95% CI)	*p*-value
circulatory system	418	Nonspecific chest pain	178521	91397	1.01 (1.01,1.01)	2.90E-35	1.01 (1.01,1.01)	3.60E-24	1 (1,1.01)	8.30E-09	1.01 (1.01,1.01)	3.60E-19
digestive	521	Diseases of hard tissues of teeth	233809	60055	1.01 (1.01,1.02)	4.70E-56	1.01 (1.01,1.01)	6.10E-29	1.01 (1.01,1.01)	6.80E-20	1.01 (1.01,1.01)	6.20E-30
digestive	521.1	Dental caries	236323	58432	1.01 (1.01,1.02)	2.20E-55	1.01 (1.01,1.01)	2.30E-28	1.01 (1.01,1.01)	1.20E-19	1.01 (1.01,1.01)	2.30E-29
digestive	522	Diseases of pulp and periapical tissues	284618	14361	1.02 (1.02,1.02)	1.10E-28	1.01 (1.01,1.02)	1.50E-15	1.01 (1.01,1.02)	3.70E-14	1.01 (1.01,1.02)	8.50E-17

© 2022 Bigdeli TB et al. *JAMA Psychiatry*.

digestive	523	Gingival and periodontal diseases	246246	49998	1.01 (1.01,1.02)	1.00E-45	1.01 (1.01,1.01)	1.70E-23	1.01 (1.01,1.01)	5.00E-16	1.01 (1.01,1.01)	3.10E-23
digestive	523.3	Periodontitis (acute or chronic)	271618	25654	1.02 (1.01,1.02)	2.40E-33	1.01 (1.01,1.01)	5.70E-17	1.01 (1.01,1.01)	1.70E-13	1.01 (1.01,1.01)	8.30E-19
digestive	525	Other diseases of the teeth and supporting structures	230287	60680	1.01 (1.01,1.02)	8.10E-53	1.01 (1.01,1.01)	6.00E-25	1.01 (1.01,1.01)	2.20E-16	1.01 (1.01,1.01)	5.10E-26
digestive	525.1	Loss of teeth or edentulism	261829	38073	1.01 (1.01,1.02)	3.10E-35	1.01 (1.01,1.01)	4.20E-18	1.01 (1,1.01)	1.20E-10	1.01 (1.01,1.01)	6.00E-17
genitourinary	599	Other symptoms/disorders or the urinary system	215307	59968	1.01 (1.01,1.01)	2.90E-26	1.01 (1.01,1.01)	7.00E-17	1.01 (1,1.01)	7.20E-09	1.01 (1.01,1.01)	2.20E-15
infectious diseases	70.3	Viral hepatitis C	294978	13935	1.02 (1.02,1.02)	1.20E-32	1.02 (1.02,1.02)	4.60E-27	1.02 (1.01,1.02)	4.20E-20	1.02 (1.01,1.02)	4.10E-26
mental disorders	290.3	Other persistent mental disorders due to conditions classified elsewhere	288225	14474	1.02 (1.01,1.02)	6.50E-26	1.01 (1.01,1.02)	8.20E-11	1.01 (1.01,1.01)	1.70E-08	1.01 (1.01,1.02)	1.20E-12
mental disorders	292	Neurological disorders	246745	40721	1.01 (1.01,1.02)	1.80E-40	1.01 (1.01,1.01)	2.40E-20	1.01 (1,1.01)	1.10E-11	1.01 (1.01,1.01)	2.20E-19
mental disorders	296	Mood disorders	161370	132442	1.02 (1.02,1.02)	7.00E-193	1.02 (1.02,1.02)	1.20E-124	1 (0.99,1.01)	0.9	1.02 (1.02,1.02)	1.40E-121
mental disorders	296.1	Bipolar	285708	20399	1.05 (1.05,1.06)	7.10E-255	1.04 (1.04,1.05)	2.20E-114	1.04 (1.04,1.05)	1.20E-171	1.05 (1.04,1.05)	3.60E-154
mental disorders	296.2	Depression	167868	124718	1.02 (1.02,1.02)	1.10E-138	1.02 (1.02,1.02)	3.70E-96	0.99 (0.98,0.99)	5.90E-11	1.02 (1.01,1.02)	7.30E-86
mental disorders	296.22	Major depressive disorder	200712	92217	1.02 (1.02,1.02)	4.50E-97	1.02 (1.01,1.02)	3.00E-67	1 (1,1)	0.98	1.01 (1.01,1.01)	9.60E-54
mental disorders	297	Suicidal ideation or attempt	285894	14832	1.03 (1.03,1.04)	6.50E-85	1.02 (1.02,1.03)	3.80E-31	1.03 (1.02,1.03)	3.50E-46	1.03 (1.02,1.03)	2.20E-47
mental disorders	297.1	Suicidal ideation	291719	12317	1.04 (1.03,1.04)	9.00E-80	1.03 (1.02,1.03)	3.00E-30	1.03 (1.02,1.03)	3.00E-44	1.03 (1.02,1.03)	5.20E-45
mental disorders	300	Anxiety disorders	166912	123000	1.02 (1.02,1.02)	1.40E-137	1.02 (1.01,1.02)	3.10E-89	1.01 (1.01,1.01)	4.60E-22	1.02 (1.01,1.02)	5.90E-83
mental disorders	300.1	Anxiety disorder	206217	79142	1.02 (1.02,1.02)	1.20E-132	1.02 (1.02,1.02)	3.70E-88	1.01 (1.01,1.01)	2.20E-39	1.02 (1.02,1.02)	4.30E-84
mental disorders	300.11	Generalized anxiety disorder	278052	21853	1.02 (1.02,1.02)	2.20E-37	1.02 (1.01,1.02)	8.40E-28	1.01 (1.01,1.01)	1.80E-11	1.01 (1.01,1.02)	3.70E-22
mental disorders	300.4	Dysthymic disorder	274401	23556	1.02 (1.01,1.02)	1.70E-30	1.01 (1.01,1.02)	1.60E-17	1 (1,1.01)	0.00027	1.01 (1.01,1.01)	5.80E-15
mental disorders	300.9	Posttraumatic stress disorder	230809	69537	1.01 (1.01,1.01)	2.50E-47	1.01 (1.01,1.01)	1.70E-26	1 (1,1)	0.001	1.01 (1.01,1.01)	1.20E-19
mental disorders	301	Personality disorders	290676	13909	1.04 (1.03,1.04)	8.90E-89	1.03 (1.02,1.03)	8.60E-37	1.03 (1.02,1.03)	2.30E-50	1.03 (1.02,1.03)	2.50E-50
mental disorders	301.2	Antisocial/borderline personality disorder	303440	5191	1.04 (1.03,1.04)	1.20E-38	1.03 (1.02,1.04)	4.00E-16	1.03 (1.02,1.04)	1.20E-23	1.03 (1.02,1.03)	2.90E-20
mental disorders	304	Adjustment reaction	238220	47151	1.01 (1.01,1.02)	2.60E-39	1.01 (1.01,1.01)	6.90E-28	1 (1,1.01)	7.50E-06	1.01 (1.01,1.01)	9.60E-24
mental disorders	306	Other mental disorder	257665	23904	1.02 (1.02,1.02)	8.00E-41	1.01 (1.01,1.02)	4.30E-17	1.01 (1.01,1.01)	5.60E-14	1.01 (1.01,1.02)	2.70E-20
mental disorders	316	Substance addiction and disorders	261155	36576	1.03 (1.03,1.03)	6.00E-121	1.02 (1.02,1.02)	8.40E-64	1.02 (1.02,1.02)	6.30E-62	1.02 (1.02,1.03)	3.20E-78
mental disorders	317.1	Alcoholism	255939	39754	1.02 (1.02,1.02)	3.40E-64	1.01 (1.01,1.02)	4.00E-34	1.01 (1.01,1.01)	1.30E-28	1.02 (1.01,1.02)	5.20E-42
mental disorders	318	Tobacco use disorder	175890	103781	1.01 (1.01,1.01)	4.90E-34	1.01 (1.01,1.01)	2.00E-17	1.01 (1,1.01)	9.60E-12	1.01 (1.01,1.01)	2.20E-21
neurological	327.4	Insomnia	230277	51649	1.01 (1.01,1.01)	1.40E-26	1.01 (1.01,1.01)	9.70E-18	1 (1,1.01)	0.00082	1.01 (1,1.01)	5.90E-12

© 2022 Bigdeli TB et al. *JAMA Psychiatry*.

respiratory	465	Acute upper respiratory infections of multiple or unspecified sites	204993	53253	1.01 (1.01,1.02)	3.50E-49	1.01 (1.01,1.01)	1.60E-31	1.01 (1.01,1.01)	6.10E-22	1.01 (1.01,1.01)	6.00E-30

eTable 20. Significant findings ($p<10^{-25}$) in PheWAS of DEP PRS in EA participants.

Results are grouped by disease category, and numbers of controls and cases are displayed. Effect sizes and significance are reported for four models: (i) the primary analysis, adjusting for sex, age, age^2, and ancestry PCs; (ii) following exclusion individuals with mood disorders; (iii) adjusting for any mood-disorder diagnosis; or (iv) adjusting for lifetime treatment with antidepressants. Odds ratios are per SD unit increase in PRS.

Group	Phecode	Description	Controls	Case	Base		Exclude Phecode 296*		Phecode-adjusted		Antidepressant-adjusted	
					OR (95% CI)	*p*-value	OR (95% CI)	*p*-value	OR (95% CI)	*p*-value	OR (95% CI)	*p*-value
circulatory system	401	Hypertension	69818	225389	1.06 (1.05,1.07)	7.30E-40	1.06 (1.05,1.07)	6.20E-33	1.04 (1.03,1.05)	2.50E-19	1.04 (1.03,1.05)	5.90E-17
circulatory system	401.1	Essential hypertension	70815	224179	1.06 (1.05,1.07)	2.40E-40	1.06 (1.05,1.07)	2.70E-33	1.04 (1.03,1.05)	8.10E-20	1.04 (1.03,1.05)	2.30E-17
circulatory system	411	Ischemic Heart Disease	187667	100858	1.08 (1.07,1.09)	4.20E-71	1.07 (1.07,1.08)	8.10E-63	1.06 (1.05,1.07)	2.20E-42	1.06 (1.05,1.07)	3.10E-39
circulatory system	411.1	Unstable angina (intermediate coronary syndrome)	287650	11889	1.12 (1.1,1.14)	1.80E-33	1.12 (1.1,1.15)	1.40E-30	1.09 (1.07,1.11)	1.60E-20	1.09 (1.07,1.11)	1.90E-19
circulatory system	411.2	Myocardial infarction	270506	27694	1.08 (1.06,1.09)	1.50E-29	1.07 (1.06,1.09)	2.80E-26	1.06 (1.04,1.07)	2.50E-17	1.05 (1.04,1.07)	1.40E-16
circulatory system	411.3	Angina pectoris	265965	26054	1.08 (1.07,1.1)	2.50E-34	1.08 (1.06,1.09)	3.60E-28	1.06 (1.05,1.07)	3.30E-18	1.06 (1.04,1.07)	2.30E-17
circulatory system	411.4	Coronary atherosclerosis	205059	88022	1.07 (1.06,1.08)	6.10E-57	1.07 (1.06,1.08)	3.20E-51	1.05 (1.05,1.06)	1.10E-34	1.05 (1.04,1.06)	5.10E-32
circulatory system	411.8	Other chronic ischemic heart disease, unspecified	242159	53680	1.07 (1.06,1.08)	8.70E-40	1.07 (1.06,1.08)	2.90E-35	1.05 (1.04,1.06)	2.50E-24	1.05 (1.04,1.06)	4.60E-22
circulatory system	418	Nonspecific chest pain	178521	91397	1.1 (1.09,1.11)	7.90E-125	1.09 (1.09,1.1)	3.50E-96	1.07 (1.06,1.08)	1.80E-59	1.07 (1.06,1.08)	3.40E-56
circulatory system	427	Cardiac dysrhythmias	168977	104053	1.05 (1.04,1.06)	5.60E-37	1.05 (1.04,1.06)	3.80E-30	1.03 (1.02,1.04)	2.20E-15	1.03 (1.02,1.04)	3.20E-14
circulatory system	428	Congestive heart failure; nonhypertensive	254758	41526	1.08 (1.07,1.09)	7.90E-42	1.08 (1.06,1.09)	9.10E-38	1.05 (1.04,1.07)	6.80E-22	1.05 (1.04,1.06)	5.40E-21
circulatory system	433	Cerebrovascular disease	239598	49889	1.06 (1.05,1.07)	5.80E-29	1.05 (1.04,1.07)	6.00E-23	1.03 (1.02,1.04)	5.30E-11	1.03 (1.02,1.04)	7.00E-11
circulatory system	458	Hypotension	256763	27983	1.08 (1.07,1.1)	6.60E-37	1.08 (1.06,1.09)	5.00E-26	1.05 (1.03,1.06)	2.40E-12	1.05 (1.03,1.06)	4.40E-12
circulatory system	458.9	Hypotension NOS	273868	16517	1.09 (1.08,1.11)	1.30E-27	1.08 (1.07,1.1)	6.00E-20	1.06 (1.04,1.07)	2.10E-11	1.06 (1.04,1.07)	3.20E-11
dermatologic	681	Superficial cellulitis and abscess	224657	52705	1.06 (1.05,1.07)	5.60E-30	1.05 (1.04,1.06)	2.00E-20	1.03 (1.02,1.04)	3.70E-09	1.03 (1.02,1.04)	1.00E-08
digestive	521	Diseases of hard tissues of teeth	233809	60055	1.07 (1.06,1.08)	1.10E-46	1.06 (1.05,1.07)	8.40E-34	1.03 (1.02,1.04)	1.30E-10	1.03 (1.02,1.04)	7.90E-11
digestive	521.1	Dental caries	236323	58432	1.07 (1.06,1.08)	4.40E-46	1.06 (1.05,1.07)	1.80E-33	1.03 (1.02,1.04)	1.20E-10	1.03 (1.02,1.04)	7.00E-11
digestive	523	Gingival and periodontal diseases	246246	49998	1.07 (1.06,1.08)	1.60E-38	1.06 (1.05,1.07)	4.50E-28	1.03 (1.02,1.04)	1.40E-08	1.03 (1.02,1.04)	1.50E-08
digestive	525	Other diseases of the teeth and supporting structures	230287	60680	1.08 (1.07,1.09)	2.90E-67	1.08 (1.07,1.09)	2.60E-49	1.04 (1.03,1.05)	3.30E-19	1.04 (1.03,1.05)	8.30E-20
digestive	525.1	Loss of teeth or edentulism	261829	38073	1.08 (1.07,1.09)	7.50E-43	1.07 (1.06,1.08)	1.20E-29	1.04 (1.03,1.05)	5.60E-11	1.04 (1.03,1.05)	4.40E-11

© 2022 Bigdeli TB et al. *JAMA Psychiatry*.

digestive	530	Diseases of esophagus	153615	131627	1.08 (1.07,1.09)	2.30E-84	1.07 (1.06,1.08)	2.50E-70	1.05 (1.04,1.06)	7.00E-38	1.05 (1.04,1.06)	5.30E-34
digestive	530.1	Esophagitis, GERD and related diseases	156612	128556	1.08 (1.07,1.09)	2.70E-84	1.07 (1.06,1.08)	1.90E-70	1.05 (1.04,1.06)	5.40E-38	1.05 (1.04,1.06)	4.30E-34
digestive	530.11	GERD	162469	122039	1.08 (1.07,1.09)	3.30E-84	1.07 (1.07,1.08)	1.30E-70	1.05 (1.04,1.06)	2.00E-38	1.05 (1.04,1.06)	2.00E-34
digestive	532	Dysphagia	252740	40103	1.07 (1.05,1.08)	3.70E-31	1.06 (1.05,1.07)	2.40E-24	1.03 (1.02,1.05)	4.20E-10	1.03 (1.02,1.04)	2.50E-09
digestive	561	Symptoms involving digestive system	233209	41830	1.08 (1.07,1.09)	5.20E-46	1.08 (1.06,1.09)	2.40E-36	1.05 (1.03,1.06)	9.00E-16	1.04 (1.03,1.06)	1.40E-15
digestive	578	Gastrointestinal hemorrhage	250313	35111	1.07 (1.05,1.08)	2.20E-28	1.06 (1.05,1.07)	1.40E-22	1.04 (1.03,1.05)	3.40E-12	1.04 (1.03,1.05)	1.70E-11
endocrine/metabolic	250	Diabetes mellitus	189084	108545	1.05 (1.04,1.05)	1.80E-30	1.04 (1.04,1.05)	5.60E-26	1.03 (1.02,1.04)	1.90E-15	1.03 (1.02,1.04)	2.80E-13
endocrine/metabolic	250.2	Type 2 diabetes	190540	107822	1.04 (1.04,1.05)	4.80E-29	1.04 (1.03,1.05)	6.10E-25	1.03 (1.02,1.04)	1.50E-14	1.03 (1.02,1.04)	1.90E-12
endocrine/metabolic	250.24	Type 2 diabetes with neurological manifestations	259077	40530	1.06 (1.05,1.08)	1.10E-30	1.07 (1.05,1.08)	1.10E-28	1.04 (1.03,1.05)	1.60E-13	1.04 (1.02,1.05)	2.20E-10
endocrine/metabolic	276	Disorders of fluid, electrolyte, and acid-base balance	221908	54151	1.07 (1.06,1.08)	2.10E-43	1.06 (1.05,1.07)	8.60E-30	1.04 (1.03,1.05)	3.40E-15	1.04 (1.03,1.05)	1.20E-14
endocrine/metabolic	276.1	Electrolyte imbalance	242364	39794	1.06 (1.05,1.07)	5.90E-26	1.05 (1.04,1.06)	1.30E-16	1.03 (1.02,1.04)	2.90E-09	1.03 (1.02,1.04)	4.40E-09
genitourinary	585	Renal failure	230047	61932	1.05 (1.04,1.06)	7.30E-26	1.05 (1.04,1.06)	8.50E-21	1.03 (1.02,1.04)	1.30E-10	1.03 (1.02,1.04)	1.70E-09
genitourinary	585.1	Acute renal failure	263505	29679	1.07 (1.06,1.08)	3.80E-28	1.06 (1.05,1.08)	7.20E-21	1.04 (1.03,1.06)	4.40E-11	1.04 (1.03,1.05)	1.50E-10
genitourinary	591	Urinary tract infection	252052	33029	1.07 (1.06,1.08)	6.30E-30	1.07 (1.05,1.08)	8.30E-23	1.04 (1.03,1.06)	6.20E-12	1.04 (1.03,1.06)	8.80E-12
genitourinary	599	Other symptoms/disorders or the urinary system	215307	59968	1.05 (1.04,1.06)	4.80E-29	1.05 (1.04,1.06)	2.50E-21	1.03 (1.02,1.04)	7.70E-08	1.03 (1.02,1.04)	8.40E-08
genitourinary	600	Hyperplasia of prostate	189050	97473	1.05 (1.04,1.06)	5.40E-27	1.04 (1.03,1.05)	2.40E-21	1.03 (1.02,1.04)	1.90E-10	1.03 (1.02,1.04)	4.40E-10
hematopoietic	285	Other anemias	223129	59404	1.05 (1.04,1.06)	1.00E-26	1.05 (1.04,1.06)	1.30E-21	1.03 (1.02,1.04)	7.80E-10	1.03 (1.02,1.04)	4.30E-09
mental disorders	292	Neurological disorders	246745	40721	1.07 (1.06,1.08)	6.40E-33	1.05 (1.04,1.06)	7.20E-17	1.02 (1.01,1.03)	0.00023	1.03 (1.01,1.04)	3.80E-06
mental disorders	296	Mood disorders	161370	132442	1.15 (1.14,1.16)	7.60E-271	1.14 (1.13,1.15)	2.60E-219	1.08 (1.03,1.12)	0.00028	1.1 (1.09,1.11)	5.40E-68
mental disorders	296.1	Bipolar	285708	20399	1.16 (1.15,1.18)	8.20E-91	1.16 (1.14,1.18)	1.60E-55	1.1 (1.08,1.12)	1.90E-35	1.12 (1.1,1.14)	2.90E-49
mental disorders	296.2	Depression	167868	124718	1.15 (1.14,1.16)	3.20E-257	1.14 (1.13,1.15)	9.80E-210	1.05 (1.03,1.07)	1.90E-05	1.09 (1.08,1.1)	1.20E-59
mental disorders	296.22	Major depressive disorder	200712	92217	1.14 (1.13,1.15)	1.10E-205	1.13 (1.12,1.14)	2.20E-165	1.05 (1.04,1.07)	6.00E-18	1.08 (1.07,1.09)	1.60E-47
mental disorders	297	Suicidal ideation or attempt	285894	14832	1.16 (1.14,1.18)	1.90E-64	1.13 (1.11,1.16)	5.80E-31	1.1 (1.08,1.12)	1.50E-26	1.11 (1.09,1.13)	1.30E-30
mental disorders	297.1	Suicidal ideation	291719	12317	1.16 (1.14,1.18)	1.50E-53	1.13 (1.1,1.16)	2.70E-25	1.1 (1.08,1.12)	1.60E-21	1.1 (1.08,1.13)	6.00E-25
mental disorders	300	Anxiety disorders	166912	123000	1.13 (1.12,1.14)	2.00E-193	1.12 (1.11,1.13)	1.10E-151	1.06 (1.05,1.07)	8.50E-33	1.07 (1.06,1.08)	2.60E-41
mental disorders	300.1	Anxiety disorder	206217	79142	1.12 (1.11,1.13)	6.40E-137	1.11 (1.1,1.12)	4.50E-106	1.06 (1.05,1.07)	2.10E-29	1.06 (1.05,1.07)	2.50E-33
mental disorders	300.11	Generalized anxiety disorder	278052	21853	1.1 (1.08,1.11)	8.00E-37	1.1 (1.08,1.12)	4.00E-33	1.04 (1.03,1.06)	4.20E-08	1.04 (1.03,1.06)	8.50E-09

© 2022 Bigdeli TB et al. *JAMA Psychiatry*.

mental disorders	300.12	Agorophobia, social phobia, and panic disorder	292966	12188	1.12 (1.1,1.14)	7.20E-33	1.13 (1.1,1.15)	2.20E-30	1.07 (1.05,1.09)	4.20E-12	1.07 (1.05,1.09)	4.10E-12
mental disorders	300.4	Dysthymic disorder	274401	23556	1.12 (1.11,1.14)	1.30E-63	1.11 (1.1,1.13)	3.00E-45	1.06 (1.04,1.07)	1.20E-14	1.07 (1.05,1.08)	5.00E-19
mental disorders	300.9	Posttraumatic stress disorder	230809	69537	1.1 (1.09,1.11)	6.20E-99	1.09 (1.08,1.1)	1.30E-74	1.04 (1.03,1.05)	8.70E-18	1.05 (1.04,1.06)	2.70E-22
mental disorders	301	Personality disorders	290676	13909	1.18 (1.16,1.2)	2.30E-75	1.17 (1.14,1.19)	2.60E-43	1.12 (1.1,1.14)	3.40E-35	1.13 (1.11,1.15)	1.60E-40
mental disorders	301.2	Antisocial/borderline personality disorder	303440	5191	1.2 (1.16,1.23)	4.10E-35	1.19 (1.14,1.23)	2.10E-19	1.14 (1.11,1.17)	4.70E-19	1.15 (1.11,1.18)	6.10E-21
mental disorders	304	Adjustment reaction	238220	47151	1.08 (1.07,1.09)	4.50E-46	1.07 (1.06,1.08)	5.50E-37	1.02 (1.01,1.03)	2.30E-05	1.03 (1.02,1.04)	5.80E-09
mental disorders	306	Other mental disorder	257665	23904	1.1 (1.08,1.11)	4.50E-41	1.08 (1.07,1.1)	2.00E-25	1.05 (1.03,1.06)	6.70E-11	1.05 (1.04,1.07)	3.20E-13
mental disorders	316	Substance addiction and disorders	261155	36576	1.12 (1.11,1.14)	2.80E-87	1.11 (1.1,1.13)	3.00E-58	1.07 (1.06,1.09)	3.80E-31	1.08 (1.07,1.09)	2.50E-36
mental disorders	317.1	Alcoholism	255939	39754	1.09 (1.08,1.1)	3.50E-52	1.08 (1.06,1.09)	1.50E-33	1.05 (1.04,1.06)	1.20E-16	1.05 (1.04,1.07)	1.60E-20
mental disorders	318	Tobacco use disorder	175890	103781	1.09 (1.08,1.1)	4.80E-97	1.08 (1.07,1.09)	3.80E-77	1.06 (1.05,1.07)	1.20E-49	1.06 (1.05,1.07)	2.10E-50
musculoskeletal	716	Other arthropathies	247336	33669	1.06 (1.05,1.08)	3.50E-26	1.06 (1.05,1.08)	9.60E-24	1.04 (1.03,1.05)	1.50E-11	1.04 (1.02,1.05)	1.10E-09
musculoskeletal	721	Spondylosis and allied disorders	238955	44356	1.07 (1.06,1.08)	4.90E-37	1.07 (1.05,1.08)	1.20E-31	1.04 (1.03,1.05)	1.40E-13	1.03 (1.02,1.04)	4.00E-10
musculoskeletal	721.1	Spondylosis without myelopathy	242860	40805	1.07 (1.06,1.08)	7.50E-34	1.06 (1.05,1.08)	2.60E-28	1.04 (1.03,1.05)	2.30E-12	1.03 (1.02,1.04)	3.00E-09
musculoskeletal	722	Intervertebral disc disorders	228596	55337	1.07 (1.06,1.08)	2.50E-44	1.07 (1.06,1.08)	8.80E-38	1.04 (1.03,1.05)	3.40E-17	1.04 (1.03,1.05)	1.20E-12
musculoskeletal	722.6	Degeneration of intervertebral disc	242906	42226	1.06 (1.05,1.07)	8.60E-31	1.06 (1.05,1.07)	1.20E-25	1.04 (1.03,1.05)	2.30E-11	1.03 (1.02,1.04)	3.10E-08
musculoskeletal	740	Osteoarthrosis	146222	131442	1.06 (1.05,1.06)	4.10E-46	1.05 (1.04,1.06)	8.80E-38	1.03 (1.03,1.04)	1.10E-17	1.03 (1.02,1.04)	3.40E-14
musculoskeletal	740.9	Osteoarthrosis NOS	179236	99593	1.07 (1.06,1.07)	8.80E-56	1.06 (1.05,1.07)	1.70E-44	1.04 (1.03,1.05)	5.80E-24	1.04 (1.03,1.05)	3.30E-20
musculoskeletal	745	Pain in joint	89094	187207	1.07 (1.06,1.07)	2.20E-52	1.06 (1.05,1.07)	2.30E-44	1.04 (1.03,1.04)	7.10E-17	1.03 (1.02,1.04)	2.80E-14
neurological	327	Sleep disorders	191386	81129	1.08 (1.07,1.09)	9.40E-70	1.07 (1.07,1.08)	1.30E-57	1.03 (1.03,1.04)	7.20E-14	1.03 (1.02,1.04)	1.50E-12
neurological	327.3	Sleep apnea	195189	101623	1.04 (1.04,1.05)	6.00E-28	1.04 (1.03,1.05)	7.70E-25	1.02 (1.01,1.03)	2.20E-05	1.02 (1.01,1.02)	3.50E-05
neurological	327.4	Insomnia	230277	51649	1.07 (1.06,1.08)	1.40E-44	1.07 (1.06,1.08)	1.10E-36	1.03 (1.02,1.04)	4.20E-08	1.03 (1.02,1.04)	1.40E-07
neurological	338	Pain	225658	47039	1.09 (1.08,1.1)	4.60E-66	1.09 (1.08,1.1)	7.30E-52	1.05 (1.04,1.07)	6.50E-24	1.05 (1.04,1.06)	4.20E-21
neurological	338.2	Chronic pain	248178	34632	1.1 (1.09,1.11)	1.80E-57	1.09 (1.08,1.1)	6.10E-43	1.06 (1.05,1.07)	4.70E-21	1.05 (1.04,1.07)	1.70E-18
neurological	339	Other headache syndromes	237942	42426	1.09 (1.08,1.1)	1.10E-57	1.09 (1.07,1.1)	1.20E-45	1.05 (1.04,1.07)	2.60E-21	1.05 (1.04,1.06)	5.30E-18
neurological	350	Abnormal movement	220347	57430	1.07 (1.06,1.08)	5.00E-43	1.06 (1.05,1.07)	3.50E-29	1.03 (1.02,1.04)	2.10E-09	1.03 (1.02,1.04)	1.30E-09
neurological	350.2	Abnormality of gait	238406	44292	1.07 (1.06,1.08)	5.70E-37	1.06 (1.05,1.07)	1.40E-25	1.03 (1.02,1.04)	3.80E-09	1.03 (1.02,1.04)	2.80E-09
neurological	351	Other peripheral nerve disorders	227740	57720	1.05 (1.05,1.06)	7.50E-30	1.05 (1.04,1.06)	3.60E-25	1.03 (1.02,1.04)	6.50E-09	1.02 (1.01,1.03)	1.60E-05

© 2022 Bigdeli TB et al. *JAMA Psychiatry*.

neurological	355.1	Chronic pain syndrome	283361	17187	1.11 (1.09,1.12)	7.50E-37	1.1 (1.09,1.12)	1.30E-30	1.06 (1.05,1.08)	1.30E-14	1.06 (1.04,1.08)	1.30E-12
respiratory	465	Acute upper respiratory infections of multiple or unspecified sites	204993	53253	1.07 (1.06,1.08)	5.10E-41	1.06 (1.05,1.07)	4.80E-30	1.04 (1.03,1.05)	3.90E-14	1.04 (1.03,1.05)	1.50E-13
respiratory	480	Pneumonia	260383	31337	1.08 (1.07,1.09)	2.50E-35	1.07 (1.05,1.08)	1.00E-23	1.05 (1.04,1.06)	1.00E-14	1.05 (1.03,1.06)	5.80E-14
respiratory	496	Chronic airway obstruction	215720	72353	1.1 (1.09,1.11)	2.00E-108	1.1 (1.09,1.11)	5.50E-91	1.08 (1.07,1.09)	1.80E-61	1.08 (1.07,1.09)	1.20E-59
respiratory	496.2	Chronic bronchitis	276924	19677	1.12 (1.1,1.13)	8.00E-49	1.11 (1.09,1.13)	2.00E-39	1.09 (1.07,1.1)	2.70E-27	1.08 (1.07,1.1)	5.70E-27
respiratory	496.21	Obstructive chronic bronchitis	282188	17154	1.12 (1.1,1.13)	2.20E-42	1.11 (1.09,1.13)	6.80E-35	1.08 (1.07,1.1)	2.30E-23	1.08 (1.07,1.1)	4.60E-23
respiratory	497	Bronchitis	268282	14750	1.09 (1.08,1.11)	3.90E-26	1.09 (1.07,1.11)	1.50E-21	1.06 (1.04,1.08)	3.90E-12	1.06 (1.04,1.08)	3.60E-12
respiratory	509	Respiratory failure, insufficiency, arrest	275454	22821	1.09 (1.07,1.1)	5.50E-32	1.08 (1.07,1.1)	1.40E-27	1.06 (1.04,1.07)	1.20E-15	1.06 (1.04,1.07)	1.20E-14
respiratory	509.1	Respiratory failure	284540	17095	1.09 (1.08,1.11)	1.60E-28	1.09 (1.07,1.11)	1.60E-24	1.06 (1.05,1.08)	6.20E-15	1.06 (1.05,1.08)	4.30E-14
respiratory	512	Other symptoms of respiratory system	139662	120187	1.08 (1.07,1.09)	3.60E-85	1.08 (1.07,1.08)	5.30E-69	1.05 (1.04,1.06)	5.20E-31	1.05 (1.04,1.06)	9.60E-29
respiratory	512.7	Shortness of breath	209689	60907	1.08 (1.07,1.09)	6.30E-60	1.07 (1.06,1.08)	3.90E-48	1.05 (1.04,1.06)	1.70E-24	1.05 (1.04,1.06)	1.50E-22
respiratory	512.8	Cough	212486	48953	1.06 (1.05,1.07)	2.70E-27	1.05 (1.04,1.06)	2.80E-19	1.03 (1.02,1.04)	1.80E-08	1.03 (1.02,1.04)	4.20E-08
respiratory	512.9	Other dyspnea	219490	48646	1.07 (1.06,1.08)	1.50E-37	1.06 (1.05,1.07)	3.50E-30	1.04 (1.03,1.05)	6.40E-14	1.04 (1.03,1.05)	6.50E-13
sense organs	366	Cataract	116658	166416	1.05 (1.05,1.06)	1.70E-37	1.05 (1.04,1.06)	9.50E-32	1.03 (1.02,1.04)	1.10E-13	1.03 (1.02,1.04)	3.00E-12
sense organs	366.2	Senile cataract	139596	142445	1.04 (1.04,1.05)	1.20E-27	1.04 (1.03,1.05)	4.10E-23	1.02 (1.02,1.03)	1.70E-09	1.02 (1.01,1.03)	1.40E-08
sense organs	367	Disorders of refraction and accommodation; blindness and low vision	86906	200705	1.05 (1.04,1.06)	1.60E-35	1.05 (1.04,1.06)	4.20E-27	1.03 (1.02,1.03)	1.40E-09	1.02 (1.02,1.03)	1.70E-08
sense organs	386.9	Dizziness and giddiness (Light-headedness and vertigo)	231860	43001	1.08 (1.07,1.09)	3.00E-44	1.07 (1.06,1.08)	3.80E-32	1.04 (1.03,1.06)	1.30E-15	1.04 (1.03,1.06)	1.20E-15
symptoms	760	Back pain	128684	149486	1.09 (1.08,1.1)	1.50E-105	1.08 (1.08,1.09)	3.50E-89	1.06 (1.05,1.07)	9.50E-44	1.05 (1.04,1.06)	3.10E-37
symptoms	761	Cervicalgia	226921	56206	1.08 (1.07,1.09)	3.80E-52	1.07 (1.06,1.08)	2.80E-41	1.05 (1.04,1.06)	1.20E-20	1.04 (1.03,1.05)	4.20E-17
symptoms	785	Abdominal pain	206085	63494	1.09 (1.08,1.1)	2.20E-82	1.09 (1.08,1.1)	8.60E-67	1.06 (1.05,1.07)	4.70E-37	1.06 (1.05,1.07)	1.80E-34
symptoms	788	Syncope and collapse	267138	25048	1.07 (1.06,1.09)	7.40E-26	1.07 (1.05,1.08)	1.90E-18	1.04 (1.02,1.05)	2.80E-08	1.04 (1.02,1.05)	1.60E-08
symptoms	789	Nausea and vomiting	262919	21357	1.1 (1.09,1.12)	3.20E-42	1.09 (1.07,1.11)	1.10E-27	1.07 (1.05,1.08)	1.10E-18	1.07 (1.05,1.08)	5.70E-18
symptoms	798	Malaise and fatigue	223258	47019	1.08 (1.06,1.09)	8.20E-45	1.07 (1.05,1.08)	3.70E-31	1.04 (1.03,1.05)	2.20E-12	1.04 (1.03,1.05)	1.30E-12

© 2022 Bigdeli TB et al. *JAMA Psychiatry*.

eTable 21. Significant findings ($p<10^{-5}$) in PheWAS of SCZ PRS in AA participants.

Results are grouped by disease category, and numbers of controls and cases are displayed. Effect sizes and significance are reported for four models: (i) the primary analysis, adjusting for sex, age, age^2, and ancestry PCs; (ii) following exclusion individuals with SCZ-related diagnoses; (iii) adjusting for any SCZ-related diagnosis; or (iv) adjusting for lifetime treatment with antipsychotics. Odds ratios are per SD unit increase in PRS.

Group	Phecode	Description	Controls	Case	Base		Exclude Phecode 295*		Phecode-adjusted		Antipsychotic-adjusted	
					OR (95% CI)	*p*-value	OR (95% CI)	*p*-value	OR (95% CI)	*p*-value	OR (95% CI)	*p*-value
dermatologic	700	Corns and callosities	68194	9528	1.07 (1.04,1.1)	2.80E-06	1.07 (1.03,1.1)	0.00011	1.06 (1.03,1.09)	9.20E-05	1.06 (1.03,1.09)	0.00015
digestive	521	Diseases of hard tissues of teeth	48819	27048	1.06 (1.04,1.09)	3.80E-10	1.05 (1.02,1.07)	6.90E-05	1.04 (1.02,1.06)	0.00014	1.04 (1.01,1.06)	0.00064
digestive	521.1	Dental caries	49444	26480	1.06 (1.04,1.09)	7.30E-10	1.04 (1.02,1.07)	8.90E-05	1.04 (1.02,1.06)	0.00021	1.04 (1.01,1.06)	0.00092
digestive	523	Gingival and periodontal diseases	53599	22773	1.06 (1.04,1.08)	3.80E-08	1.05 (1.02,1.07)	0.00014	1.03 (1.01,1.06)	0.0015	1.03 (1.01,1.05)	0.0034
digestive	525	Other diseases of the teeth and supporting structures	47747	27265	1.08 (1.06,1.1)	4.60E-14	1.05 (1.03,1.08)	7.10E-06	1.05 (1.03,1.08)	4.50E-07	1.05 (1.03,1.07)	4.50E-06
digestive	525.1	Loss of teeth or edentulism	62014	16377	1.06 (1.03,1.08)	9.70E-07	1.04 (1.01,1.07)	0.0055	1.03 (1.01,1.06)	0.0043	1.03 (1,1.05)	0.019
infectious diseases	110	Dermatophytosis / Dermatomycosis	45593	27229	1.05 (1.03,1.07)	9.90E-07	1.05 (1.02,1.07)	3.40E-05	1.04 (1.02,1.06)	0.00042	1.04 (1.02,1.06)	0.00051
infectious diseases	110.1	Dermatophytosis	46880	26148	1.05 (1.03,1.07)	2.20E-06	1.05 (1.02,1.07)	6.10E-05	1.04 (1.02,1.06)	0.00067	1.04 (1.01,1.06)	0.00086
mental disorders	292.6	Hallucinations	81498	518	1.3 (1.16,1.45)	3.20E-06	1.03 (0.79,1.35)	0.82	1.26 (1.12,1.4)	5.20E-05	1.21 (1.08,1.35)	0.00091
mental disorders	296	Mood disorders	33552	44186	1.09 (1.06,1.11)	8.30E-17	1.07 (1.05,1.09)	1.80E-10	0.84 (0.78,0.9)	2.10E-06	1.05 (1.03,1.07)	2.10E-06
mental disorders	296.1	Bipolar	75000	5921	1.11 (1.07,1.15)	2.20E-09	1.1 (1.05,1.16)	0.00012	1.08 (1.04,1.12)	5.20E-05	1.04 (1,1.08)	0.042
mental disorders	296.2	Depression	35260	42196	1.08 (1.05,1.1)	9.20E-14	1.07 (1.05,1.09)	6.60E-10	0.9 (0.86,0.95)	6.80E-05	1.04 (1.02,1.07)	3.80E-05
mental disorders	296.22	Major depressive disorder	44614	32643	1.05 (1.03,1.07)	1.40E-07	1.05 (1.03,1.07)	3.40E-06	0.97 (0.94,1)	0.037	1.02 (1,1.04)	0.07
mental disorders	297	Suicidal ideation or attempt	72688	6386	1.1 (1.06,1.14)	6.70E-08	1.08 (1.03,1.13)	0.00099	1.06 (1.02,1.1)	0.0013	1.03 (1,1.07)	0.078
mental disorders	297.1	Suicidal ideation	74668	5503	1.1 (1.06,1.14)	6.60E-07	1.08 (1.03,1.14)	0.0032	1.06 (1.02,1.1)	0.004	1.03 (0.99,1.07)	0.16
mental disorders	300	Anxiety disorders	36301	40111	1.06 (1.04,1.08)	2.50E-08	1.04 (1.02,1.06)	0.00011	1 (0.98,1.03)	0.79	1.02 (1,1.05)	0.022
mental disorders	300.1	Anxiety disorder	51938	21915	1.06 (1.04,1.08)	6.10E-08	1.05 (1.03,1.08)	1.60E-05	1.02 (1,1.05)	0.052	1.03 (1.01,1.06)	0.0052
mental disorders	301	Personality disorders	75463	4935	1.1 (1.05,1.14)	2.20E-06	1.06 (1,1.12)	0.04	1.06 (1.02,1.1)	0.0034	1.03 (0.99,1.07)	0.17
mental disorders	304	Adjustment reaction	55082	18851	1.05 (1.03,1.08)	2.70E-06	1.05 (1.03,1.08)	3.10E-05	1.02 (1,1.04)	0.082	1.03 (1.01,1.06)	0.0029
mental disorders	316	Substance addiction and disorders	55813	22948	1.08 (1.06,1.1)	1.70E-13	1.06 (1.03,1.08)	2.30E-06	1.05 (1.03,1.07)	6.20E-06	1.04 (1.02,1.07)	8.40E-05
mental disorders	317.1	Alcoholism	58054	19278	1.07 (1.04,1.09)	5.90E-09	1.04 (1.01,1.06)	0.0035	1.04 (1.01,1.06)	0.0016	1.03 (1.01,1.06)	0.004
respiratory	465	Acute upper respiratory infections of multiple or unspecified sites	46098	21217	1.07 (1.05,1.09)	1.70E-09	1.06 (1.03,1.09)	2.20E-06	1.05 (1.03,1.07)	1.50E-05	1.05 (1.03,1.07)	2.80E-05

© 2022 Bigdeli TB et al. *JAMA Psychiatry*.

eTable 22. Significant findings (p<10^{-5}) in PheWAS of BIP PRS in AA participants.

Results are grouped by disease category, and numbers of controls and cases are displayed. Effect sizes and significance are reported for four models: (i) the primary analysis, adjusting for sex, age, age^2, and ancestry PCs; (ii) following exclusion individuals with mood disorders; (iii) adjusting for any mood-disorder diagnosis; or (iv) adjusting for lifetime treatment with mood-stabilizers. Odds ratios are per SD unit increase in PRS.

Group	Phecode	Description	Controls	Case	Base		Exclude Phecode 296*		Phecode-adjusted		Mood stabilizer-adjusted	
					OR (95% CI)	*p*-value	OR (95% CI)	*p*-value	OR (95% CI)	*p*-value	OR (95% CI)	*p*-value
digestive	521	Diseases of hard tissues of teeth	48819	27048	1.01 (1,1.01)	9.20E-11	1.01 (1,1.01)	6.30E-07	1.01 (1,1.01)	1.70E-06	1.01 (1,1.01)	3.60E-08
digestive	521.1	Dental caries	49444	26480	1.01 (1,1.01)	1.20E-09	1.01 (1,1.01)	5.50E-06	1.0 (1,1.01)	1.10E-05	1.01 (1,1.01)	3.20E-07
digestive	523	Gingival and periodontal diseases	53599	22773	1.01 (1,1.01)	2.20E-07	1.0 (1,1.01)	0.00065	1.0 (1,1.01)	0.00038	1 (1,1.01)	2.30E-05
digestive	525	Other diseases of the teeth and supporting structures	47747	27265	1.01 (1,1.01)	1.00E-09	1.0 (1,1.01)	9.80E-05	1.0 (1,1.01)	2.60E-05	1.01 (1,1.01)	2.80E-07
digestive	525.1	Loss of teeth or edentulism	62014	16377	1.01 (1,1.01)	3.90E-06	1.0 (1,1.01)	0.004	1.0 (1,1.01)	0.0021	1.0 (1,1.01)	0.00014
infectious diseases	110	Dermatophytosis / Dermatomycosis	45593	27229	1.01 (1,1.01)	5.80E-07	1.0 (1,1.01)	0.00018	1.0 (1,1.01)	5.70E-05	1.0 (1,1.01)	9.90E-06
infectious diseases	110.1	Dermatophytosis	46880	26148	1.01 (1,1.01)	7.60E-07	1.0 (1,1.01)	0.00016	1.0 (1,1.01)	6.30E-05	1.0 (1,1.01)	1.40E-05
mental disorders	296	Mood disorders	33552	44186	1.01 (1,1.01)	4.60E-09	1.0 (1,1.01)	6.30E-05	0.99 (0.98,1)	0.0033	1.0 (1,1.01)	5.20E-06
mental disorders	296.1	Bipolar	75000	5921	1.01 (1.01,1.02)	8.50E-11	1.01 (1.01,1.02)	5.60E-05	1.01 (1.01,1.01)	2.30E-07	1.01 (1.01,1.01)	1.90E-06
mental disorders	296.2	Depression	35260	42196	1.01 (1,1.01)	3.30E-07	1.0 (1,1.01)	0.00033	0.99 (0.99,1)	0.0014	1 (1,1.01)	6.50E-05
mental disorders	297	Suicidal ideation or attempt	72688	6386	1.01 (1.01,1.01)	8.50E-08	1.01 (1,1.01)	0.0024	1.01 (1,1.01)	8.00E-05	1.01 (1,1.01)	3.40E-05
mental disorders	297.1	Suicidal ideation	74668	5503	1.01 (1.01,1.01)	2.70E-06	1.01 (1,1.01)	0.0081	1.01 (1,1.01)	0.00074	1.01 (1,1.01)	0.00039
mental disorders	300	Anxiety disorders	36301	40111	1 (1,1.01)	4.00E-06	1.0 (1,1.01)	0.006	1 (1,1)	0.5	1 (1,1.01)	0.00084
mental disorders	301	Personality disorders	75463	4935	1.01 (1.01,1.01)	5.20E-07	1.01 (1,1.01)	0.024	1.01 (1,1.01)	0.00015	1.01 (1,1.01)	9.30E-05
mental disorders	306	Other mental disorder	62819	9537	1.01 (1,1.01)	1.70E-06	1.0 (1,1.01)	0.019	1.01 (1,1.01)	0.0011	1.01 (1,1.01)	0.00018
mental disorders	316	Substance addiction and disorders	55813	22948	1.01 (1,1.01)	7.30E-09	1.0 (1,1.01)	0.0013	1.0 (1,1.01)	0.00016	1.01 (1,1.01)	1.70E-06
mental disorders	317.1	Alcoholism	58054	19278	1.01 (1,1.01)	6.10E-07	1.0 (1,1.01)	0.0092	1.0 (1,1.01)	0.0013	1.0 (1,1.01)	5.10E-05
mental disorders	318	Tobacco use disorder	43079	33056	1.01 (1,1.01)	4.10E-07	1.0 (1,1.01)	0.0043	1.0 (1,1.01)	0.00017	1.0 (1,1.01)	7.20E-06
respiratory	465	Acute upper respiratory infections of multiple or unspecified sites	46098	21217	1.01 (1,1.01)	1.80E-06	1.0 (1,1.01)	0.00063	1.0 (1,1.01)	0.00042	1.0 (1,1.01)	5.40E-05
respiratory	512.8	Cough	54075	14679	1.01 (1,1.01)	9.20E-06	1.01 (1,1.01)	5.70E-05	1.0 (1,1.01)	0.00039	1.01 (1,1.01)	8.30E-05

© 2022 Bigdeli TB et al. *JAMA Psychiatry*.

eTable 23. Significant findings (p<10^{-5}) in PheWAS of DEP PRS in AA participants.

Results are grouped by disease category, and numbers of controls and cases are displayed. Effect sizes and significance are reported for four models: (i) the primary analysis, adjusting for sex, age, age^2, and ancestry PCs; (ii) following exclusion individuals with mood disorders; (iii) adjusting for any mood-disorder diagnosis; or (iv) adjusting for lifetime treatment with antidepressants. Odds ratios are per SD unit increase in PRS.

Group	Phecode	Description	Controls	Case	Base		Exclude Phecode 296*		Phecode-adjusted		Antidepressant-adjusted	
					OR (95% CI)	*p*-value	OR (95% CI)	*p*-value	OR (95% CI)	*p*-value	OR (95% CI)	*p*-value
mental disorders	296	Mood disorders	33552	44186	1.04 (1.02,1.05)	3.8E-07	1.04 (1.02,1.05)	9.0E-06	0.97 (0.91,1.02)	0.25	1.03 (1.01,1.06)	0.00053
mental disorders	296.2	Depression	35260	42196	1.04 (1.02,1.05)	1.4E-06	1.04 (1.02,1.05)	8.5E-06	0.99 (0.95,1.02)	0.49	1.03 (1.01,1.05)	0.002
mental disorders	300	Anxiety disorders	36301	40111	1.04 (1.02,1.05)	2.6E-06	1.03 (1.02,1.05)	4.9E-05	1.02 (1,1.04)	0.075	1.03 (1.01,1.05)	0.0018
mental disorders	300.1	Anxiety disorder	51938	21915	1.04 (1.02,1.06)	3.6E-06	1.04 (1.02,1.06)	4.4E-05	1.02 (1,1.04)	0.014	1.03 (1.01,1.05)	0.00059
mental disorders	300.9	Posttraumatic stress disorder	52155	26603	1.03 (1.02,1.05)	7.7E-06	1.03 (1.01,1.05)	0.00024	1.02 (1,1.04)	0.018	1.03 (1.01,1.05)	0.0012
mental disorders	306	Other mental disorder	62819	9537	1.05 (1.03,1.08)	4.7E-06	1.05 (1.02,1.07)	0.00066	1.04 (1.02,1.06)	0.00054	1.04 (1.02,1.07)	0.00012
respiratory	512	Other symptoms of respiratory system	34852	33303	1.04 (1.02,1.05)	4.4E-06	1.03 (1.01,1.05)	0.00019	1.03 (1.01,1.05)	0.00025	1.03 (1.02,1.05)	6.4E-05

© 2022 Bigdeli TB et al. *JAMA Psychiatry*.

eTable 24. Significant findings ($p<10^{-25}$) in PheWAS of SCZ-specific PRS in EA participants.
Results are grouped by disease category, and numbers of controls and cases are displayed. Effect sizes and significance are reported for four models: (i) the primary analysis, adjusting for sex, age, age^2, and ancestry PCs; (ii) following exclusion individuals with SCZ-related diagnoses; (iii) adjusting for any SCZ-related diagnosis; or (iv) adjusting for lifetime treatment with antipsychotics. Odds ratios are per SD unit increase in PRS.

Group	Phecode	Description	Controls	Case	Base		Exclude phecode 295*		Phecode-adjusted		Antipsychotic-adjusted	
					OR (95% CI)	*p*-value	OR (95% CI)	*p*-value	OR (95% CI)	*p*-value	OR (95% CI)	*p*-value
circulatory system	411	Ischemic Heart Disease	187667	100858	0.98 (0.97,0.98)	1.9E-26	0.98 (0.97,0.98)	3.2E-24	0.98 (0.97,0.98)	5.5E-23	0.98 (0.97,0.98)	8.0E-29
digestive	530	Diseases of esophagus	153615	131627	0.98 (0.97,0.98)	1.9E-32	0.98 (0.97,0.98)	5.6E-34	0.98 (0.97,0.98)	2.3E-26	0.97 (0.97,0.98)	1.9E-36
digestive	530.1	Esophagitis, GERD and related diseases	156612	128556	0.98 (0.97,0.98)	1.1E-34	0.97 (0.97,0.98)	1.7E-35	0.98 (0.97,0.98)	2.0E-28	0.97 (0.97,0.98)	1.0E-38
digestive	530.11	GERD	162469	122039	0.98 (0.97,0.98)	2.3E-35	0.97 (0.97,0.98)	1.2E-35	0.98 (0.97,0.98)	5.2E-29	0.97 (0.97,0.98)	2.6E-39
mental disorders	296.22	Major depressive disorder	200712	92217	0.98 (0.97,0.98)	4.2E-29	0.97 (0.97,0.98)	4.1E-34	0.97 (0.97,0.98)	4.7E-18	0.97 (0.96,0.97)	7.4E-41
musculoskeletal	721	Spondylosis and allied disorders	238955	44356	0.97 (0.96,0.97)	1.4E-29	0.97 (0.97,0.98)	1.1E-23	0.97 (0.97,0.98)	3.0E-25	0.97 (0.96,0.97)	2.0E-32
musculoskeletal	721.1	Spondylosis without myelopathy	242860	40805	0.97 (0.96,0.97)	1.6E-30	0.97 (0.96,0.98)	2.5E-24	0.97 (0.96,0.98)	4.3E-26	0.97 (0.96,0.97)	2.4E-33
musculoskeletal	722	Intervertebral disc disorders	228596	55337	0.97 (0.96,0.97)	1.1E-41	0.97 (0.96,0.97)	3.3E-34	0.97 (0.96,0.97)	1.3E-36	0.96 (0.96,0.97)	2.4E-45
musculoskeletal	722.6	Degeneration of intervertebral disc	242906	42226	0.96 (0.96,0.97)	1.7E-37	0.97 (0.96,0.97)	4.7E-32	0.97 (0.96,0.97)	4.2E-33	0.96 (0.96,0.97)	1.8E-40
musculoskeletal	726	Peripheral enthesopathies and allied syndromes	196831	75403	0.97 (0.96,0.97)	6.3E-44	0.97 (0.97,0.97)	2.0E-36	0.97 (0.97,0.97)	9.6E-39	0.97 (0.96,0.97)	9.3E-47
musculoskeletal	740	Osteoarthrosis	146222	131442	0.97 (0.97,0.98)	8.0E-44	0.97 (0.97,0.98)	8.6E-38	0.97 (0.97,0.98)	3.1E-38	0.97 (0.97,0.97)	4.3E-47
musculoskeletal	740.1	Osteoarthritis; localized	205924	74421	0.98 (0.97,0.98)	2.4E-27	0.98 (0.97,0.98)	3.1E-23	0.98 (0.97,0.98)	2.1E-24	0.98 (0.97,0.98)	1.6E-28
musculoskeletal	740.9	Osteoarthrosis NOS	179236	99593	0.97 (0.97,0.98)	2.5E-37	0.97 (0.97,0.98)	4.2E-33	0.97 (0.97,0.98)	3.9E-32	0.97 (0.97,0.98)	4.5E-41
musculoskeletal	745	Pain in joint	89094	187207	0.98 (0.97,0.98)	7.8E-29	0.98 (0.97,0.98)	4.7E-28	0.98 (0.97,0.98)	4.4E-23	0.97 (0.97,0.98)	2.5E-32
neurological	327.3	Sleep apnea	195189	101623	0.97 (0.96,0.97)	6.9E-65	0.97 (0.96,0.97)	1.3E-57	0.97 (0.96,0.97)	2.0E-57	0.96 (0.96,0.97)	1.9E-68
neurological	327.32	Obstructive sleep apnea	213928	82508	0.97 (0.96,0.97)	2.4E-54	0.97 (0.96,0.97)	3.3E-49	0.97 (0.96,0.97)	3.0E-48	0.97 (0.96,0.97)	6.4E-57
neurological	351	Other peripheral nerve disorders	227740	57720	0.97 (0.96,0.97)	1.0E-43	0.97 (0.96,0.97)	1.1E-40	0.97 (0.96,0.97)	1.5E-38	0.96 (0.96,0.97)	2.2E-47
sense organs	389	Hearing loss	121761	165684	0.97 (0.97,0.97)	5.4E-45	0.97 (0.97,0.98)	1.1E-37	0.97 (0.97,0.98)	3.8E-41	0.97 (0.97,0.97)	2.5E-46
sense organs	389.1	Sensorineural hearing loss	143787	132938	0.97 (0.96,0.97)	7.4E-55	0.97 (0.96,0.97)	3.0E-47	0.97 (0.96,0.97)	6.8E-51	0.97 (0.96,0.97)	1.5E-56
symptoms	760	Back pain	128684	149486	0.98 (0.97,0.98)	2.0E-31	0.98 (0.97,0.98)	1.2E-30	0.98 (0.97,0.98)	1.8E-25	0.97 (0.97,0.98)	2.5E-36

© 2022 Bigdeli TB et al. *JAMA Psychiatry*.

eTable 25. Significant findings ($p<10^{-5}$) in PheWAS of SCZ-specific PRS in AA participants.

Results are grouped by disease category, and numbers of controls and cases are displayed. Effect sizes and significance are reported for four models: (i) the primary analysis, adjusting for sex, age, age^2, and ancestry PCs; (ii) following exclusion individuals with SCZ-related diagnoses; (iii) adjusting for any SCZ-related diagnosis; or (iv) adjusting for lifetime treatment with antipsychotics. Odds ratios are per SD unit increase in PRS.

Group	Phecode	Description	Controls	Case	Base		Exclude Phecode 295*		Phecode-adjusted		Antipsychotic-adjusted	
					OR (95% CI)	*p*-value	OR (95% CI)	*p*-value	OR (95% CI)	*p*-value	OR (95% CI)	*p*-value
dermatologic	687.4	Disturbance of skin sensation	62795	8986	0.98 (0.97,0.99)	3.6E-06	0.98 (0.97,0.99)	6.7E-05	0.98 (0.97,0.99)	3.9E-06	0.98 (0.97,0.99)	2.4E-06
neurological	351	Other peripheral nerve disorders	59576	15819	0.98 (0.98,0.99)	2.5E-06	0.99 (0.98,0.99)	9.0E-05	0.98 (0.98,0.99)	1.9E-06	0.98 (0.98,0.99)	9.1E-07

© 2022 Bigdeli TB et al. *JAMA Psychiatry*.

eTable 26. Significant findings ($p<10^{-25}$) in PheWAS of BIP-specific PRS in EA participants.

Results are grouped by disease category, and numbers of controls and cases are displayed. Effect sizes and significance are reported for four models: (i) the primary analysis, adjusting for sex, age, age^2, and ancestry PCs; (ii) following exclusion individuals with mood disorders; (iii) adjusting for any mood-disorder diagnosis; or (iv) adjusting for lifetime treatment with mood-stabilizers. Odds ratios are per SD unit increase in PRS. **NB:** there were no significant results in the analysis of BIP-specific PRS in AA participants.

Group	Phecode	Description	Controls	Case	Base		Exclude Phecode 296*		Phecode-adjusted		Mood stabilizer-adjusted	
					OR (95% CI)	*p*-value	OR (95% CI)	*p*-value	OR (95% CI)	*p*-value	OR (95% CI)	*p*-value
circulatory system	418	Nonspecific chest pain	178521	91397	0.98 (0.98,0.99)	1.9E-26	0.98 (0.98,0.99)	7.6E-24	0.99 (0.98,0.99)	1.2E-16	0.98 (0.98,0.99)	5.3E-25
mental disorders	296	Mood disorders	161370	132442	0.98 (0.98,0.99)	9.6E-28	0.98 (0.98,0.99)	1.5E-27	0.99 (0.98,1.01)	4.6E-01	0.98 (0.98,0.99)	1.6E-26
mental disorders	296.2	Depression	167868	124718	0.98 (0.98,0.98)	2.6E-35	0.98 (0.98,0.98)	1.0E-32	0.98 (0.97,0.99)	7.9E-07	0.98 (0.98,0.98)	2.9E-33
mental disorders	296.22	Major depressive disorder	200712	92217	0.98 (0.98,0.99)	4.6E-26	0.98 (0.98,0.99)	1.5E-23	0.99 (0.99,1)	3.4E-04	0.98 (0.98,0.99)	9.0E-24
mental disorders	300	Anxiety disorders	166912	123000	0.98 (0.98,0.98)	2.7E-36	0.98 (0.98,0.98)	2.3E-34	0.98 (0.98,0.99)	1.2E-14	0.98 (0.98,0.98)	1.3E-34
symptoms	785	Abdominal pain	206085	63494	0.98 (0.98,0.98)	1.3E-26	0.98 (0.98,0.98)	6.3E-22	0.98 (0.98,0.99)	2.8E-18	0.98 (0.98,0.98)	1.0E-25

eTable 27. Significant findings ($p<10^{-25}$) in PheWAS of DEP-specific PRS in EA participants.

Results are grouped by disease category, and numbers of controls and cases are displayed. Effect sizes and significance are reported for four models: (i) the primary analysis, adjusting for sex, age, age^2, and ancestry PCs; (ii) following exclusion individuals with mood disorders; (iii) adjusting for any mood-disorder diagnosis; or (iv) adjusting for lifetime treatment with antidepressants. Odds ratios are per SD unit increase in PRS.

Group	Phecode	Description	Controls	Case	Base		Exclude Phecode 296*		Phecode-adjusted		Antidepressant-adjusted	
					OR (95% CI)	*p*-value	OR (95% CI)	*p*-value	OR (95% CI)	*p*-value	OR (95% CI)	*p*-value
circulatory system	401	Hypertension	69818	225389	1.04 (1.04,1.05)	1.5E-31	1.05 (1.04,1.05)	4.1E-32	1.04 (1.03,1.04)	4.6E-22	1.03 (1.02,1.04)	7.7E-18
circulatory system	401.1	Essential hypertension	70815	224179	1.04 (1.04,1.05)	1.7E-31	1.05 (1.04,1.05)	1.2E-31	1.04 (1.03,1.04)	4.3E-22	1.03 (1.02,1.04)	7.2E-18
circulatory system	411	Ischemic Heart Disease	187667	100858	1.06 (1.05,1.06)	3.4E-64	1.06 (1.05,1.06)	7.9E-60	1.05 (1.04,1.06)	3.2E-50	1.05 (1.04,1.05)	3.5E-45
circulatory system	411.3	Angina pectoris	265965	26054	1.06 (1.05,1.07)	4.2E-31	1.06 (1.05,1.07)	1.2E-26	1.05 (1.04,1.07)	1.1E-23	1.05 (1.04,1.06)	2.2E-21
circulatory system	411.4	Coronary atherosclerosis	205059	88022	1.05 (1.05,1.06)	5.0E-56	1.05 (1.05,1.06)	3.1E-50	1.05 (1.04,1.06)	1.6E-44	1.05 (1.04,1.05)	3.9E-40
circulatory system	411.8	Other chronic ischemic heart disease, unspecified	242159	53680	1.05 (1.04,1.06)	1.0E-37	1.05 (1.04,1.06)	1.4E-33	1.05 (1.04,1.05)	3.2E-30	1.04 (1.04,1.05)	5.7E-27
circulatory system	418	Nonspecific chest pain	178521	91397	1.05 (1.04,1.06)	6.7E-52	1.05 (1.04,1.06)	7.8E-49	1.04 (1.03,1.05)	1.3E-30	1.03 (1.03,1.04)	3.6E-24
digestive	530	Diseases of esophagus	153615	131627	1.06 (1.05,1.07)	4.8E-83	1.06 (1.05,1.07)	1.1E-80	1.05 (1.05,1.06)	9.5E-60	1.05 (1.04,1.05)	2.5E-51
digestive	530.1	Esophagitis, GERD and related diseases	156612	128556	1.06 (1.05,1.07)	7.5E-86	1.06 (1.06,1.07)	4.2E-83	1.05 (1.05,1.06)	2.6E-62	1.05 (1.04,1.06)	1.1E-53

© 2022 Bigdeli TB et al. *JAMA Psychiatry*.

digestive	530.11	GERD	162469	122039	1.06 (1.05,1.07)	3.0E-85	1.06 (1.06,1.07)	2.1E-82	1.05 (1.05,1.06)	3.7E-62	1.05 (1.04,1.06)	1.2E-53
digestive	561	Symptoms involving digestive system	233209	41830	1.05 (1.04,1.06)	1.2E-28	1.05 (1.04,1.06)	1.0E-30	1.04 (1.03,1.04)	5.9E-16	1.03 (1.02,1.04)	2.4E-13
endocrine/metabolic	250	Diabetes mellitus	189084	108545	1.04 (1.04,1.05)	3.3E-42	1.04 (1.04,1.05)	6.4E-39	1.04 (1.03,1.04)	6.7E-33	1.03 (1.03,1.04)	9.8E-29
endocrine/metabolic	250.2	Type 2 diabetes	190540	107822	1.04 (1.04,1.05)	5.8E-42	1.04 (1.04,1.05)	1.2E-38	1.04 (1.03,1.04)	9.4E-33	1.03 (1.03,1.04)	1.1E-28
endocrine/metabolic	250.24	Type 2 diabetes with neurological manifestations	259077	40530	1.06 (1.05,1.07)	2.4E-40	1.06 (1.05,1.07)	4.2E-38	1.05 (1.04,1.06)	4.3E-30	1.05 (1.04,1.05)	4.1E-25
endocrine/metabolic	272	Disorders of lipoid metabolism	60613	234106	1.04 (1.03,1.05)	1.1E-27	1.04 (1.03,1.05)	2.2E-25	1.04 (1.03,1.04)	1.9E-20	1.03 (1.02,1.04)	4.5E-17
endocrine/metabolic	272.1	Hyperlipidemia	60815	233903	1.04 (1.03,1.05)	5.8E-28	1.04 (1.03,1.05)	1.2E-25	1.04 (1.03,1.04)	1.1E-20	1.03 (1.02,1.04)	2.9E-17
endocrine/metabolic	278	Overweight, obesity and other hyperalimentation	151774	133275	1.04 (1.03,1.04)	1.1E-35	1.04 (1.03,1.05)	3.2E-35	1.03 (1.03,1.04)	3.8E-25	1.03 (1.02,1.04)	9.3E-23
endocrine/metabolic	278.1	Obesity	169074	119822	1.04 (1.04,1.05)	1.7E-41	1.04 (1.04,1.05)	4.4E-41	1.04 (1.03,1.04)	4.2E-30	1.03 (1.03,1.04)	2.0E-27
endocrine/metabolic	278.11	Morbid obesity	265927	32713	1.05 (1.04,1.06)	4.7E-27	1.05 (1.04,1.06)	5.8E-27	1.04 (1.03,1.05)	2.9E-20	1.04 (1.03,1.05)	7.6E-19
injuries & poisonings	840	Sprains and strains	238860	36387	1.05 (1.04,1.06)	4.0E-28	1.05 (1.04,1.06)	3.5E-26	1.04 (1.03,1.05)	1.9E-19	1.04 (1.03,1.05)	2.5E-16
mental disorders	296	Mood disorders	161370	132442	1.06 (1.05,1.06)	1.8E-73	1.07 (1.06,1.08)	1.9E-96	1.14 (1.1,1.18)	6.3E-16	1.02 (1.01,1.03)	2.2E-08
mental disorders	296.2	Depression	167868	124718	1.07 (1.06,1.07)	6.5E-96	1.07 (1.07,1.08)	6.0E-108	1.12 (1.1,1.14)	5.8E-38	1.04 (1.03,1.05)	1.5E-19
mental disorders	296.22	Major depressive disorder	200712	92217	1.07 (1.06,1.08)	4.6E-90	1.07 (1.07,1.08)	1.0E-93	1.06 (1.05,1.07)	1.4E-29	1.04 (1.03,1.05)	3.2E-22
mental disorders	300	Anxiety disorders	166912	123000	1.05 (1.04,1.05)	5.9E-47	1.06 (1.05,1.06)	1.1E-58	1.02 (1.02,1.03)	3.1E-09	1.01 (1.01,1.02)	3.0E-04
mental disorders	300.1	Anxiety disorder	206217	79142	1.04 (1.03,1.05)	3.5E-27	1.05 (1.04,1.05)	1.1E-34	1.01 (1.01,1.02)	1.3E-04	1.01 (1,1.01)	6.9E-02
mental disorders	300.9	Posttraumatic stress disorder	230809	69537	1.04 (1.04,1.05)	1.6E-31	1.05 (1.04,1.06)	6.1E-35	1.02 (1.01,1.03)	1.6E-08	1.02 (1.01,1.02)	2.8E-05
musculoskeletal	716	Other arthropathies	247336	33669	1.05 (1.04,1.06)	4.3E-28	1.05 (1.04,1.07)	8.3E-28	1.04 (1.03,1.05)	4.6E-20	1.04 (1.03,1.05)	6.3E-17
musculoskeletal	716.9	Arthropathy NOS	258687	26329	1.06 (1.05,1.07)	6.8E-28	1.06 (1.05,1.07)	3.4E-27	1.05 (1.04,1.06)	3.5E-20	1.05 (1.03,1.06)	2.7E-17
musculoskeletal	721	Spondylosis and allied disorders	238955	44356	1.06 (1.05,1.07)	4.5E-46	1.06 (1.05,1.07)	5.9E-39	1.05 (1.04,1.06)	4.0E-32	1.04 (1.04,1.05)	1.8E-25
musculoskeletal	721.1	Spondylosis without myelopathy	242860	40805	1.06 (1.05,1.07)	8.2E-45	1.06 (1.05,1.07)	2.4E-37	1.05 (1.04,1.06)	2.4E-31	1.05 (1.04,1.06)	4.7E-25
musculoskeletal	722	Intervertebral disc disorders	228596	55337	1.06 (1.05,1.07)	4.7E-56	1.06 (1.05,1.07)	2.9E-46	1.05 (1.04,1.06)	1.7E-40	1.05 (1.04,1.05)	4.3E-32
musculoskeletal	722.6	Degeneration of intervertebral disc	242906	42226	1.06 (1.05,1.07)	8.3E-44	1.06 (1.05,1.07)	2.1E-36	1.05 (1.04,1.06)	8.4E-32	1.05 (1.04,1.05)	1.9E-25
musculoskeletal	726	Peripheral enthesopathies and allied syndromes	196831	75403	1.06 (1.05,1.06)	9.4E-60	1.06 (1.05,1.06)	1.0E-50	1.05 (1.04,1.06)	1.1E-44	1.05 (1.04,1.05)	1.0E-37
musculoskeletal	727	Other disorders of synovium, tendon, and bursa	245069	38207	1.06 (1.05,1.06)	1.2E-34	1.05 (1.04,1.06)	1.2E-28	1.05 (1.04,1.06)	2.4E-26	1.04 (1.04,1.05)	1.4E-22

© 2022 Bigdeli TB et al. *JAMA Psychiatry*.

musculoskeletal	740	Osteoarthrosis	146222	131442	1.06 (1.05,1.07)	1.4E-82	1.06 (1.05,1.07)	5.0E-73	1.05 (1.05,1.06)	2.5E-62	1.05 (1.04,1.06)	4.4E-53
musculoskeletal	740.1	Osteoarthritis; localized	205924	74421	1.05 (1.04,1.05)	2.2E-42	1.05 (1.04,1.05)	1.1E-35	1.04 (1.03,1.05)	8.0E-33	1.04 (1.03,1.05)	3.3E-28
musculoskeletal	740.11	Osteoarthrosis, localized, primary	238007	48589	1.05 (1.04,1.06)	9.2E-33	1.05 (1.04,1.05)	1.8E-27	1.04 (1.03,1.05)	2.9E-26	1.04 (1.03,1.05)	5.1E-23
musculoskeletal	740.9	Osteoarthrosis NOS	179236	99593	1.06 (1.05,1.06)	4.6E-67	1.06 (1.05,1.06)	1.3E-60	1.05 (1.04,1.06)	4.6E-49	1.04 (1.04,1.05)	4.0E-41
musculoskeletal	745	Pain in joint	89094	187207	1.05 (1.05,1.06)	3.6E-55	1.05 (1.05,1.06)	1.9E-51	1.04 (1.04,1.05)	2.7E-36	1.04 (1.03,1.05)	1.0E-28
neurological	327	Sleep disorders	191386	81129	1.05 (1.04,1.06)	7.1E-48	1.05 (1.05,1.06)	1.3E-47	1.04 (1.03,1.04)	2.2E-22	1.03 (1.02,1.04)	9.7E-16
neurological	327.3	Sleep apnea	195189	101623	1.06 (1.05,1.06)	4.6E-69	1.05 (1.05,1.06)	2.4E-59	1.05 (1.04,1.05)	1.7E-47	1.04 (1.04,1.05)	1.2E-41
neurological	327.32	Obstructive sleep apnea	213928	82508	1.05 (1.05,1.06)	3.0E-54	1.05 (1.04,1.06)	1.7E-46	1.04 (1.04,1.05)	2.2E-37	1.04 (1.03,1.05)	4.7E-32
neurological	338	Pain	225658	47039	1.06 (1.05,1.07)	2.6E-47	1.06 (1.05,1.07)	1.0E-43	1.05 (1.04,1.06)	1.5E-28	1.04 (1.03,1.05)	8.6E-22
neurological	338.2	Chronic pain	248178	34632	1.06 (1.05,1.07)	2.8E-40	1.06 (1.05,1.08)	1.3E-36	1.05 (1.04,1.06)	3.2E-24	1.04 (1.03,1.05)	1.6E-18
neurological	339	Other headache syndromes	237942	42426	1.07 (1.06,1.08)	2.2E-50	1.07 (1.06,1.08)	2.5E-47	1.05 (1.05,1.06)	6.7E-33	1.05 (1.04,1.06)	7.0E-26
neurological	340	Migraine	280876	21151	1.08 (1.07,1.09)	4.9E-36	1.08 (1.06,1.09)	6.3E-30	1.07 (1.05,1.08)	3.2E-27	1.06 (1.05,1.07)	2.0E-22
neurological	350	Abnormal movement	220347	57430	1.04 (1.04,1.05)	1.9E-29	1.05 (1.04,1.06)	1.2E-33	1.03 (1.02,1.04)	2.7E-14	1.03 (1.02,1.03)	1.6E-11
neurological	351	Other peripheral nerve disorders	227740	57720	1.07 (1.06,1.07)	2.5E-65	1.06 (1.06,1.07)	2.3E-55	1.06 (1.05,1.06)	1.7E-48	1.05 (1.04,1.06)	6.5E-39
neurological	355.1	Chronic pain syndrome	283361	17187	1.08 (1.06,1.09)	2.3E-30	1.07 (1.06,1.09)	3.3E-26	1.06 (1.05,1.07)	9.4E-20	1.05 (1.04,1.07)	1.6E-15
respiratory	496	Chronic airway obstruction	215720	72353	1.06 (1.05,1.07)	1.6E-60	1.06 (1.06,1.07)	3.4E-65	1.05 (1.04,1.06)	2.5E-43	1.05 (1.04,1.05)	2.5E-38
respiratory	496.2	Chronic bronchitis	276924	19677	1.07 (1.05,1.08)	1.3E-26	1.07 (1.06,1.09)	3.1E-28	1.05 (1.04,1.07)	1.0E-18	1.05 (1.04,1.06)	8.1E-17
respiratory	512	Other symptoms of respiratory system	139662	120187	1.05 (1.05,1.06)	1.2E-58	1.05 (1.05,1.06)	4.6E-58	1.04 (1.03,1.05)	4.8E-36	1.04 (1.03,1.04)	1.0E-28
respiratory	512.7	Shortness of breath	209689	60907	1.05 (1.05,1.06)	4.6E-44	1.05 (1.05,1.06)	1.1E-40	1.04 (1.03,1.05)	2.1E-28	1.04 (1.03,1.05)	6.6E-24
respiratory	512.9	Other dyspnea	219490	48646	1.05 (1.05,1.06)	1.5E-39	1.06 (1.05,1.06)	3.9E-37	1.04 (1.04,1.05)	3.0E-27	1.04 (1.03,1.05)	4.7E-23
sense organs	386.9	Dizziness and giddiness (Light-headedness and vertigo)	231860	43001	1.05 (1.05,1.06)	7.0E-36	1.05 (1.04,1.06)	4.2E-31	1.04 (1.03,1.05)	1.7E-22	1.04 (1.03,1.05)	1.7E-19
sense organs	389	Hearing loss	121761	165684	1.04 (1.04,1.05)	1.5E-40	1.04 (1.03,1.05)	3.3E-33	1.04 (1.03,1.04)	1.2E-31	1.04 (1.03,1.04)	5.5E-29
sense organs	389.1	Sensorineural hearing loss	143787	132938	1.05 (1.04,1.06)	1.7E-48	1.05 (1.04,1.05)	4.1E-40	1.04 (1.04,1.05)	2.5E-39	1.04 (1.04,1.05)	4.4E-36
symptoms	760	Back pain	128684	149486	1.06 (1.05,1.07)	5.6E-81	1.06 (1.06,1.07)	4.6E-80	1.05 (1.04,1.06)	3.3E-54	1.04 (1.04,1.05)	2.4E-42
symptoms	761	Cervicalgia	226921	56206	1.06 (1.05,1.06)	1.2E-46	1.06 (1.05,1.06)	9.4E-42	1.05 (1.04,1.05)	8.6E-31	1.04 (1.03,1.05)	2.1E-24
symptoms	763	Thoracic or lumbosacral neuritis	263827	29935	1.06 (1.05,1.07)	1.1E-31	1.06 (1.05,1.07)	2.2E-26	1.05 (1.04,1.06)	2.9E-22	1.04 (1.03,1.05)	7.6E-17

© 2022 Bigdeli TB et al. *JAMA Psychiatry*.

		or radiculitis, unspecified										
symptoms	765	Cervical radiculitis	282740	16245	1.07 (1.06,1.08)	8.7E-26	1.07 (1.06,1.08)	5.4E-23	1.06 (1.05,1.07)	1.8E-19	1.05 (1.04,1.07)	7.4E-16
symptoms	770	Myalgia and myositis unspecified	273265	19134	1.07 (1.06,1.08)	7.6E-29	1.07 (1.05,1.08)	7.2E-24	1.06 (1.04,1.07)	1.1E-19	1.05 (1.04,1.06)	4.9E-16
symptoms	785	Abdominal pain	206085	63494	1.05 (1.05,1.06)	2.2E-45	1.05 (1.05,1.06)	1.4E-41	1.04 (1.03,1.05)	5.5E-28	1.04 (1.03,1.05)	1.9E-22

© 2022 Bigdeli TB et al. *JAMA Psychiatry*.

eTable 28. Significant findings (*p*<10^{-5}) in PheWAS of DEP-specific PRS in AA participants.
Results are grouped by disease category, and numbers of controls and cases are displayed. Effect sizes and significance are reported for four models: (i) the primary analysis, adjusting for sex, age, age^2, and ancestry PCs; (ii) following exclusion individuals with mood disorders; (iii) adjusting for any mood-disorder diagnosis; or (iv) adjusting for lifetime treatment with antidepressants. Odds ratios are per SD unit increase in PRS.

Group	Phecode	Description	Controls	Case	Base		Exclude Phecode 296*		Phecode-adjusted		Antidepressant-adjusted	
					OR (95% CI)	*p*-value	OR (95% CI)	*p*-value	OR (95% CI)	*p*-value	OR (95% CI)	*p*-value
neurological	339	Other headache syndromes	54393	17396	1.03 (1.02,1.04)	6.2E-08	1.02 (1.01,1.03)	1.4E-05	1.03 (1.02,1.04)	2.1E-07	1.02 (1.01,1.03)	8.4E-07

© 2022 Bigdeli TB et al. *JAMA Psychiatry*.

eTable 29. Significant findings (*p*<10^{-25}) in PheWAS of common factor PRS in EA participants.

Results are grouped by disease category, and numbers of controls and cases are displayed. Effect sizes and significance are reported for six models: (i) the primary analysis, adjusting for sex, age, age^2, and ancestry PCs; (ii) following exclusion individuals with psychotic or mood disorders; (iii) adjusting for any diagnosis of a psychotic or mood-disorder (separately); or (iv) adjusting for lifetime treatment with antipsychotics; (v) adjusting for lifetime treatment with mood stabilizers; (vi) adjusting for lifetime treatment with antidepressants. Odds ratios are per SD unit increase in PRS.

Group	Phe-code	Description	Cont	Case	Base		Exclude Phecode 295*		Phecode-adjusted		Antipsychotic-adjusted		Mood stabilizer-adjusted		Antidepressant-adjusted	
					OR (95% CI)	*p*-value	OR (95% CI)	*p*-value	OR (95% CI)	*p*-value	OR (95% CI)	*p*-value	OR (95% CI)	*p*-value	OR (95% CI)	*p*-value
circulatory system	401	Hypertension	69818	225389	1.03 (1.02,1.03)	2.2E-31	1.02 (1.02,1.03)	5.9E-25	1.01 (1.01,1.02)	2.1E-07	1.02 (1.01,1.02)	2.2E-15	1.02 (1.02,1.03)	2.1E-20	1.01 (1.01,1.02)	1.1E-06
circulatory system	401.1	Essential hypertension	70815	224179	1.03 (1.02,1.03)	2.6E-30	1.02 (1.02,1.03)	3.6E-24	1.01 (1.01,1.02)	4.6E-07	1.02 (1.01,1.02)	7.6E-15	1.02 (1.02,1.03)	1.3E-19	1.01 (1.01,1.02)	2.4E-06
circulatory system	411	Ischemic Heart Disease	187667	100858	1.03 (1.03,1.04)	5.4E-51	1.03 (1.02,1.03)	2.7E-42	1.02 (1.01,1.02)	4.8E-19	1.02 (1.02,1.03)	1.3E-30	1.03 (1.02,1.03)	1.6E-39	1.02 (1.01,1.02)	8.6E-18
circulatory system	411.1	Unstable angina (intermediate coronary syndrome)	287650	11889	1.06 (1.05,1.07)	8.0E-32	1.06 (1.05,1.07)	3.8E-30	1.04 (1.03,1.05)	1.2E-14	1.04 (1.04,1.05)	5.3E-21	1.05 (1.04,1.06)	1.0E-24	1.04 (1.03,1.05)	3.7E-14
circulatory system	411.3	Angina pectoris	265965	26054	1.04 (1.03,1.05)	1.6E-34	1.04 (1.03,1.05)	2.4E-28	1.02 (1.02,1.03)	7.8E-13	1.03 (1.03,1.04)	5.4E-22	1.03 (1.03,1.04)	3.3E-26	1.02 (1.02,1.03)	8.4E-13
circulatory system	411.4	Coronary atherosclerosis	205059	88022	1.03 (1.02,1.03)	3.3E-33	1.02 (1.02,1.03)	4.8E-28	1.01 (1.01,1.02)	2.4E-11	1.02 (1.02,1.02)	2.8E-20	1.02 (1.02,1.03)	1.5E-26	1.01 (1.01,1.02)	2.7E-10
circulatory system	418	Nonspecific chest pain	178521	91397	1.06 (1.05,1.06)	3.0E-174	1.05 (1.05,1.06)	2.0E-130	1.04 (1.03,1.04)	3.0E-64	1.04 (1.04,1.05)	9.4E-99	1.05 (1.05,1.05)	6.3E-121	1.04 (1.03,1.04)	1.7E-65
circulatory system	427	Cardiac dysrhythmias	168977	104053	1.03 (1.02,1.03)	1.9E-39	1.02 (1.02,1.03)	1.6E-27	1.01 (1.01,1.02)	5.8E-10	1.02 (1.01,1.02)	5.0E-16	1.02 (1.02,1.02)	9.2E-25	1.01 (1.01,1.02)	5.1E-10
circulatory system	428	Congestive heart failure; non-hypertensive	254758	41526	1.03 (1.02,1.04)	2.6E-28	1.03 (1.02,1.03)	4.7E-22	1.01 (1.01,1.02)	7.8E-08	1.02 (1.01,1.03)	3.2E-13	1.03 (1.02,1.03)	1.9E-22	1.01 (1.01,1.02)	7.7E-08
circulatory system	433	Cerebrovascular disease	239598	49889	1.03 (1.03,1.04)	6.2E-34	1.03 (1.02,1.03)	4.0E-27	1.01 (1.01,1.02)	3.1E-08	1.02 (1.01,1.03)	1.7E-15	1.02 (1.02,1.03)	6.8E-20	1.01 (1.01,1.02)	6.6E-09
circulatory system	455	Hemorrhoids	222924	42032	1.03 (1.02,1.03)	1.9E-27	1.03 (1.02,1.03)	3.7E-21	1.02 (1.01,1.02)	3.4E-10	1.02 (1.02,1.03)	2.3E-15	1.02 (1.02,1.03)	2.6E-17	1.02 (1.01,1.02)	4.5E-10
circulatory system	458	Hypotension	256763	27983	1.04 (1.03,1.05)	7.7E-35	1.03 (1.02,1.04)	8.0E-20	1.01 (1.01,1.02)	3.2E-05	1.02 (1.01,1.02)	3.0E-08	1.03 (1.02,1.04)	2.4E-19	1.01 (1.01,1.02)	8.4E-06
Dermato-logic	681	Superficial cellulitis and abscess	224657	52705	1.04 (1.03,1.04)	1.7E-54	1.03 (1.03,1.04)	1.3E-34	1.02 (1.01,1.02)	1.8E-14	1.02 (1.02,1.03)	2.2E-20	1.03 (1.02,1.03)	7.2E-32	1.02 (1.01,1.02)	1.5E-15
Dermato-logic	687.1	Rash and other nonspecific skin eruption	236181	30351	1.04 (1.03,1.05)	2.3E-37	1.03 (1.03,1.04)	3.6E-24	1.02 (1.02,1.03)	8.9E-14	1.03 (1.02,1.03)	1.5E-19	1.03 (1.02,1.04)	8.8E-24	1.02 (1.02,1.03)	4.1E-14
digestive	521	Diseases of hard tissues of teeth	233809	60055	1.05 (1.05,1.06)	3.6E-118	1.05 (1.04,1.05)	3.3E-73	1.03 (1.02,1.03)	1.0E-30	1.03 (1.03,1.04)	3.8E-43	1.04 (1.04,1.05)	2.5E-64	1.03 (1.03,1.04)	1.2E-36
digestive	521.1	Dental caries	236323	58432	1.05 (1.05,1.06)	3.6E-116	1.05 (1.04,1.05)	9.0E-72	1.03 (1.02,1.03)	2.3E-30	1.03 (1.03,1.04)	2.1E-42	1.04 (1.04,1.05)	4.0E-63	1.03 (1.03,1.04)	2.7E-36
digestive	522	Diseases of pulp and periapical tissues	284618	14361	1.06 (1.05,1.07)	1.1E-45	1.05 (1.04,1.06)	7.1E-29	1.04 (1.03,1.04)	9.5E-16	1.04 (1.03,1.05)	2.3E-18	1.05 (1.04,1.05)	1.2E-24	1.04 (1.03,1.05)	1.2E-17
digestive	522.1	Pulpitis and necrosis of tooth pulp	294641	8437	1.07 (1.06,1.08)	3.7E-37	1.06 (1.05,1.08)	2.9E-23	1.04 (1.03,1.06)	5.1E-15	1.05 (1.04,1.06)	1.1E-16	1.05 (1.04,1.07)	5.3E-21	1.05 (1.04,1.06)	1.8E-16

© 2022 Bigdeli TB et al. *JAMA Psychiatry*.

digestive	523	Gingival and periodontal diseases	246246	49998	1.05 (1.05,1.06)	4.4E-95	1.04 (1.04,1.05)	2.3E-59	1.03 (1.02,1.03)	5.1E-24	1.03 (1.03,1.04)	1.3E-33	1.04 (1.03,1.04)	3.1E-49	1.03 (1.02,1.03)	5.0E-28
digestive	523.1	Gingivitis	281815	17735	1.05 (1.05,1.06)	5.9E-41	1.04 (1.04,1.05)	5.0E-25	1.03 (1.02,1.04)	4.4E-12	1.03 (1.02,1.04)	3.0E-16	1.04 (1.03,1.04)	3.1E-20	1.03 (1.02,1.04)	2.2E-13
digestive	523.3	Periodontitis (acute or chronic)	271618	25654	1.06 (1.05,1.06)	1.5E-64	1.05 (1.04,1.06)	9.7E-41	1.03 (1.02,1.04)	5.2E-19	1.03 (1.03,1.04)	3.2E-25	1.04 (1.04,1.05)	1.0E-35	1.03 (1.03,1.04)	2.3E-21
digestive	523.31	Acute periodontitis	282062	17450	1.05 (1.05,1.06)	3.9E-41	1.04 (1.03,1.05)	1.4E-23	1.03 (1.02,1.03)	3.6E-11	1.03 (1.02,1.04)	3.5E-15	1.04 (1.03,1.05)	8.3E-22	1.03 (1.02,1.04)	8.2E-13
digestive	523.32	Chronic periodontitis	287968	14089	1.06 (1.05,1.06)	5.4E-36	1.05 (1.04,1.06)	2.4E-22	1.03 (1.02,1.04)	2.6E-11	1.03 (1.03,1.04)	3.5E-15	1.04 (1.03,1.05)	5.7E-20	1.03 (1.02,1.04)	2.4E-12
digestive	525	Other diseases of the teeth and supporting structures	230287	60680	1.06 (1.05,1.06)	3.4E-135	1.05 (1.04,1.05)	1.5E-83	1.03 (1.03,1.03)	6.7E-36	1.04 (1.03,1.04)	3.7E-48	1.04 (1.04,1.05)	4.2E-74	1.03 (1.03,1.04)	4.2E-42
digestive	525.1	Loss of teeth or edentulism	261829	38073	1.05 (1.05,1.06)	7.4E-85	1.05 (1.04,1.05)	1.1E-52	1.03 (1.02,1.03)	4.1E-20	1.03 (1.03,1.04)	1.0E-27	1.04 (1.03,1.05)	5.7E-44	1.03 (1.02,1.03)	7.4E-23
digestive	530	Diseases of esophagus	153615	131627	1.04 (1.04,1.04)	2.3E-102	1.04 (1.03,1.04)	6.2E-85	1.02 (1.02,1.03)	3.2E-31	1.03 (1.03,1.03)	4.7E-54	1.03 (1.03,1.04)	6.7E-68	1.02 (1.02,1.03)	3.9E-30
digestive	530.1	Esophagitis, GERD and related diseases	156612	128556	1.04 (1.04,1.05)	3.8E-102	1.04 (1.03,1.04)	2.2E-84	1.02 (1.02,1.03)	1.6E-31	1.03 (1.03,1.03)	4.4E-54	1.03 (1.03,1.04)	3.5E-67	1.02 (1.02,1.03)	3.4E-30
digestive	530.11	GERD	162469	122039	1.04 (1.04,1.05)	5.1E-101	1.04 (1.03,1.04)	7.5E-84	1.02 (1.02,1.03)	1.6E-31	1.03 (1.03,1.03)	1.9E-53	1.03 (1.03,1.04)	1.5E-65	1.02 (1.02,1.03)	5.9E-30
digestive	532	Dysphagia	252740	40103	1.04 (1.03,1.04)	1.4E-44	1.03 (1.03,1.04)	1.4E-31	1.02 (1.01,1.02)	3.4E-10	1.02 (1.01,1.03)	3.3E-13	1.03 (1.02,1.03)	3.6E-26	1.02 (1.01,1.02)	2.1E-10
digestive	550	Abdominal hernia	237547	50050	1.03 (1.02,1.03)	6.0E-29	1.02 (1.02,1.03)	6.0E-22	1.02 (1.01,1.02)	2.3E-10	1.02 (1.01,1.02)	6.1E-16	1.02 (1.02,1.03)	2.1E-20	1.02 (1.01,1.02)	4.7E-11
digestive	561	Symptoms involving digestive system	233209	41830	1.05 (1.04,1.05)	4.7E-66	1.04 (1.04,1.05)	3.5E-48	1.02 (1.02,1.03)	4.7E-16	1.03 (1.02,1.03)	9.3E-26	1.03 (1.03,1.04)	2.1E-37	1.02 (1.02,1.03)	7.6E-18
digestive	578	Gastrointestinal hemorrhage	250313	35111	1.04 (1.03,1.04)	9.4E-40	1.03 (1.03,1.04)	2.9E-29	1.02 (1.01,1.03)	4.4E-13	1.02 (1.02,1.03)	5.6E-17	1.03 (1.02,1.04)	2.4E-25	1.02 (1.02,1.03)	2.0E-13
endocrine/ metabolic	276	Disorders of fluid, electrolyte, and acid-base balance	221908	54151	1.04 (1.03,1.04)	2.1E-59	1.03 (1.02,1.04)	1.6E-31	1.02 (1.01,1.02)	1.2E-13	1.02 (1.01,1.02)	9.6E-15	1.03 (1.03,1.04)	1.6E-35	1.02 (1.01,1.02)	4.5E-15
endocrine/ metabolic	276.1	Electrolyte imbalance	242364	39794	1.03 (1.03,1.04)	7.2E-33	1.02 (1.02,1.03)	3.2E-15	1.01 (1.01,1.02)	2.4E-07	1.01 (1.01,1.02)	3.6E-07	1.02 (1.02,1.03)	4.2E-19	1.01 (1.01,1.02)	4.3E-08
endocrine/ metabolic	276.5	Hypovolemia	270652	16482	1.05 (1.04,1.05)	1.2E-29	1.04 (1.03,1.05)	4.0E-18	1.02 (1.01,1.03)	1.5E-06	1.02 (1.01,1.03)	4.6E-07	1.03 (1.03,1.04)	1.9E-17	1.02 (1.01,1.03)	1.6E-07
Genito-urinary	591	Urinary tract infection	252052	33029	1.04 (1.04,1.05)	2.2E-42	1.04 (1.03,1.04)	1.4E-28	1.02 (1.02,1.03)	1.2E-12	1.02 (1.02,1.03)	4.0E-16	1.03 (1.03,1.04)	5.0E-27	1.02 (1.02,1.03)	1.6E-13
Genito-urinary	599	Other symptoms/disorders or the urinary system	215307	59968	1.04 (1.04,1.04)	1.6E-63	1.04 (1.03,1.04)	3.6E-47	1.02 (1.02,1.03)	1.7E-17	1.03 (1.02,1.03)	3.6E-32	1.03 (1.03,1.04)	1.1E-39	1.02 (1.02,1.03)	1.1E-19
Genito-urinary	600	Hyperplasia of prostate	189050	97473	1.03 (1.03,1.04)	8.4E-48	1.03 (1.02,1.03)	6.4E-39	1.02 (1.01,1.02)	1.6E-16	1.02 (1.02,1.03)	7.6E-28	1.03 (1.02,1.03)	2.1E-33	1.02 (1.01,1.02)	3.5E-17
Genito-urinary	601	Inflammatory diseases of prostate	281612	14536	1.05 (1.04,1.06)	1.6E-31	1.05 (1.04,1.06)	3.9E-24	1.03 (1.03,1.04)	1.2E-15	1.04 (1.03,1.05)	1.9E-20	1.04 (1.03,1.05)	8.9E-23	1.03 (1.03,1.04)	2.0E-15
Genito-urinary	605	Erectile dysfunction [ED]	209855	76830	1.02 (1.02,1.03)	5.0E-30	1.02 (1.02,1.03)	1.3E-26	1.01 (1.01,1.02)	1.6E-07	1.02 (1.01,1.02)	2.6E-18	1.02 (1.01,1.02)	6.1E-19	1.01 (1.01,1.02)	6.7E-08
infectious diseases	70.3	Viral hepatitis C	294978	13935	1.07 (1.06,1.08)	1.8E-49	1.06 (1.05,1.07)	1.2E-38	1.05 (1.04,1.05)	3.7E-24	1.05 (1.04,1.05)	4.1E-24	1.06 (1.05,1.07)	3.4E-38	1.05 (1.04,1.06)	7.7E-28
infectious diseases	110	Dermatophytosis /	206920	69951	1.03 (1.02,1.03)	2.9E-37	1.02 (1.02,1.03)	7.5E-22	1.01 (1.01,1.02)	8.0E-08	1.02 (1.01,1.02)	2.9E-16	1.02 (1.02,1.03)	2.6E-21	1.01 (1.01,1.02)	1.5E-08

© 2022 Bigdeli TB et al. *JAMA Psychiatry*.

		Dermatomycosis														
infectious diseases	110.1	Dermatophytosis	209524	68178	1.03 (1.02,1.03)	3.1E-36	1.02 (1.02,1.03)	5.6E-21	1.01 (1.01,1.02)	1.7E-07	1.02 (1.01,1.02)	8.9E-16	1.02 (1.02,1.03)	1.1E-20	1.01 (1.01,1.02)	3.3E-08
infectious diseases	110.11	Dermatophytosis of nail	235188	52596	1.03 (1.02,1.03)	2.4E-26	1.02 (1.02,1.03)	1.2E-15	1.01 (1,1.01)	3.4E-05	1.02 (1.01,1.02)	1.8E-11	1.02 (1.01,1.02)	2.8E-15	1.01 (1.01,1.02)	1.0E-05
injuries & poisonings	916	Contusion	261799	18348	1.06 (1.05,1.07)	7.5E-51	1.05 (1.04,1.06)	3.9E-33	1.03 (1.02,1.04)	7.8E-17	1.04 (1.03,1.05)	2.5E-21	1.04 (1.04,1.05)	3.8E-28	1.03 (1.03,1.04)	4.9E-19
injuries & poisonings	969	Poisoning by psychotropic agents	306220	1944	1.14 (1.11,1.17)	4.0E-30	1.14 (1.11,1.18)	1.3E-16	1.1 (1.07,1.12)	4.0E-16	1.07 (1.05,1.1)	1.5E-09	1.1 (1.07,1.12)	1.7E-15	1.11 (1.08,1.13)	2.9E-18
mental disorders	290.1	Dementias	291550	14058	1.05 (1.04,1.06)	1.2E-29	1.04 (1.03,1.05)	4.2E-15	1.02 (1.01,1.03)	2.4E-05	1.02 (1.01,1.03)	4.5E-07	1.04 (1.03,1.05)	1.0E-17	1.02 (1.01,1.03)	1.3E-07
mental disorders	290.3	Other persistent mental disorders due to conditions classified elsewhere	288225	14474	1.06 (1.05,1.06)	1.7E-37	1.04 (1.03,1.05)	9.1E-19	1.02 (1.01,1.03)	2.6E-05	1.02 (1.01,1.03)	9.6E-08	1.03 (1.03,1.04)	3.4E-15	1.03 (1.02,1.03)	5.4E-09
mental disorders	292	Neurological disorders	246745	40721	1.05 (1.05,1.06)	9.6E-88	1.05 (1.04,1.05)	9.9E-52	1.02 (1.02,1.03)	1.8E-16	1.03 (1.02,1.03)	6.5E-24	1.04 (1.03,1.04)	3.1E-43	1.03 (1.02,1.03)	3.8E-24
mental disorders	292.4	Altered mental status	288556	10297	1.07 (1.06,1.08)	6.4E-44	1.05 (1.04,1.07)	6.7E-19	1.04 (1.03,1.05)	2.4E-13	1.03 (1.02,1.04)	8.5E-09	1.05 (1.04,1.06)	4.6E-24	1.04 (1.03,1.05)	4.8E-17
mental disorders	296	Mood disorders	161370	132442	1.11 (1.1,1.11)	<1E-300	1.1 (1.09,1.1)	<1E-300	1.04 (1.02,1.06)	1.1E-04	1.09 (1.08,1.09)	<1E-300	1.09 (1.09,1.1)	<1E-300	1.08 (1.07,1.08)	1.7E-174
mental disorders	296.1	Bipolar	285708	20399	1.16 (1.15,1.17)	<1E-300	1.14 (1.13,1.15)	3.0E-174	1.11 (1.1,1.12)	3.2E-166	1.1 (1.09,1.11)	5.6E-121	1.12 (1.11,1.13)	1.5E-157	1.13 (1.12,1.14)	5.9E-219
mental disorders	296.2	Depression	167868	124718	1.1 (1.09,1.1)	<1E-300	1.09 (1.09,1.1)	<1E-300	1 (0.99,1.01)	5.6E-01	1.08 (1.07,1.08)	1.5E-290	1.09 (1.08,1.09)	<1E-300	1.06 (1.06,1.07)	2.2E-126
mental disorders	296.22	Major depressive disorder	200712	92217	1.09 (1.09,1.1)	<1E-300	1.09 (1.08,1.09)	2.3E-299	1.02 (1.02,1.03)	4.1E-16	1.07 (1.06,1.07)	1.2E-196	1.08 (1.07,1.08)	8.8E-261	1.05 (1.05,1.06)	2.3E-90
mental disorders	297	Suicidal ideation or attempt	285894	14832	1.13 (1.12,1.14)	7.6E-166	1.1 (1.09,1.12)	1.2E-77	1.08 (1.07,1.09)	2.4E-73	1.07 (1.06,1.08)	5.0E-53	1.09 (1.09,1.1)	8.1E-91	1.09 (1.08,1.1)	6.1E-90
mental disorders	297.1	Suicidal ideation	291719	12317	1.13 (1.12,1.14)	1.6E-149	1.11 (1.1,1.12)	5.3E-71	1.09 (1.08,1.1)	3.5E-67	1.07 (1.06,1.09)	6.2E-47	1.1 (1.09,1.11)	6.5E-82	1.1 (1.09,1.11)	1.1E-81
mental disorders	297.2	Suicide or self-inflicted injury	304207	3948	1.12 (1.11,1.14)	1.3E-47	1.11 (1.09,1.13)	9.2E-22	1.08 (1.07,1.1)	2.1E-22	1.06 (1.04,1.08)	2.3E-12	1.08 (1.07,1.1)	1.3E-22	1.09 (1.07,1.11)	8.7E-27
mental disorders	300	Anxiety disorders	166912	123000	1.09 (1.09,1.1)	<1E-300	1.09 (1.08,1.09)	<1E-300	1.05 (1.04,1.05)	7.2E-80	1.07 (1.07,1.08)	4.7E-236	1.08 (1.08,1.08)	1.2E-299	1.06 (1.05,1.06)	4.8E-122
mental disorders	300.1	Anxiety disorder	206217	79142	1.09 (1.09,1.1)	<1E-300	1.09 (1.08,1.09)	1.1E-279	1.05 (1.05,1.06)	1.7E-102	1.07 (1.07,1.08)	2.0E-201	1.08 (1.08,1.09)	7.2E-258	1.06 (1.06,1.07)	3.4E-130
mental disorders	300.11	Generalized anxiety disorder	278052	21853	1.08 (1.07,1.09)	4.1E-99	1.08 (1.07,1.08)	9.1E-78	1.04 (1.03,1.05)	3.4E-26	1.05 (1.04,1.06)	1.1E-43	1.06 (1.05,1.07)	4.4E-63	1.04 (1.04,1.05)	2.0E-32
mental disorders	300.12	Agorophobia, social phobia, and panic disorder	292966	12188	1.08 (1.07,1.09)	2.1E-67	1.08 (1.07,1.09)	1.2E-50	1.05 (1.04,1.06)	8.3E-23	1.05 (1.04,1.06)	2.5E-26	1.07 (1.06,1.08)	4.5E-41	1.05 (1.04,1.06)	1.1E-25
mental disorders	300.3	Obsessive-compulsive disorders	306240	3142	1.11 (1.09,1.13)	2.2E-32	1.1 (1.08,1.13)	1.2E-20	1.07 (1.05,1.09)	9.2E-15	1.07 (1.05,1.09)	5.4E-14	1.09 (1.07,1.11)	9.2E-21	1.08 (1.06,1.1)	6.8E-17
mental disorders	300.4	Dysthymic disorder	274401	23556	1.08 (1.07,1.09)	2.9E-117	1.08 (1.07,1.08)	5.0E-85	1.03 (1.03,1.04)	6.8E-22	1.05 (1.05,1.06)	5.3E-51	1.06 (1.06,1.07)	1.4E-72	1.04 (1.04,1.05)	1.3E-34
mental disorders	300.9	Posttraumatic stress disorder	230809	69537	1.06 (1.06,1.07)	1.4E-166	1.06 (1.05,1.06)	5.1E-117	1.02 (1.02,1.03)	6.9E-18	1.04 (1.03,1.04)	6.3E-55	1.05 (1.04,1.05)	3.2E-90	1.03 (1.02,1.03)	2.4E-32
mental disorders	301	Personality disorders	290676	13909	1.14 (1.12,1.15)	2.2E-177	1.12 (1.11,1.13)	7.9E-93	1.09 (1.08,1.1)	8.0E-85	1.08 (1.07,1.09)	1.6E-65	1.1 (1.09,1.11)	2.2E-101	1.1 (1.09,1.11)	7.2E-104
mental disorders	301.2	Antisocial/borderline	303440	5191	1.15 (1.14,1.17)	9.3E-85	1.14 (1.12,1.16)	2.8E-44	1.11 (1.09,1.13)	1.5E-46	1.09 (1.07,1.1)	3.1E-29	1.11 (1.1,1.13)	2.2E-46	1.12 (1.1,1.14)	6.2E-54

© 2022 Bigdeli TB et al. *JAMA Psychiatry*.

		personality disorder														
mental disorders	303	Psychogenic and somatoform disorders	297603	7203	1.08 (1.06,1.09)	1.8E-34	1.08 (1.06,1.09)	1.0E-26	1.04 (1.03,1.05)	2.1E-10	1.05 (1.03,1.06)	1.3E-13	1.05 (1.04,1.06)	2.1E-15	1.04 (1.03,1.06)	2.0E-12
mental disorders	303.4	Somatoform disorder	300051	6434	1.08 (1.06,1.09)	2.2E-31	1.08 (1.06,1.09)	2.3E-24	1.04 (1.03,1.05)	1.7E-09	1.05 (1.03,1.06)	6.7E-13	1.05 (1.04,1.06)	3.4E-14	1.04 (1.03,1.06)	2.8E-11
mental disorders	304	Adjustment reaction	238220	47151	1.06 (1.05,1.06)	2.5E-108	1.05 (1.05,1.06)	3.1E-83	1.02 (1.01,1.03)	1.6E-13	1.04 (1.04,1.05)	5.7E-55	1.05 (1.04,1.05)	2.1E-71	1.03 (1.02,1.03)	3.0E-27
mental disorders	306	Other mental disorder	257665	23904	1.07 (1.06,1.08)	2.2E-89	1.06 (1.05,1.06)	7.5E-47	1.03 (1.03,1.04)	9.8E-23	1.04 (1.04,1.05)	5.2E-33	1.05 (1.04,1.06)	8.1E-46	1.04 (1.03,1.05)	3.8E-31
mental disorders	316	Substance addiction and disorders	261155	36576	1.1 (1.09,1.1)	2.2E-215	1.08 (1.08,1.09)	6.6E-134	1.06 (1.05,1.07)	1.6E-82	1.06 (1.06,1.07)	6.2E-83	1.08 (1.07,1.08)	2.0E-134	1.07 (1.06,1.07)	6.2E-104
mental disorders	317.1	Alcoholism	255939	39754	1.07 (1.06,1.07)	1.5E-125	1.06 (1.05,1.06)	1.8E-77	1.04 (1.03,1.05)	1.7E-42	1.04 (1.04,1.05)	1.3E-48	1.05 (1.05,1.06)	1.5E-81	1.05 (1.04,1.05)	8.2E-57
mental disorders	318	Tobacco use disorder	175890	103781	1.05 (1.05,1.06)	3.5E-141	1.04 (1.04,1.05)	8.4E-99	1.03 (1.03,1.04)	1.4E-59	1.04 (1.03,1.04)	2.8E-72	1.04 (1.04,1.05)	1.6E-104	1.03 (1.03,1.04)	7.9E-65
Musculo-skeletal	716	Other arthropathies	247336	33669	1.03 (1.03,1.04)	3.7E-27	1.03 (1.03,1.04)	2.5E-24	1.02 (1.01,1.02)	1.9E-07	1.02 (1.02,1.03)	6.4E-14	1.02 (1.02,1.03)	7.2E-16	1.01 (1.01,1.02)	2.0E-06
Muscul-oskeletal	720	Spinal stenosis	265640	30340	1.04 (1.03,1.04)	1.2E-32	1.04 (1.03,1.04)	1.3E-27	1.02 (1.01,1.02)	6.8E-09	1.03 (1.02,1.03)	1.0E-18	1.03 (1.02,1.03)	1.9E-18	1.01 (1.01,1.02)	3.2E-06
Musculo-skeletal	721	Spondylosis and allied disorders	238955	44356	1.04 (1.04,1.05)	7.1E-55	1.04 (1.04,1.05)	1.1E-49	1.02 (1.01,1.03)	1.2E-14	1.03 (1.02,1.04)	1.0E-30	1.03 (1.02,1.04)	6.9E-30	1.02 (1.01,1.02)	3.3E-11
Musculo-skeletal	721.1	Spondylosis without myelopathy	242860	40805	1.04 (1.03,1.05)	7.7E-49	1.04 (1.03,1.05)	9.5E-44	1.02 (1.01,1.02)	1.3E-12	1.03 (1.02,1.03)	7.4E-27	1.03 (1.02,1.03)	5.4E-26	1.02 (1.01,1.02)	1.2E-09
Musculo-skeletal	722	Intervertebral disc disorders	228596	55337	1.04 (1.04,1.04)	7.4E-62	1.04 (1.03,1.04)	3.0E-55	1.02 (1.02,1.03)	3.5E-17	1.03 (1.02,1.03)	6.3E-34	1.03 (1.02,1.03)	4.3E-34	1.02 (1.01,1.02)	5.0E-13
Musculo-skeletal	722.6	Degeneration of intervertebral disc	242906	42226	1.04 (1.03,1.04)	1.6E-48	1.04 (1.03,1.05)	1.6E-44	1.02 (1.01,1.03)	5.1E-14	1.03 (1.02,1.03)	1.1E-26	1.03 (1.02,1.03)	1.4E-26	1.02 (1.01,1.02)	8.0E-11
Musculo-skeletal	740	Osteoarthrosis	146222	131442	1.03 (1.02,1.03)	3.6E-39	1.02 (1.02,1.03)	4.7E-34	1.01 (1,1.01)	3.6E-06	1.02 (1.01,1.02)	1.2E-19	1.02 (1.01,1.02)	1.5E-21	1.01 (1,1.01)	8.8E-05
Musculo-skeletal	740.9	Osteoarthrosis NOS	179236	99593	1.03 (1.03,1.03)	2.9E-51	1.03 (1.02,1.03)	4.1E-42	1.01 (1.01,1.02)	1.5E-11	1.02 (1.02,1.03)	5.5E-26	1.02 (1.02,1.03)	5.7E-30	1.01 (1.01,1.02)	1.6E-09
Musculo-skeletal	745	Pain in joint	89094	187207	1.03 (1.03,1.04)	7.6E-61	1.03 (1.03,1.04)	3.5E-49	1.01 (1.01,1.02)	5.2E-10	1.02 (1.02,1.03)	1.7E-31	1.03 (1.02,1.03)	9.6E-36	1.01 (1.01,1.02)	2.2E-09
Neuro-logical	327	Sleep disorders	191386	81129	1.05 (1.05,1.05)	2.0E-116	1.05 (1.04,1.05)	1.0E-92	1.02 (1.01,1.02)	1.5E-15	1.03 (1.03,1.04)	8.9E-48	1.04 (1.03,1.04)	1.2E-62	1.02 (1.01,1.02)	6.8E-18
Neuro-logical	327.3	Sleep apnea	195189	101623	1.02 (1.02,1.03)	1.8E-30	1.02 (1.02,1.03)	2.4E-29	1 (1,1.01)	1.3E-01	1.02 (1.01,1.02)	1.5E-15	1.01 (1.01,1.02)	3.2E-14	1 (1,1.01)	4.0E-02
Neuro-logical	327.4	Insomnia	230277	51649	1.05 (1.05,1.06)	1.1E-96	1.05 (1.04,1.05)	3.6E-75	1.02 (1.02,1.03)	6.2E-17	1.03 (1.03,1.04)	1.8E-37	1.04 (1.03,1.04)	5.0E-55	1.02 (1.02,1.03)	2.0E-19
Neuro-logical	338	Pain	225658	47039	1.06 (1.05,1.06)	8.4E-104	1.05 (1.05,1.06)	3.6E-79	1.03 (1.02,1.03)	3.5E-28	1.04 (1.03,1.04)	1.8E-46	1.04 (1.04,1.05)	1.7E-58	1.03 (1.02,1.03)	4.4E-28
Neuro-logical	338.2	Chronic pain	248178	34632	1.06 (1.06,1.07)	3.6E-99	1.06 (1.05,1.06)	4.0E-74	1.03 (1.03,1.04)	3.2E-30	1.04 (1.04,1.05)	2.3E-47	1.05 (1.04,1.05)	8.9E-57	1.03 (1.03,1.04)	2.4E-30
Neuro-logical	339	Other headache syndromes	237942	42426	1.05 (1.04,1.05)	8.1E-67	1.04 (1.04,1.05)	1.6E-47	1.02 (1.02,1.03)	8.6E-14	1.03 (1.02,1.03)	9.2E-25	1.03 (1.02,1.03)	8.1E-19	1.02 (1.01,1.03)	2.1E-12
Neuro-logical	350	Abnormal movement	220347	57430	1.04 (1.04,1.05)	2.1E-68	1.04 (1.03,1.04)	2.8E-43	1.02 (1.01,1.02)	3.9E-10	1.02 (1.02,1.03)	2.6E-21	1.03 (1.02,1.03)	1.8E-31	1.02 (1.01,1.02)	3.9E-12
Neuro-logical	350.2	Abnormality of gait	238406	44292	1.04 (1.04,1.05)	6.7E-53	1.03 (1.03,1.04)	1.3E-33	1.01 (1.01,1.02)	2.2E-08	1.02 (1.02,1.03)	3.6E-17	1.03 (1.02,1.03)	5.4E-27	1.02 (1.01,1.02)	1.2E-09
Neuro-logical	351	Other peripheral nerve disorders	227740	57720	1.03 (1.02,1.03)	2.3E-36	1.03 (1.02,1.03)	2.2E-32	1.01 (1.01,1.02)	6.4E-06	1.02 (1.02,1.02)	1.2E-17	1.02 (1.01,1.02)	1.7E-16	1.01 (1,1.01)	2.8E-03

© 2022 Bigdeli TB et al. *JAMA Psychiatry*.

Neuro-logical	355.1	Chronic pain syndrome	283361	17187	1.07 (1.06,1.08)	8.8E-64	1.07 (1.06,1.08)	3.6E-54	1.04 (1.03,1.05)	8.4E-21	1.05 (1.04,1.06)	1.7E-32	1.05 (1.04,1.06)	2.0E-34	1.04 (1.03,1.05)	5.8E-20
Neuro-logical	356	Hereditary and idiopathic peripheral neuropathy	264091	26984	1.03 (1.03,1.04)	3.8E-26	1.03 (1.03,1.04)	4.7E-22	1.01 (1.01,1.02)	2.2E-05	1.02 (1.02,1.03)	1.8E-12	1.02 (1.02,1.03)	4.5E-12	1.01 (1,1.01)	8.7E-03
respiratory	464	Acute sinusitis	264174	17840	1.06 (1.06,1.07)	1.2E-57	1.06 (1.06,1.07)	9.9E-51	1.05 (1.04,1.05)	3.8E-30	1.05 (1.05,1.06)	4.7E-40	1.05 (1.04,1.06)	1.7E-39	1.05 (1.04,1.05)	8.0E-30
respiratory	465	Acute upper respiratory infections of multiple or unspecified sites	204993	53253	1.06 (1.05,1.06)	9.3E-121	1.05 (1.05,1.06)	2.0E-85	1.04 (1.03,1.04)	3.2E-49	1.04 (1.04,1.05)	4.6E-67	1.05 (1.04,1.05)	2.1E-78	1.04 (1.03,1.04)	1.2E-51
respiratory	465.2	Acute pharyngitis	276813	9789	1.06 (1.05,1.08)	2.9E-33	1.06 (1.05,1.07)	2.5E-24	1.05 (1.03,1.06)	1.8E-17	1.05 (1.04,1.06)	1.4E-20	1.05 (1.04,1.06)	1.1E-22	1.05 (1.04,1.06)	2.3E-18
respiratory	475	Chronic sinusitis	257693	26585	1.04 (1.04,1.05)	4.7E-42	1.04 (1.03,1.05)	1.6E-33	1.03 (1.02,1.03)	1.3E-17	1.04 (1.03,1.04)	1.0E-26	1.03 (1.03,1.04)	3.5E-26	1.03 (1.02,1.03)	1.0E-16
respiratory	476	Allergic rhinitis	215075	63297	1.03 (1.02,1.03)	1.9E-36	1.03 (1.02,1.03)	1.3E-30	1.02 (1.01,1.02)	4.3E-14	1.02 (1.02,1.03)	5.2E-25	1.02 (1.02,1.03)	5.6E-24	1.02 (1.01,1.02)	3.8E-13
respiratory	480	Pneumonia	260383	31337	1.04 (1.03,1.05)	3.4E-39	1.03 (1.03,1.04)	1.9E-26	1.02 (1.01,1.02)	4.0E-10	1.02 (1.01,1.03)	2.0E-11	1.03 (1.03,1.04)	2.0E-25	1.02 (1.01,1.03)	1.8E-10
respiratory	496	Chronic airway obstruction	215720	72353	1.06 (1.05,1.06)	9.2E-137	1.05 (1.05,1.06)	4.1E-107	1.04 (1.03,1.04)	3.9E-62	1.04 (1.04,1.05)	2.8E-79	1.05 (1.04,1.05)	1.9E-105	1.04 (1.03,1.04)	3.1E-63
respiratory	496.2	Chronic bronchitis	276924	19677	1.06 (1.05,1.07)	4.3E-59	1.06 (1.05,1.06)	5.7E-43	1.04 (1.03,1.05)	2.6E-26	1.04 (1.04,1.05)	3.9E-31	1.05 (1.05,1.06)	6.8E-45	1.04 (1.03,1.05)	3.0E-27
respiratory	496.21	Obstructive chronic bronchitis	282188	17154	1.06 (1.05,1.07)	2.0E-50	1.06 (1.05,1.06)	4.3E-37	1.04 (1.03,1.05)	8.0E-22	1.04 (1.03,1.05)	8.8E-26	1.05 (1.04,1.06)	2.3E-38	1.04 (1.03,1.05)	1.1E-22
respiratory	497	Bronchitis	268282	14750	1.07 (1.06,1.08)	7.7E-60	1.07 (1.06,1.08)	1.4E-45	1.05 (1.04,1.06)	9.3E-29	1.05 (1.04,1.06)	2.0E-34	1.06 (1.05,1.07)	4.1E-42	1.05 (1.04,1.06)	3.5E-30
respiratory	509	Respiratory failure, insufficiency, arrest	275454	22821	1.04 (1.03,1.05)	1.2E-28	1.04 (1.03,1.04)	7.6E-22	1.02 (1.01,1.03)	2.3E-08	1.02 (1.01,1.03)	1.5E-07	1.03 (1.02,1.04)	4.6E-20	1.02 (1.01,1.03)	3.3E-08
respiratory	512	Other symptoms of respiratory system	139662	120187	1.04 (1.04,1.05)	1.6E-104	1.04 (1.03,1.04)	3.1E-78	1.02 (1.02,1.02)	4.8E-24	1.03 (1.03,1.03)	3.0E-49	1.03 (1.03,1.04)	5.1E-66	1.02 (1.02,1.03)	2.3E-25
respiratory	512.7	Shortness of breath	209689	60907	1.04 (1.04,1.05)	3.7E-70	1.04 (1.03,1.04)	9.6E-53	1.02 (1.02,1.03)	3.5E-18	1.03 (1.02,1.03)	7.1E-33	1.03 (1.03,1.04)	1.2E-46	1.02 (1.02,1.03)	1.8E-18
respiratory	512.8	Cough	212486	48953	1.03 (1.03,1.04)	2.6E-35	1.03 (1.02,1.03)	2.4E-22	1.01 (1.01,1.02)	4.7E-07	1.02 (1.01,1.02)	1.1E-14	1.02 (1.02,1.03)	1.3E-19	1.01 (1.01,1.02)	1.0E-07
respiratory	512.9	Other dyspnea	219490	48646	1.03 (1.02,1.03)	9.6E-32	1.03 (1.02,1.03)	1.0E-25	1.01 (1,1.01)	1.5E-04	1.02 (1.01,1.02)	1.1E-12	1.02 (1.02,1.03)	3.4E-18	1.01 (1,1.01)	9.1E-05
sense organs	366	Cataract	116658	166416	1.03 (1.02,1.03)	2.1E-37	1.02 (1.02,1.03)	1.9E-28	1.01 (1.01,1.01)	1.4E-06	1.02 (1.01,1.02)	2.0E-18	1.02 (1.02,1.02)	2.3E-24	1.01 (1.01,1.01)	1.7E-06
sense organs	366.2	Senile cataract	139596	142445	1.02 (1.02,1.03)	5.4E-28	1.02 (1.01,1.02)	1.1E-20	1.01 (1,1.01)	1.5E-04	1.01 (1.01,1.02)	3.0E-13	1.02 (1.01,1.02)	5.2E-18	1.01 (1,1.01)	1.2E-04
sense organs	367	Disorders of refraction and accommodation; blindness and low vision	86906	200705	1.03 (1.03,1.04)	5.2E-58	1.03 (1.02,1.03)	5.4E-43	1.01 (1.01,1.02)	3.5E-11	1.02 (1.02,1.03)	4.4E-27	1.03 (1.02,1.03)	4.6E-34	1.01 (1.01,1.02)	1.0E-11
sense organs	375.1	Dry eyes	216725	58461	1.03 (1.02,1.03)	9.9E-29	1.02 (1.02,1.03)	1.1E-23	1.01 (1.01,1.02)	3.5E-06	1.02 (1.01,1.02)	1.7E-14	1.02 (1.01,1.02)	5.0E-16	1.01 (1.01,1.01)	1.8E-05
sense organs	386.9	Dizziness and giddiness (Light-headedness and vertigo)	231860	43001	1.04 (1.04,1.05)	2.2E-56	1.04 (1.03,1.04)	7.8E-41	1.02 (1.01,1.02)	1.7E-12	1.03 (1.02,1.03)	9.3E-27	1.03 (1.03,1.04)	6.7E-31	1.02 (1.02,1.03)	2.4E-14
symptoms	760	Back pain	128684	149486	1.05 (1.05,1.05)	2.3E-146	1.05 (1.04,1.05)	2.6E-121	1.03 (1.02,1.03)	7.0E-46	1.04 (1.04,1.04)	2.5E-86	1.04 (1.04,1.05)	2.7E-96	1.03 (1.02,1.03)	4.1E-41

© 2022 Bigdeli TB et al. *JAMA Psychiatry*.

symptoms	761	Cervicalgia	226921	56206	1.05 (1.05,1.06)	3.9E-96	1.05 (1.04,1.05)	2.8E-78	1.03 (1.02,1.03)	1.9E-33	1.04 (1.03,1.04)	4.2E-56	1.04 (1.03,1.04)	4.5E-56	1.03 (1.02,1.03)	9.6E-31
symptoms	763	Thoracic or lumbosacral neuritis or radiculitis, unspecified	263827	29935	1.04 (1.03,1.05)	2.5E-38	1.04 (1.03,1.05)	2.6E-36	1.02 (1.01,1.03)	1.2E-11	1.03 (1.02,1.04)	9.6E-22	1.03 (1.02,1.04)	1.2E-20	1.02 (1.01,1.02)	5.3E-08
symptoms	770	Myalgia and myositis unspecified	273265	19134	1.06 (1.05,1.06)	5.4E-48	1.05 (1.04,1.06)	9.9E-37	1.03 (1.02,1.04)	1.3E-16	1.04 (1.03,1.05)	3.2E-25	1.04 (1.03,1.05)	1.6E-26	1.03 (1.02,1.04)	1.6E-15
symptoms	772.3	Muscle weakness	265118	25069	1.04 (1.03,1.05)	6.0E-35	1.04 (1.03,1.04)	5.0E-23	1.02 (1.01,1.02)	5.1E-07	1.02 (1.01,1.03)	2.4E-10	1.03 (1.02,1.04)	1.1E-19	1.02 (1.01,1.02)	9.2E-08
symptoms	785	Abdominal pain	206085	63494	1.05 (1.05,1.06)	4.1E-120	1.05 (1.05,1.06)	9.0E-94	1.03 (1.03,1.04)	4.0E-42	1.04 (1.03,1.04)	1.7E-60	1.04 (1.04,1.05)	4.3E-77	1.03 (1.03,1.04)	5.3E-42
symptoms	788	Syncope and collapse	267138	25048	1.04 (1.03,1.04)	7.3E-28	1.03 (1.02,1.04)	9.6E-19	1.01 (1.01,1.02)	1.7E-04	1.02 (1.01,1.03)	2.6E-08	1.02 (1.02,1.03)	4.0E-13	1.01 (1.01,1.02)	2.0E-05
symptoms	789	Nausea and vomiting	262919	21357	1.06 (1.05,1.07)	9.3E-56	1.05 (1.04,1.06)	8.1E-36	1.03 (1.02,1.04)	7.8E-18	1.03 (1.02,1.04)	1.6E-18	1.04 (1.04,1.05)	5.1E-32	1.03 (1.03,1.04)	5.3E-19
symptoms	798	Malaise and fatigue	223258	47019	1.04 (1.04,1.05)	7.8E-65	1.04 (1.03,1.05)	3.7E-47	1.02 (1.01,1.02)	1.7E-11	1.03 (1.02,1.03)	2.2E-26	1.03 (1.03,1.04)	1.4E-39	1.02 (1.01,1.02)	6.1E-14

© 2022 Bigdeli TB et al. *JAMA Psychiatry*.

eTable 30. Significant findings ($p<10^{-5}$) in PheWAS of common factor PRS in AA participants.

Results are grouped by disease category, and numbers of controls and cases are displayed. Effect sizes and significance are reported for six models: (i) the primary analysis, adjusting for sex, age, age^2, and ancestry PCs; (ii) following exclusion individuals with psychotic or mood disorders; (iii) adjusting for any diagnosis of a psychotic or mood-disorder (separately); or (iv) adjusting for lifetime treatment with antipsychotics; (v) adjusting for lifetime treatment with mood stabilizers; (vi) adjusting for lifetime treatment with antidepressants. Odds ratios are per SD unit increase in PRS.

Group	Phecode	Description	Cont	Case	Base		Exclude phecode 295*		Phecode-adjusted		Antipsychotic-adjusted		Mood stabilizer-adjusted		Antidepressant-adjusted	
					OR (95% CI)	*p*-value	OR (95% CI)	*p*-value	OR (95% CI)	*p*-value	OR (95% CI)	*p*-value	OR (95% CI)	*p*-value	OR (95% CI)	*p*-value
circulatory system	418	Nonspecific chest pain	41653	29692	1.02 (1.02,1.03)	2.8E-16	1.02 (1.01,1.03)	1.0E-11	1.02 (1.01,1.02)	4.2E-09	1.02 (1.01,1.02)	9.5E-11	1.02 (1.01,1.02)	5.5E-12	1.02 (1.01,1.02)	2.5E-10
dermato-logic	681	Superficial cellulitis and abscess	59215	13907	1.02 (1.01,1.03)	4.2E-08	1.01 (1.01,1.02)	1.7E-04	1.01 (1.01,1.02)	3.2E-05	1.01 (1.01,1.02)	4.3E-05	1.02 (1.01,1.02)	3.2E-06	1.01 (1.01,1.02)	1.2E-05
digestive	521	Diseases of hard tissues of teeth	48819	27048	1.02 (1.02,1.03)	7.3E-15	1.02 (1.01,1.02)	7.5E-08	1.01 (1.01,1.02)	1.1E-06	1.01 (1.01,1.02)	3.2E-07	1.02 (1.01,1.02)	1.7E-09	1.02 (1.01,1.02)	1.2E-08
digestive	521.1	Dental caries	49444	26480	1.02 (1.02,1.03)	6.7E-14	1.02 (1.01,1.02)	3.8E-07	1.01 (1.01,1.02)	4.1E-06	1.01 (1.01,1.02)	1.2E-06	1.02 (1.01,1.02)	8.7E-09	1.02 (1.01,1.02)	4.7E-08
digestive	523	Gingival and periodontal diseases	53599	22773	1.02 (1.01,1.02)	3.1E-11	1.01 (1.01,1.02)	3.8E-06	1.01 (1.01,1.02)	9.2E-05	1.01 (1.01,1.02)	2.4E-05	1.01 (1.01,1.02)	6.3E-07	1.01 (1.01,1.02)	2.8E-06
digestive	523.3	Periodontitis (acute or chronic)	63670	12964	1.02 (1.01,1.02)	3.5E-06	1.01 (1,1.02)	2.0E-03	1.01 (1,1.02)	1.3E-02	1.01 (1,1.02)	7.1E-03	1.01 (1,1.02)	7.3E-04	1.01 (1,1.02)	1.9E-03
digestive	525	Other diseases of the teeth and supporting structures	47747	27265	1.02 (1.01,1.03)	2.4E-13	1.01 (1.01,1.02)	8.4E-06	1.01 (1.01,1.02)	2.7E-05	1.01 (1.01,1.02)	7.8E-06	1.02 (1.01,1.02)	2.9E-08	1.01 (1.01,1.02)	2.7E-07
digestive	525.1	Loss of teeth or edentulism	62014	16377	1.02 (1.01,1.03)	3.9E-10	1.01 (1.01,1.02)	1.7E-04	1.01 (1.01,1.02)	2.1E-04	1.01 (1.01,1.02)	1.3E-04	1.02 (1.01,1.02)	1.7E-06	1.01 (1.01,1.02)	1.5E-05
digestive	530	Diseases of esophagus	42841	32106	1.01 (1.01,1.02)	3.4E-08	1.01 (1.01,1.02)	1.4E-06	1.01 (1,1.01)	7.7E-04	1.01 (1,1.02)	1.1E-04	1.01 (1.01,1.02)	1.7E-05	1.01 (1,1.01)	4.6E-04
digestive	530.1	Esophagitis, GERD and related diseases	43610	31338	1.01 (1.01,1.02)	2.9E-08	1.01 (1.01,1.02)	1.3E-06	1.01 (1,1.01)	7.0E-04	1.01 (1,1.02)	9.0E-05	1.01 (1.01,1.02)	1.6E-05	1.01 (1,1.02)	3.8E-04
digestive	530.11	GERD	44780	30147	1.01 (1.01,1.02)	5.2E-08	1.01 (1.01,1.02)	4.2E-06	1.01 (1,1.01)	8.7E-04	1.01 (1,1.02)	1.3E-04	1.01 (1.01,1.02)	2.8E-05	1.01 (1,1.01)	5.8E-04
digestive	561	Symptoms involving digestive system	61913	10387	1.02 (1.02,1.03)	9.7E-10	1.02 (1.01,1.03)	1.4E-07	1.02 (1.01,1.03)	6.7E-06	1.02 (1.01,1.03)	3.2E-06	1.02 (1.01,1.03)	3.7E-07	1.02 (1.01,1.03)	1.3E-06
endocrine/ metabolic	276	Disorders of fluid, electrolyte, and acid-base balance	55412	17337	1.02 (1.01,1.02)	8.6E-07	1.01 (1,1.02)	3.1E-03	1.01 (1,1.02)	3.2E-04	1.01 (1,1.02)	8.9E-04	1.01 (1.01,1.02)	4.3E-05	1.01 (1.01,1.02)	1.2E-04
endocrine/ metabolic	276.5	Hypovolemia	70215	5188	1.03 (1.02,1.04)	1.8E-07	1.02 (1.01,1.03)	2.8E-04	1.02 (1.01,1.03)	2.7E-05	1.02 (1.01,1.03)	7.6E-05	1.02 (1.01,1.04)	4.3E-06	1.02 (1.01,1.03)	1.3E-05
infectious diseases	110	Dermatophytosis / Dermatomycosis	45593	27229	1.02 (1.01,1.02)	4.9E-09	1.01 (1.01,1.02)	2.3E-06	1.01 (1.01,1.02)	4.0E-05	1.01 (1.01,1.02)	7.5E-06	1.01 (1.01,1.02)	1.4E-06	1.01 (1.01,1.02)	8.2E-06
infectious diseases	110.1	Dermatophytosis	46880	26148	1.02 (1.01,1.02)	7.7E-09	1.01 (1.01,1.02)	2.0E-06	1.01 (1.01,1.02)	4.6E-05	1.01 (1.01,1.02)	1.0E-05	1.01 (1.01,1.02)	2.1E-06	1.01 (1.01,1.02)	9.4E-06
infectious diseases	110.11	Dermatophytosis of nail	55927	19673	1.01 (1.01,1.02)	9.0E-06	1.01 (1.01,1.02)	1.2E-04	1.01 (1,1.02)	3.0E-03	1.01 (1,1.02)	1.4E-03	1.01 (1,1.02)	4.4E-04	1.01 (1,1.02)	8.3E-04
mental disorders	292	Neurological disorders	66397	10268	1.02 (1.01,1.03)	1.4E-06	1.01 (1,1.02)	5.8E-03	1.01 (1,1.02)	8.3E-03	1.01 (1,1.02)	1.2E-02	1.01 (1,1.02)	9.6E-04	1.01 (1,1.02)	1.4E-03
mental disorders	296	Mood disorders	33552	44186	1.03 (1.03,1.04)	2.0E-29	1.03 (1.02,1.03)	8.3E-19	0.99 (0.97,1.01)	2.5E-01	1.02 (1.02,1.03)	4.8E-15	1.03 (1.02,1.03)	7.3E-20	1.02 (1.02,1.03)	6.5E-12
mental disorders	296.1	Bipolar	75000	5921	1.04 (1.03,1.06)	3.2E-19	1.04 (1.03,1.06)	1.5E-09	1.03 (1.02,1.04)	3.4E-11	1.03 (1.02,1.04)	3.2E-07	1.03 (1.02,1.04)	1.3E-08	1.04 (1.03,1.05)	2.2E-14
mental disorders	296.2	Depression	35260	42196	1.03 (1.02,1.03)	3.0E-26	1.02 (1.02,1.03)	2.2E-17	0.99 (0.98,1.01)	2.6E-01	1.02 (1.02,1.03)	1.2E-13	1.02 (1.02,1.03)	3.4E-18	1.02 (1.01,1.03)	1.1E-09
mental disorders	296.22	Major depressive disorder	44614	32643	1.02 (1.02,1.03)	2.8E-17	1.02 (1.01,1.03)	2.9E-11	1 (0.99,1.01)	8.1E-01	1.01 (1.01,1.02)	2.0E-07	1.02 (1.01,1.02)	8.9E-11	1.01 (1.01,1.02)	7.1E-05
mental disorders	297	Suicidal ideation or attempt	72688	6386	1.04 (1.03,1.05)	5.4E-14	1.03 (1.02,1.05)	9.4E-07	1.03 (1.02,1.04)	1.5E-07	1.02 (1.01,1.03)	1.5E-05	1.03 (1.02,1.04)	5.1E-08	1.03 (1.02,1.04)	3.7E-09
mental disorders	297.1	Suicidal ideation	74668	5503	1.04 (1.03,1.05)	1.7E-13	1.04 (1.02,1.05)	1.3E-06	1.03 (1.02,1.04)	1.8E-07	1.02 (1.01,1.03)	1.4E-05	1.03 (1.02,1.04)	8.5E-08	1.03 (1.02,1.04)	5.7E-09

© 2022 Bigdeli TB et al. *JAMA Psychiatry*.

mental disorders	300	Anxiety disorders	36301	40111	1.02 (1.02,1.03)	3.2E-18	1.02 (1.01,1.02)	5.3E-09	1.01 (1,1.01)	2.0E-02	1.02 (1.01,1.02)	7.4E-08	1.02 (1.01,1.02)	3.2E-11	1.02 (1.01,1.02)	6.7E-07
mental disorders	300.1	Anxiety disorder	51938	21915	1.02 (1.02,1.03)	1.6E-16	1.02 (1.01,1.03)	4.5E-10	1.01 (1.01,1.02)	7.1E-05	1.02 (1.01,1.02)	1.1E-08	1.02 (1.01,1.03)	5.8E-11	1.02 (1.01,1.02)	3.9E-08
mental disorders	300.9	Posttraumatic stress disorder	52155	26603	1.01 (1.01,1.02)	8.0E-07	1.01 (1,1.01)	3.8E-03	1 (0.99,1.01)	8.2E-01	1 (1,1.01)	7.3E-02	1.01 (1,1.01)	2.6E-03	1 (1,1.01)	1.0E-01
mental disorders	301	Personality disorders	75463	4935	1.04 (1.03,1.05)	3.1E-15	1.03 (1.02,1.05)	1.1E-05	1.03 (1.02,1.04)	6.2E-09	1.03 (1.02,1.04)	2.0E-06	1.03 (1.02,1.04)	3.2E-09	1.04 (1.02,1.05)	8.7E-11
mental disorders	301.2	Antisocial/borderline personality disorder	79773	2083	1.05 (1.04,1.07)	3.4E-10	1.05 (1.02,1.07)	1.5E-04	1.04 (1.02,1.06)	7.8E-07	1.03 (1.02,1.05)	5.5E-05	1.04 (1.02,1.06)	2.7E-06	1.04 (1.03,1.06)	8.6E-08
mental disorders	304	Adjustment reaction	55082	18851	1.02 (1.01,1.02)	9.1E-09	1.02 (1.01,1.02)	4.1E-06	1.01 (1,1.01)	2.0E-02	1.01 (1.01,1.02)	2.7E-05	1.01 (1.01,1.02)	3.2E-06	1.01 (1.01,1.02)	2.6E-04
mental disorders	306	Other mental disorder	62819	9537	1.02 (1.02,1.03)	1.8E-09	1.02 (1.01,1.03)	3.6E-04	1.01 (1.01,1.02)	3.3E-04	1.01 (1.01,1.02)	4.9E-04	1.02 (1.01,1.03)	1.6E-05	1.02 (1.01,1.03)	1.3E-05
mental disorders	316	Substance addiction and disorders	55813	22948	1.02 (1.02,1.03)	2.2E-16	1.02 (1.01,1.02)	2.7E-07	1.02 (1.01,1.02)	5.5E-07	1.02 (1.01,1.02)	5.2E-07	1.02 (1.01,1.02)	9.5E-11	1.02 (1.01,1.02)	8.5E-10
mental disorders	317.1	Alcoholism	58054	19278	1.02 (1.01,1.03)	2.1E-10	1.01 (1.01,1.02)	7.7E-05	1.01 (1,1.02)	5.2E-04	1.01 (1,1.02)	5.5E-04	1.01 (1.01,1.02)	1.9E-06	1.01 (1.01,1.02)	7.1E-06
mental disorders	318	Tobacco use disorder	43079	33056	1.02 (1.01,1.02)	2.4E-10	1.01 (1.01,1.02)	1.3E-05	1.01 (1.01,1.02)	4.1E-05	1.01 (1.01,1.02)	2.0E-05	1.01 (1.01,1.02)	1.4E-07	1.01 (1.01,1.02)	1.0E-06
musculo-skeletal	735.2	Acquired toe deformities	69294	8813	1.02 (1.01,1.03)	8.3E-07	1.02 (1.01,1.03)	6.3E-05	1.02 (1.01,1.02)	1.2E-04	1.02 (1.01,1.03)	3.0E-05	1.02 (1.01,1.03)	1.8E-05	1.02 (1.01,1.03)	3.5E-05
musculo-skeletal	745	Pain in joint	15565	59241	1.02 (1.01,1.02)	2.6E-06	1.02 (1.01,1.02)	2.5E-06	1.01 (1,1.02)	6.2E-03	1.01 (1.01,1.02)	2.1E-04	1.01 (1.01,1.02)	1.6E-04	1.01 (1,1.02)	3.8E-03
neuro-logical	327	Sleep disorders	47798	23542	1.01 (1.01,1.02)	6.0E-06	1.01 (1.01,1.02)	5.1E-05	1 (1,1.01)	1.5E-01	1.01 (1,1.01)	1.4E-02	1.01 (1,1.01)	4.5E-03	1.01 (1,1.01)	7.0E-02
neuro-logical	338	Pain	56011	15018	1.02 (1.01,1.02)	3.4E-06	1.01 (1.01,1.02)	1.0E-04	1.01 (1,1.01)	1.3E-02	1.01 (1,1.02)	3.2E-03	1.01 (1,1.02)	1.6E-03	1.01 (1,1.02)	5.5E-03
neuro-logical	339	Other headache syndromes	54393	17396	1.02 (1.01,1.03)	5.6E-09	1.02 (1.01,1.02)	6.2E-07	1.01 (1,1.02)	3.6E-04	1.01 (1.01,1.02)	2.8E-05	1.01 (1.01,1.02)	3.5E-04	1.01 (1.01,1.02)	1.7E-04
respiratory	465	Acute upper respiratory infections of multiple or unspecified sites	46098	21217	1.02 (1.02,1.03)	6.2E-16	1.02 (1.01,1.03)	2.7E-09	1.02 (1.01,1.02)	1.9E-09	1.02 (1.01,1.03)	1.5E-10	1.02 (1.01,1.03)	2.4E-11	1.02 (1.01,1.03)	7.6E-11
respiratory	496	Chronic airway obstruction	61946	14621	1.02 (1.01,1.03)	3.6E-09	1.02 (1.01,1.02)	5.8E-05	1.01 (1.01,1.02)	1.3E-05	1.01 (1.01,1.02)	1.4E-05	1.02 (1.01,1.02)	4.7E-07	1.02 (1.01,1.02)	2.8E-06
respiratory	497	Bronchitis	69829	4681	1.03 (1.02,1.04)	2.8E-07	1.02 (1.01,1.04)	9.2E-05	1.02 (1.01,1.03)	5.8E-05	1.02 (1.01,1.03)	4.3E-05	1.02 (1.01,1.04)	8.1E-06	1.02 (1.01,1.03)	1.8E-05
respiratory	512	Other symptoms of respiratory system	34852	33303	1.02 (1.02,1.03)	6.0E-14	1.02 (1.01,1.03)	7.7E-11	1.02 (1.01,1.02)	8.8E-08	1.02 (1.01,1.02)	3.6E-09	1.02 (1.01,1.02)	2.4E-10	1.02 (1.01,1.02)	9.8E-09
respiratory	512.7	Shortness of breath	56849	15224	1.02 (1.01,1.02)	2.4E-06	1.01 (1.01,1.02)	5.5E-05	1.01 (1,1.02)	1.8E-03	1.01 (1,1.02)	6.6E-04	1.01 (1.01,1.02)	9.1E-05	1.01 (1,1.02)	5.5E-04
respiratory	512.8	Cough	54075	14679	1.02 (1.01,1.03)	1.0E-09	1.02 (1.01,1.03)	6.9E-07	1.02 (1.01,1.02)	8.8E-06	1.02 (1.01,1.02)	1.0E-06	1.02 (1.01,1.02)	1.6E-07	1.02 (1.01,1.02)	2.7E-06
sense organs	386.9	Dizziness and giddiness (Light-headedness and vertigo)	62691	10111	1.02 (1.01,1.03)	8.6E-07	1.02 (1.01,1.03)	2.4E-05	1.01 (1.01,1.02)	6.2E-04	1.02 (1.01,1.02)	9.8E-05	1.01 (1.01,1.02)	1.2E-04	1.01 (1.01,1.02)	2.4E-04
symptoms	760	Back pain	26406	47752	1.02 (1.01,1.03)	7.3E-13	1.02 (1.01,1.02)	5.2E-10	1.01 (1.01,1.02)	1.2E-06	1.02 (1.01,1.02)	5.2E-09	1.02 (1.01,1.02)	2.5E-09	1.01 (1.01,1.02)	7.4E-07
symptoms	770	Myalgia and myositis unspecified	70525	5917	1.02 (1.01,1.03)	4.8E-06	1.02 (1.01,1.03)	5.6E-05	1.02 (1.01,1.03)	1.9E-03	1.02 (1.01,1.03)	4.5E-04	1.02 (1.01,1.03)	4.3E-04	1.02 (1.01,1.03)	8.2E-04
symptoms	785	Abdominal pain	49712	20812	1.02 (1.01,1.02)	3.6E-10	1.02 (1.01,1.02)	1.4E-07	1.01 (1.01,1.02)	2.0E-05	1.01 (1.01,1.02)	3.4E-06	1.02 (1.01,1.02)	4.5E-07	1.01 (1.01,1.02)	5.1E-06
symptoms	789	Nausea and vomiting	67505	6884	1.03 (1.02,1.04)	1.3E-08	1.02 (1.01,1.03)	8.9E-05	1.02 (1.01,1.03)	2.7E-05	1.02 (1.01,1.03)	2.8E-05	1.02 (1.01,1.03)	3.1E-06	1.02 (1.01,1.03)	5.0E-06
symptoms	798	Malaise and fatigue	61002	11331	1.02 (1.01,1.03)	9.1E-08	1.02 (1.01,1.03)	1.1E-05	1.01 (1.01,1.02)	1.8E-04	1.01 (1.01,1.02)	5.7E-05	1.02 (1.01,1.02)	1.3E-05	1.01 (1.01,1.02)	6.4E-05

© 2022 Bigdeli TB et al. *JAMA Psychiatry*.

eTable 31. Associations of neuropsychiatric PRSs with hierarchical diagnoses in EA participants. For each PRS and psychosis or affective spectrum diagnosis, $R^2_{Nagelkerke}$ and $R^2_{liability}$ are the variance explained in terms of Nagelkerke's R^2 and on the liability scale (assuming K=0.01 for comparability); OR is the odds ratio (and 95% CI) per SD unit increase; and *p*-value is the significance of the PRS in the logistic model.

Training Data	Diagnosis	Cases	Controls	$R^2_{Nagelkerke}$	$R^2_{liability}$	OR (95% CI)	*p*-value
PGC3-BIP	Bipolar II	793	111105	2.3E-03	3.7E-03	1.19 (1.1, 1.28)	4.3E-06
PGC3-BIP	Bipolar I	4689	111105	8.9E-03	9.1E-03	1.33 (1.29, 1.38)	4.2E-69
PGC3-BIP	Bipolar I (psychosis)	1755	111105	1.6E-02	2.2E-02	1.53 (1.46, 1.61)	1.6E-61
PGC3-BIP	Cyclothymia	364	111105	1.6E-03	2.9E-03	1.16 (1.05, 1.3)	5.5E-03
PGC3-BIP	Delusional	1184	111105	9.9E-03	1.5E-02	1.41 (1.32, 1.49)	1.1E-28
PGC3-BIP	Dysthymia	1602	111105	1.0E-03	1.4E-03	1.11 (1.05, 1.17)	6.6E-05
PGC3-BIP	MDD	93769	111105	2.7E-03	1.1E-03	1.11 (1.1, 1.12)	6.2E-101
PGC3-BIP	MDD (psychosis)	13346	111105	3.6E-03	2.6E-03	1.16 (1.14, 1.19)	1.3E-52
PGC3-BIP	Psychosis	4274	111105	6.8E-03	7.1E-03	1.27 (1.23, 1.31)	2.0E-48
PGC3-BIP	Schizophrenia	4722	111105	1.7E-02	1.7E-02	1.47 (1.42, 1.51)	4.0E-127
PGC3-BIP	Schizoaffective	2037	111105	1.4E-02	1.8E-02	1.47 (1.41, 1.54)	5.8E-59
gSEM-BIP	Bipolar II	793	111105	2.7E-05	4.3E-05	0.98 (0.91, 1.05)	6.2E-01
gSEM-BIP	Bipolar I	4689	111105	1.1E-06	1.2E-06	1 (0.97, 1.03)	8.4E-01
gSEM-BIP	Bipolar I (psychosis)	1755	111105	3.2E-04	4.3E-04	1.06 (1.01, 1.11)	2.0E-02
gSEM-BIP	Cyclothymia	364	111105	3.9E-05	7.0E-05	0.98 (0.88, 1.08)	6.7E-01
gSEM-BIP	Delusional	1184	111105	2.7E-05	4.0E-05	0.98 (0.93, 1.04)	5.6E-01
gSEM-BIP	Dysthymia	1602	111105	6.9E-05	9.5E-05	0.97 (0.93, 1.02)	3.0E-01
gSEM-BIP	MDD	93769	111105	8.2E-04	3.4E-04	0.95 (0.94, 0.96)	1.4E-31
gSEM-BIP	MDD (psychosis)	13346	111105	9.6E-04	6.9E-04	0.93 (0.91, 0.94)	2.2E-15
gSEM-BIP	Psychosis	4274	111105	2.4E-04	2.6E-04	0.96 (0.93, 0.99)	5.5E-03
gSEM-BIP	Schizophrenia	4722	111105	6.5E-04	6.6E-04	0.93 (0.9, 0.96)	2.3E-06
gSEM-BIP	Schizoaffective	2037	111105	1.1E-04	1.4E-04	0.97 (0.92, 1.01)	1.5E-01
gSEM-GEN	Bipolar II	793	111105	6.5E-03	1.0E-02	1.33 (1.24, 1.43)	1.8E-14
gSEM-GEN	Bipolar I	4689	111105	1.8E-02	1.8E-02	1.5 (1.45, 1.55)	1.9E-135
gSEM-GEN	Bipolar I (psychosis)	1755	111105	2.2E-02	3.0E-02	1.64 (1.56, 1.73)	5.2E-84
gSEM-GEN	Cyclothymia	364	111105	5.1E-03	9.4E-03	1.31 (1.18, 1.46)	6.7E-07
gSEM-GEN	Delusional	1184	111105	1.3E-02	1.9E-02	1.47 (1.39, 1.56)	1.4E-37
gSEM-GEN	Dysthymia	1602	111105	2.3E-03	3.2E-03	1.17 (1.11, 1.23)	1.5E-09
gSEM-GEN	MDD	93769	111105	1.1E-02	4.7E-03	1.23 (1.22, 1.24)	0.0E+00
gSEM-GEN	MDD (psychosis)	13346	111105	1.2E-02	8.9E-03	1.32 (1.3, 1.35)	1.9E-176
gSEM-GEN	Psychosis	4274	111105	1.2E-02	1.3E-02	1.38 (1.34, 1.42)	2.1E-87
gSEM-GEN	Schizophrenia	4722	111105	2.2E-02	2.3E-02	1.54 (1.49, 1.59)	1.4E-164
gSEM-GEN	Schizoaffective	2037	111105	2.4E-02	3.1E-02	1.66 (1.58, 1.74)	6.2E-100
gSEM-DEP	Bipolar II	793	111105	4.8E-04	7.5E-04	1.08 (1, 1.16)	3.7E-02
gSEM-DEP	Bipolar I	4689	111105	7.9E-04	8.0E-04	1.09 (1.05, 1.12)	1.6E-07
gSEM-DEP	Bipolar I (psychosis)	1755	111105	1.9E-04	2.5E-04	0.96 (0.91, 1)	7.4E-02
gSEM-DEP	Cyclothymia	364	111105	2.9E-03	5.2E-03	1.22 (1.1, 1.35)	2.2E-04
gSEM-DEP	Delusional	1184	111105	1.1E-03	1.6E-03	0.9 (0.85, 0.95)	2.1E-04
gSEM-DEP	Dysthymia	1602	111105	1.3E-04	1.8E-04	1.04 (0.99, 1.09)	1.5E-01
gSEM-DEP	MDD	93769	111105	2.8E-03	1.2E-03	1.11 (1.1, 1.12)	3.5E-105
gSEM-DEP	MDD (psychosis)	13346	111105	2.0E-03	1.4E-03	1.11 (1.09, 1.14)	1.2E-29
gSEM-DEP	Psychosis	4274	111105	3.0E-06	3.1E-06	1 (0.96, 1.03)	7.6E-01
gSEM-DEP	Schizophrenia	4722	111105	3.9E-03	3.9E-03	0.84 (0.81, 0.86)	1.9E-30
gSEM-DEP	Schizoaffective	2037	111105	3.3E-04	4.2E-04	0.94 (0.9, 0.99)	1.2E-02
gSEM-SCZ	Bipolar II	793	111105	4.5E-04	7.1E-04	0.93 (0.87, 1)	4.3E-02
gSEM-SCZ	Bipolar I	4689	111105	6.6E-04	6.7E-04	0.93 (0.9, 0.96)	1.7E-06
gSEM-SCZ	Bipolar I (psychosis)	1755	111105	9.2E-05	1.2E-04	0.97 (0.92, 1.02)	2.1E-01
gSEM-SCZ	Cyclothymia	364	111105	1.2E-03	2.1E-03	0.88 (0.79, 0.98)	1.8E-02
gSEM-SCZ	Delusional	1184	111105	1.1E-03	1.6E-03	1.11 (1.05, 1.18)	2.4E-04
gSEM-SCZ	Dysthymia	1602	111105	8.1E-06	1.1E-05	0.99 (0.94, 1.04)	7.2E-01
gSEM-SCZ	MDD	93769	111105	9.8E-04	4.1E-04	0.94 (0.93, 0.95)	1.9E-37
gSEM-SCZ	MDD (psychosis)	13346	111105	3.4E-04	2.4E-04	0.96 (0.94, 0.97)	2.7E-06

© 2022 Bigdeli TB et al. *JAMA Psychiatry*.

gSEM-SCZ	Psychosis	4274	111105	1.5E-04	1.6E-04	1.04 (1, 1.07)	2.8E-02
gSEM-SCZ	Schizophrenia	4722	111105	7.7E-03	7.8E-03	1.28 (1.24, 1.32)	5.7E-59
gSEM-SCZ	Schizoaffective	2037	111105	5.5E-04	7.0E-04	1.08 (1.03, 1.13)	1.3E-03
PGC-MDD (2018)	Bipolar II	793	111105	3.0E-03	4.7E-03	1.21 (1.13, 1.3)	2.1E-07
PGC-MDD (2018)	Bipolar I	4689	111105	4.6E-03	4.7E-03	1.22 (1.19, 1.26)	5.8E-37
PGC-MDD (2018)	Bipolar I (psychosis)	1755	111105	6.5E-03	8.6E-03	1.3 (1.24, 1.36)	1.3E-25
PGC-MDD (2018)	Cyclothymia	364	111105	3.9E-03	7.1E-03	1.26 (1.14, 1.4)	1.5E-05
PGC-MDD (2018)	Delusional	1184	111105	3.1E-03	4.5E-03	1.2 (1.13, 1.27)	6.4E-10
PGC-MDD (2018)	Dysthymia	1602	111105	9.7E-04	1.3E-03	1.1 (1.05, 1.16)	1.0E-04
PGC-MDD (2018)	MDD	93769	111105	5.7E-03	2.4E-03	1.16 (1.15, 1.17)	5.9E-209
PGC-MDD (2018)	MDD (psychosis)	13346	111105	7.1E-03	5.1E-03	1.23 (1.21, 1.26)	1.1E-102
PGC-MDD (2018)	Psychosis	4274	111105	4.9E-03	5.2E-03	1.22 (1.18, 1.26)	1.3E-35
PGC-MDD (2018)	Schizophrenia	4722	111105	6.4E-03	6.6E-03	1.26 (1.22, 1.3)	1.1E-49
PGC-MDD (2018)	Schizoaffective	2037	111105	6.6E-03	8.5E-03	1.3 (1.24, 1.36)	4.5E-29
PGC3-SCZ	Bipolar II	793	111105	2.5E-03	3.9E-03	1.19 (1.11, 1.28)	2.3E-06
PGC3-SCZ	Bipolar I	4689	111105	6.0E-03	6.2E-03	1.26 (1.22, 1.3)	1.0E-47
PGC3-SCZ	Bipolar I (psychosis)	1755	111105	1.2E-02	1.7E-02	1.43 (1.36, 1.5)	2.2E-47
PGC3-SCZ	Cyclothymia	364	111105	3.3E-04	6.0E-04	1.07 (0.96, 1.19)	2.1E-01
PGC3-SCZ	Delusional	1184	111105	1.8E-02	2.6E-02	1.56 (1.47, 1.65)	4.0E-50
PGC3-SCZ	Dysthymia	1602	111105	1.4E-03	1.9E-03	1.13 (1.07, 1.18)	3.0E-06
PGC3-SCZ	MDD	93769	111105	2.4E-03	1.0E-03	1.1 (1.09, 1.11)	5.3E-89
PGC3-SCZ	MDD (psychosis)	13346	111105	3.6E-03	2.6E-03	1.16 (1.14, 1.18)	2.2E-53
PGC3-SCZ	Psychosis	4274	111105	8.5E-03	8.9E-03	1.3 (1.26, 1.34)	2.9E-60
PGC3-SCZ	Schizophrenia	4722	111105	4.1E-02	4.3E-02	1.79 (1.74, 1.85)	4.6E-303
PGC3-SCZ	Schizoaffective	2037	111105	2.2E-02	2.8E-02	1.59 (1.52, 1.67)	2.8E-90
PGC3-BIP	Bipolar II	221	23197	5.2E-04	7.8E-04	0.91 (0.77, 1.08)	2.6E-01
PGC3-BIP	Bipolar I	876	23197	1.4E-03	1.5E-03	1.15 (1.05, 1.25)	2.2E-03
PGC3-BIP	Bipolar I (psychosis)	556	23197	3.9E-03	4.7E-03	1.27 (1.14, 1.42)	1.4E-05
PGC3-BIP	Cyclothymia	74	23197	2.5E-03	4.6E-03	1.26 (0.94, 1.68)	1.2E-01
PGC3-BIP	Delusional	533	23197	3.9E-03	4.8E-03	1.27 (1.14, 1.41)	2.2E-05
PGC3-BIP	Dysthymia	378	23197	9.8E-05	1.3E-04	1.04 (0.91, 1.18)	5.5E-01
PGC3-BIP	MDD	27855	23197	3.1E-04	1.3E-04	1.04 (1.02, 1.06)	4.9E-04
PGC3-BIP	MDD (psychosis)	5645	23197	1.6E-03	8.7E-04	1.11 (1.07, 1.15)	4.8E-08
PGC3-BIP	Psychosis	2419	23197	9.7E-04	7.3E-04	1.1 (1.04, 1.16)	6.6E-04
PGC3-BIP	Schizophrenia	4297	23197	4.8E-03	2.9E-03	1.21 (1.16, 1.26)	1.3E-18
PGC3-BIP	Schizoaffective	1176	23197	2.5E-03	2.5E-03	1.19 (1.1, 1.28)	7.2E-06

© 2022 Bigdeli TB et al. *JAMA Psychiatry*.

eTable 32. Associations of neuropsychiatric PRSs with hierarchical diagnoses in AA participants.
For each PRS and psychosis or affective spectrum diagnosis, $R^2_{Nagelkerke}$ and $R^2_{liability}$ are the variance explained in terms of Nagelkerke's R^2 and on the liability scale (assuming K=0.01 for comparability); OR is the odds ratio (and 95% CI) per SD unit increase; and *p*-value is the significance of the PRS in the logistic model.

Training Data	Diagnosis	Cases	Controls	$R^2_{Nagelkerke}$	$R^2_{liability}$	OR (95% CI)	*p*-value
gSEM-BIP	Bipolar II	221	23197	7.4E-04	1.1E-03	0.91 (0.8, 1.04)	1.8E-01
gSEM-BIP	Bipolar I	876	23197	1.1E-04	1.1E-04	0.97 (0.9, 1.04)	4.0E-01
gSEM-BIP	Bipolar I (psychosis)	556	23197	4.6E-04	5.6E-04	1.07 (0.98, 1.16)	1.4E-01
gSEM-BIP	Cyclothymia	74	23197	4.4E-03	8.0E-03	1.27 (1.01, 1.6)	3.9E-02
gSEM-BIP	Delusional	533	23197	1.1E-04	1.3E-04	1.03 (0.95, 1.13)	4.8E-01
gSEM-BIP	Dysthymia	378	23197	2.0E-05	2.7E-05	1.01 (0.91, 1.12)	7.9E-01
gSEM-BIP	MDD	27855	23197	1.3E-04	5.5E-05	0.98 (0.96, 1)	2.3E-02
gSEM-BIP	MDD (psychosis)	5645	23197	2.0E-05	1.1E-05	0.99 (0.96, 1.02)	5.4E-01
gSEM-BIP	Psychosis	2419	23197	6.3E-04	4.7E-04	0.94 (0.9, 0.98)	6.1E-03
gSEM-BIP	Schizophrenia	4297	23197	1.8E-04	1.1E-04	0.97 (0.94, 1)	8.7E-02
gSEM-BIP	Schizoaffective	1176	23197	4.4E-05	4.2E-05	0.98 (0.92, 1.04)	5.5E-01
gSEM-GEN	Bipolar II	221	23197	6.2E-06	9.3E-06	0.99 (0.84, 1.17)	9.0E-01
gSEM-GEN	Bipolar I	876	23197	4.0E-03	4.2E-03	1.26 (1.15, 1.37)	2.9E-07
gSEM-GEN	Bipolar I (psychosis)	556	23197	7.0E-03	8.4E-03	1.37 (1.23, 1.52)	7.2E-09
gSEM-GEN	Cyclothymia	74	23197	1.7E-03	3.0E-03	1.2 (0.91, 1.59)	2.0E-01
gSEM-GEN	Delusional	533	23197	5.5E-03	6.6E-03	1.32 (1.18, 1.47)	5.8E-07
gSEM-GEN	Dysthymia	378	23197	1.8E-04	2.4E-04	0.95 (0.83, 1.08)	4.2E-01
gSEM-GEN	MDD	27855	23197	1.4E-03	5.9E-04	1.09 (1.06, 1.11)	8.1E-14
gSEM-GEN	MDD (psychosis)	5645	23197	3.2E-03	1.8E-03	1.16 (1.12, 1.2)	1.1E-14
gSEM-GEN	Psychosis	2419	23197	2.9E-03	2.2E-03	1.17 (1.11, 1.24)	4.7E-09
gSEM-GEN	Schizophrenia	4297	23197	7.0E-03	4.3E-03	1.25 (1.2, 1.3)	3.9E-26
gSEM-GEN	Schizoaffective	1176	23197	4.8E-03	4.6E-03	1.26 (1.17, 1.36)	7.7E-10
gSEM-DEP	Bipolar II	221	23197	1.6E-03	2.4E-03	1.16 (1, 1.35)	5.2E-02
gSEM-DEP	Bipolar I	876	23197	3.3E-04	3.5E-04	1.06 (0.98, 1.15)	1.4E-01
gSEM-DEP	Bipolar I (psychosis)	556	23197	1.9E-05	2.3E-05	1.01 (0.92, 1.12)	7.6E-01
gSEM-DEP	Cyclothymia	74	23197	4.5E-06	8.2E-06	0.99 (0.77, 1.28)	9.5E-01
gSEM-DEP	Delusional	533	23197	4.0E-04	4.8E-04	0.94 (0.85, 1.03)	1.8E-01
gSEM-DEP	Dysthymia	378	23197	3.0E-04	3.9E-04	0.94 (0.84, 1.06)	3.0E-01
gSEM-DEP	MDD	27855	23197	1.1E-04	4.6E-05	1.02 (1, 1.04)	3.6E-02
gSEM-DEP	MDD (psychosis)	5645	23197	2.8E-05	1.6E-05	1.01 (0.98, 1.05)	4.7E-01
gSEM-DEP	Psychosis	2419	23197	1.9E-05	1.5E-05	1.01 (0.97, 1.06)	6.3E-01
gSEM-DEP	Schizophrenia	4297	23197	1.3E-03	7.7E-04	0.92 (0.89, 0.95)	6.2E-06
gSEM-DEP	Schizoaffective	1176	23197	3.6E-05	3.4E-05	0.98 (0.92, 1.05)	5.9E-01
gSEM-SCZ	Bipolar II	221	23197	2.0E-04	3.0E-04	0.94 (0.8, 1.11)	4.9E-01
gSEM-SCZ	Bipolar I	876	23197	1.8E-06	1.9E-06	1 (0.92, 1.08)	9.1E-01
gSEM-SCZ	Bipolar I (psychosis)	556	23197	4.0E-04	4.8E-04	0.93 (0.84, 1.03)	1.7E-01
gSEM-SCZ	Cyclothymia	74	23197	9.2E-04	1.7E-03	0.88 (0.67, 1.15)	3.4E-01
gSEM-SCZ	Delusional	533	23197	3.2E-04	3.9E-04	1.07 (0.96, 1.18)	2.2E-01
gSEM-SCZ	Dysthymia	378	23197	1.6E-04	2.2E-04	1.05 (0.93, 1.19)	4.4E-01
gSEM-SCZ	MDD	27855	23197	1.2E-07	5.2E-08	1 (0.98, 1.02)	9.4E-01
gSEM-SCZ	MDD (psychosis)	5645	23197	3.7E-07	2.0E-07	1 (0.97, 1.04)	9.3E-01
gSEM-SCZ	Psychosis	2419	23197	4.3E-05	3.3E-05	1.02 (0.97, 1.07)	4.7E-01
gSEM-SCZ	Schizophrenia	4297	23197	1.7E-03	1.0E-03	1.11 (1.07, 1.16)	1.6E-07
gSEM-SCZ	Schizoaffective	1176	23197	1.3E-04	1.2E-04	1.04 (0.97, 1.11)	3.2E-01
PGC-MDD (2018)	Bipolar II	221	23197	6.8E-07	1.0E-06	1 (0.82, 1.2)	9.7E-01
PGC-MDD (2018)	Bipolar I	876	23197	1.2E-05	1.3E-05	1.01 (0.92, 1.12)	7.7E-01
PGC-MDD (2018)	Bipolar I (psychosis)	556	23197	1.6E-04	2.0E-04	0.95 (0.84, 1.07)	3.8E-01
PGC-MDD (2018)	Cyclothymia	74	23197	3.9E-05	7.2E-05	0.97 (0.69, 1.34)	8.5E-01
PGC-MDD (2018)	Delusional	533	23197	6.5E-06	7.9E-06	0.99 (0.88, 1.12)	8.6E-01
PGC-MDD (2018)	Dysthymia	378	23197	1.9E-04	2.5E-04	1.06 (0.92, 1.22)	4.1E-01
PGC-MDD (2018)	MDD	27855	23197	1.6E-05	6.6E-06	1.01 (0.99, 1.04)	4.3E-01
PGC-MDD (2018)	MDD (psychosis)	5645	23197	3.4E-05	1.9E-05	1.02 (0.98, 1.06)	4.3E-01
PGC-MDD (2018)	Psychosis	2419	23197	2.0E-04	1.5E-04	1.05 (0.99, 1.11)	1.2E-01

© 2022 Bigdeli TB et al. *JAMA Psychiatry*.

PGC-MDD (2018)	Schizophrenia	4297	23197	1.8E-04	1.1E-04	1.04 (0.99, 1.09)	8.5E-02
PGC-MDD (2018)	Schizoaffective	1176	23197	9.5E-08	9.1E-08	1 (0.92, 1.09)	9.8E-01
PGC3-SCZ	Bipolar II	221	23197	1.0E-04	1.5E-04	1.04 (0.88, 1.23)	6.2E-01
PGC3-SCZ	Bipolar I	876	23197	1.9E-03	2.0E-03	1.17 (1.07, 1.27)	3.4E-04
PGC3-SCZ	Bipolar I (psychosis)	556	23197	3.2E-03	3.9E-03	1.24 (1.11, 1.37)	7.4E-05
PGC3-SCZ	Cyclothymia	74	23197	5.3E-05	9.7E-05	1.03 (0.77, 1.37)	8.2E-01
PGC3-SCZ	Delusional	533	23197	1.6E-03	1.9E-03	1.16 (1.04, 1.29)	6.3E-03
PGC3-SCZ	Dysthymia	378	23197	9.6E-05	1.3E-04	1.04 (0.91, 1.18)	5.6E-01
PGC3-SCZ	MDD	27855	23197	6.8E-04	2.8E-04	1.06 (1.04, 1.08)	2.2E-07
PGC3-SCZ	MDD (psychosis)	5645	23197	1.1E-03	5.9E-04	1.09 (1.05, 1.13)	6.5E-06
PGC3-SCZ	Psychosis	2419	23197	2.9E-03	2.1E-03	1.17 (1.11, 1.23)	4.3E-09
PGC3-SCZ	Schizophrenia	4297	23197	1.2E-02	7.3E-03	1.33 (1.28, 1.38)	6.6E-44
PGC3-SCZ	Schizoaffective	1176	23197	2.6E-03	2.5E-03	1.18 (1.1, 1.27)	5.9E-06

© 2022 Bigdeli TB et al. *JAMA Psychiatry*.

eFigure 1. Odds ratios for top decile of SCZ PRS in EA participants.

For comparisons of the top PRS decile versus the remaining 90% (circles) and bottom 10% (triangles) displayed; error bars indicate the 95% CI for the estimated effect on selected phecodes. Displayed results are limited to phecodes with p-values < 10^{-10} (in either comparison).

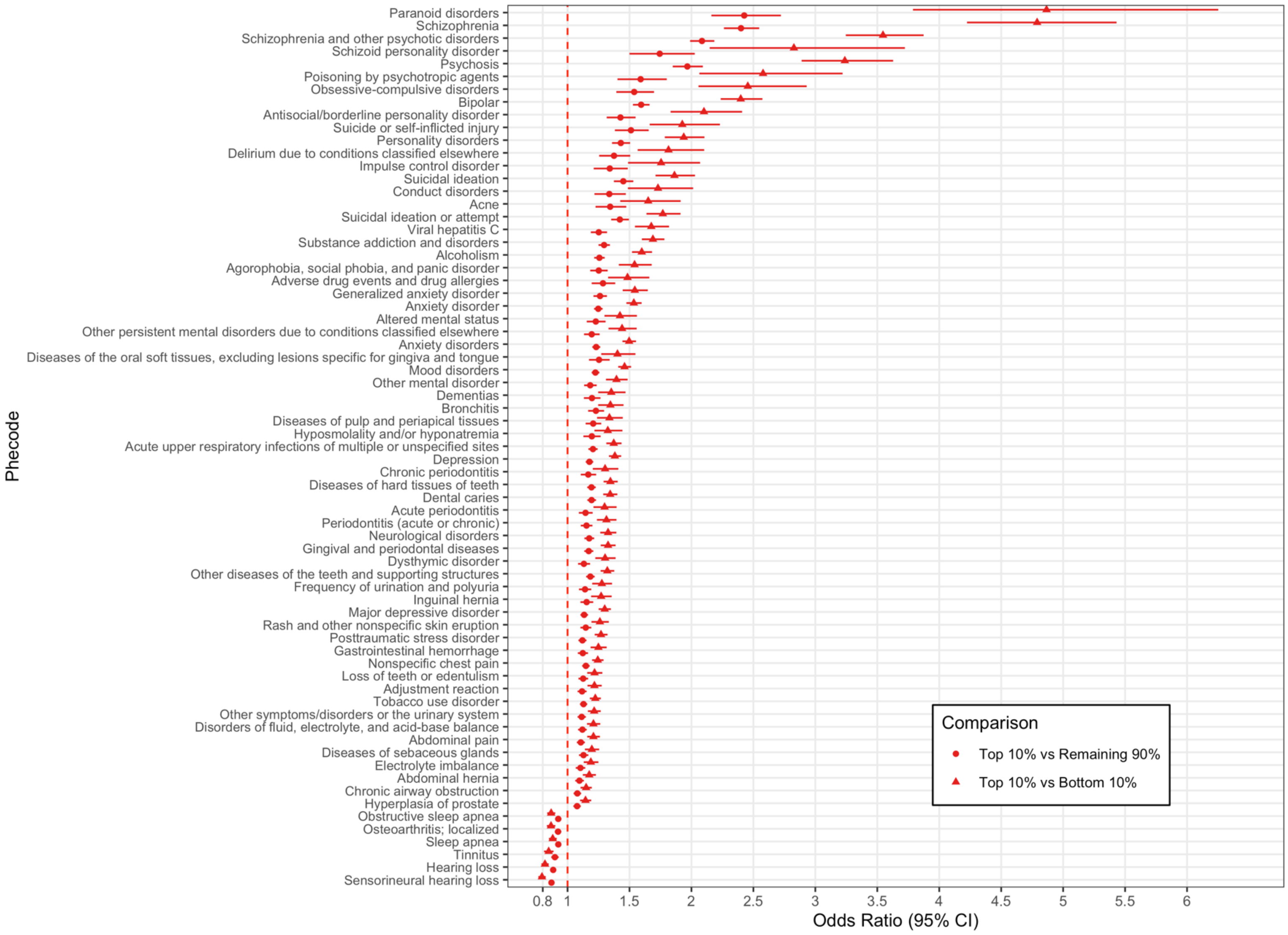

© 2022 Bigdeli TB et al. *JAMA Psychiatry*.

eFigure 2. Odds ratios for top deciles of BIP PRS in EA participants.

For comparisons of the top PRS decile versus the remaining 90% (circles) and bottom 10% (triangles) displayed; error bars indicate the 95% CI for the estimated effect on selected phecodes. Displayed results are limited to phecodes with p-values < 10^{-10} (in either comparison).

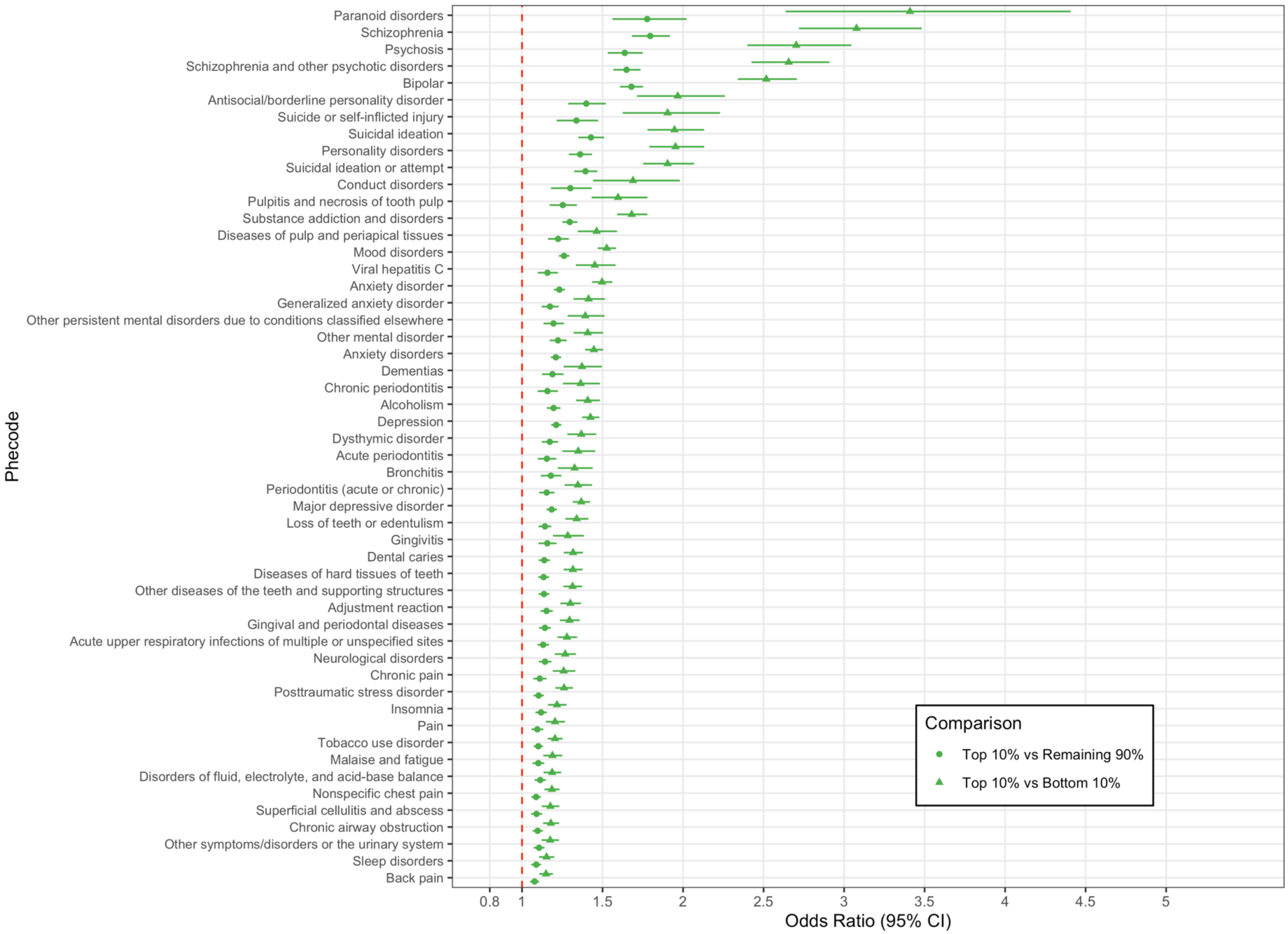

© 2022 Bigdeli TB et al. *JAMA Psychiatry*.

eFigure 3. Odds ratios for top decile of DEP PRS in EA participants.
For comparisons of the top PRS decile versus the remaining 90% (circles) and bottom 10% (triangles) displayed; error bars indicate the 95% CI for the estimated effect on selected phecodes. Displayed results are limited to phecodes with p-values < 10^{-20} (in either comparison).

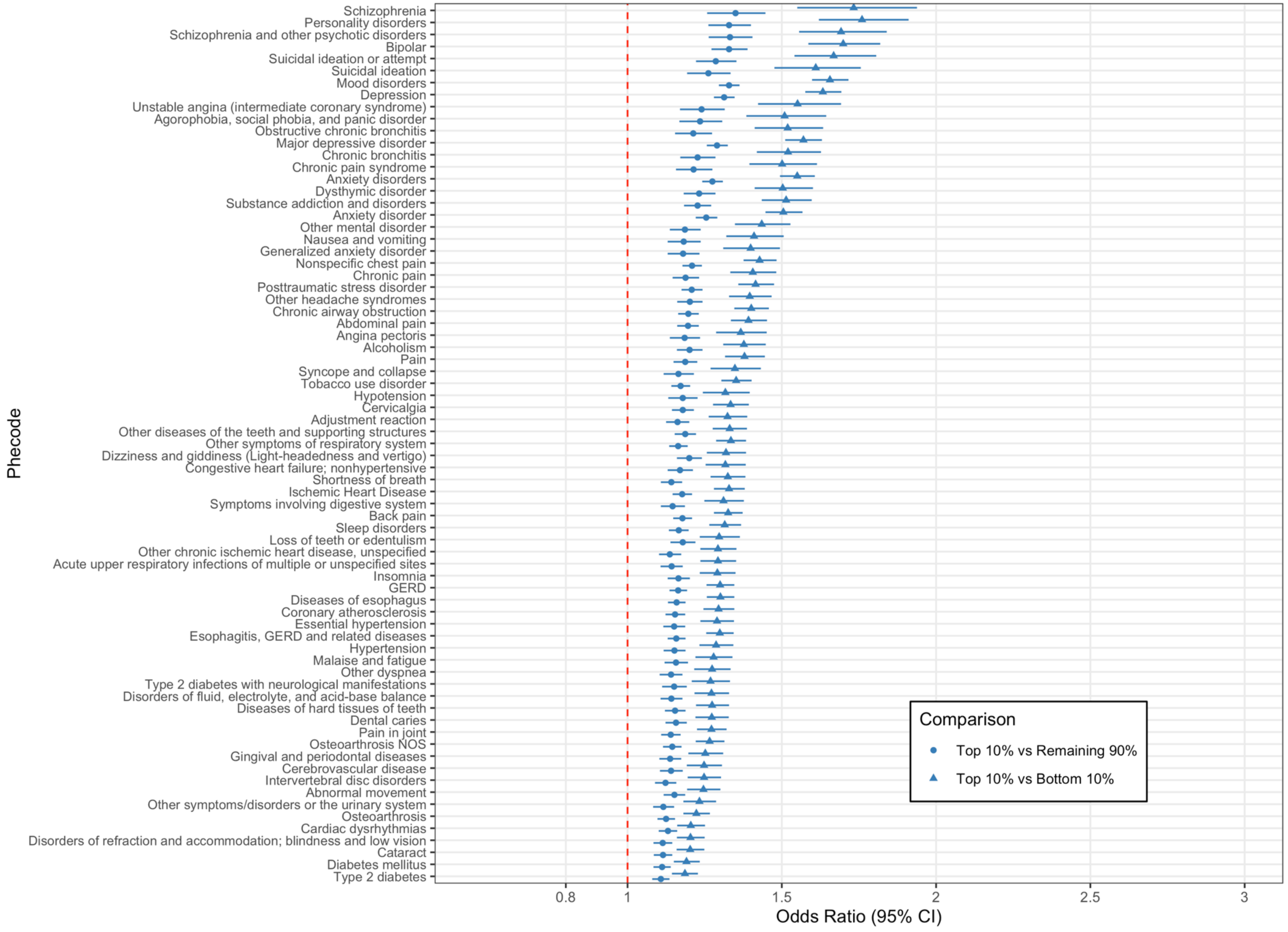

© 2022 Bigdeli TB et al. *JAMA Psychiatry*.

eFigure 4. PheWAS results for neuropsychiatric PRS.
PheWAS of SCZ, BIP, and DEP PRS across 1,650 disease categories are displayed. A red dotted line indicates an approximate Bonferroni adjusted *p*-value threshold for the number of Phecodes tested. Part of this figure has been republished with permission.

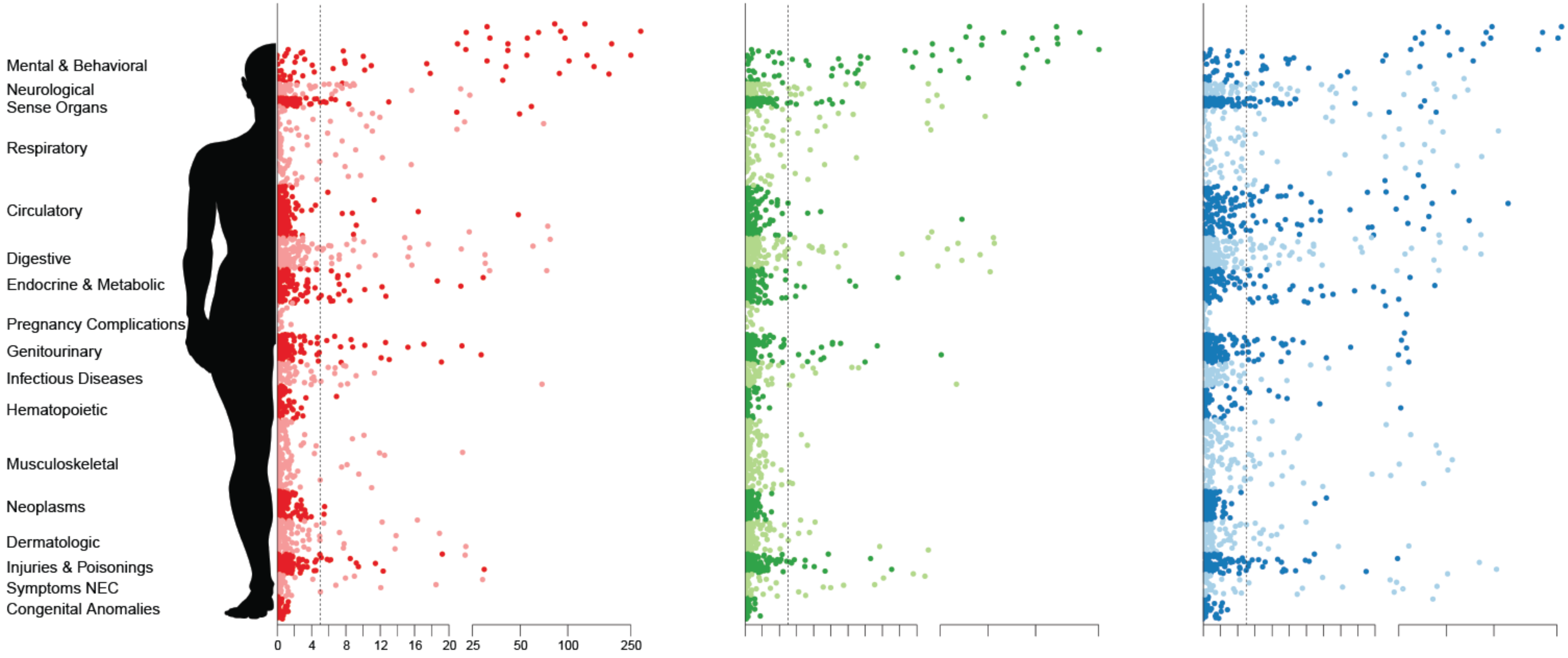

© 2022 Bigdeli TB et al. *JAMA Psychiatry*.

eFigure 5. Relative enrichments of SCZ versus BIP PheWAS results across disease categories.
For each disease category, ranked *p*-values for SCZ are plotted against ranked p-values for BIP, as in a quantile-quantile (QQ) plot. Displayed results are based on EA participants. The diagonal line indicates expectation under the null of no relative enrichment in either results set.

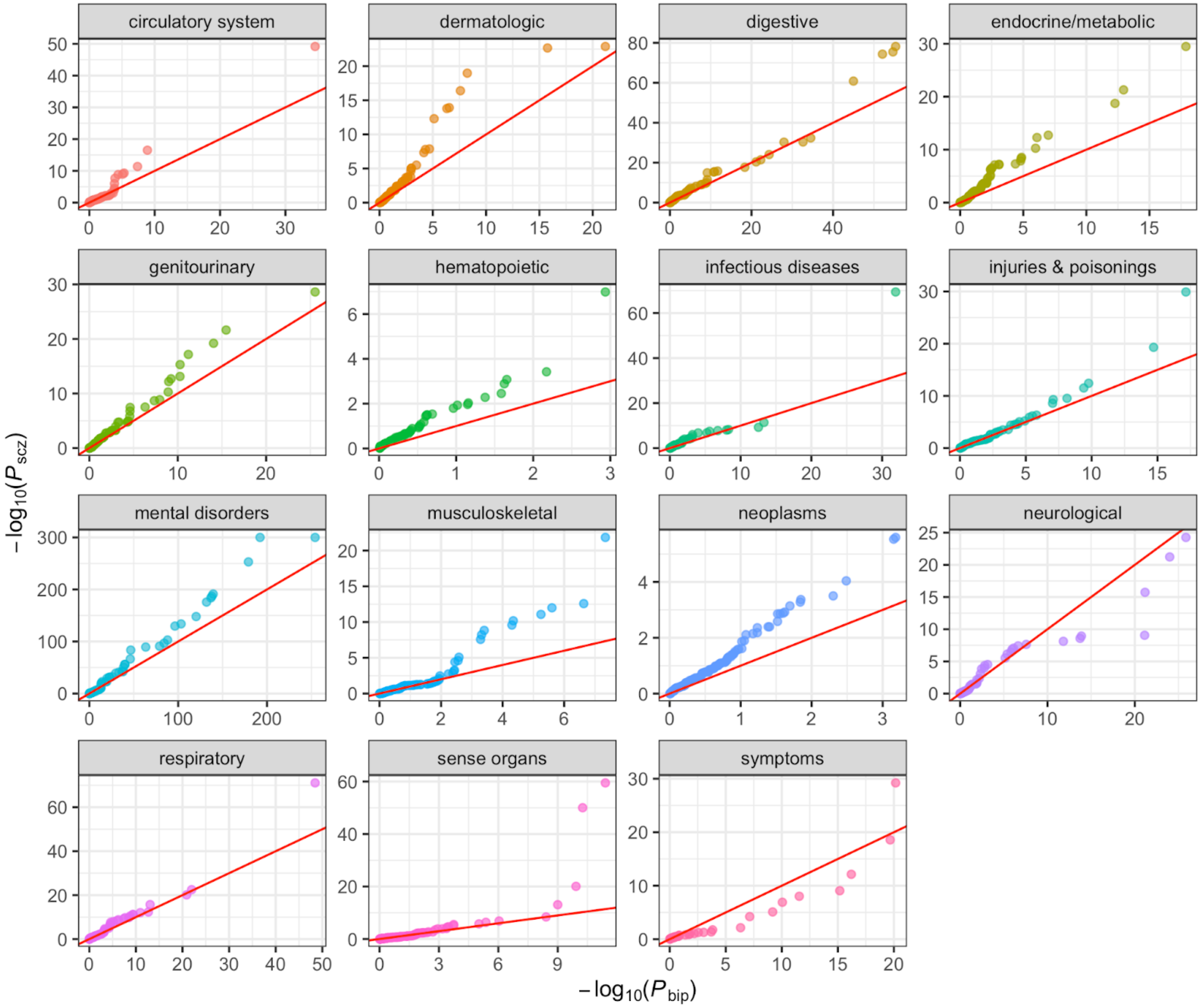

© 2022 Bigdeli TB et al. *JAMA Psychiatry*.

eFigure 6. Relative enrichments of SCZ and DEP PheWAS results across disease categories.
For each disease category, ranked *p*-values for SCZ are plotted against ranked p-values for DEP, as in a quantile-quantile (QQ) plot. Displayed results are based on EA participants. The diagonal line indicates expectation under the null of no relative enrichment in either results set.

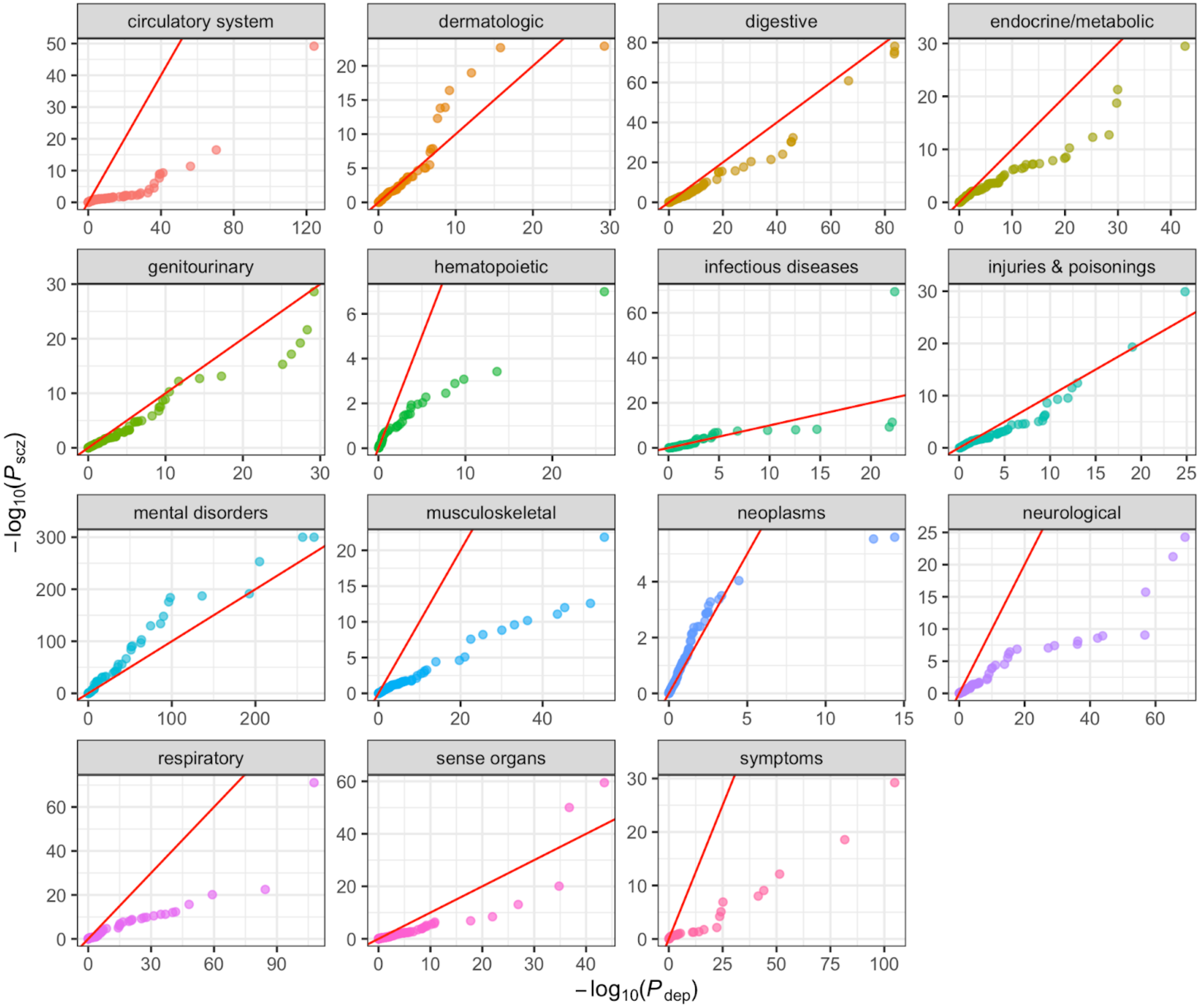

© 2022 Bigdeli TB et al. *JAMA Psychiatry*.

eFigure 7. Relative enrichments of BIP and DEP PheWAS results across disease categories.
For each disease category, ranked *p*-values for BIP are plotted against ranked p-values for DEP, as in a quantile-quantile (QQ) plot. Displayed results are based on EA participants. The diagonal line indicates expectation under the null of no relative enrichment in either results set.

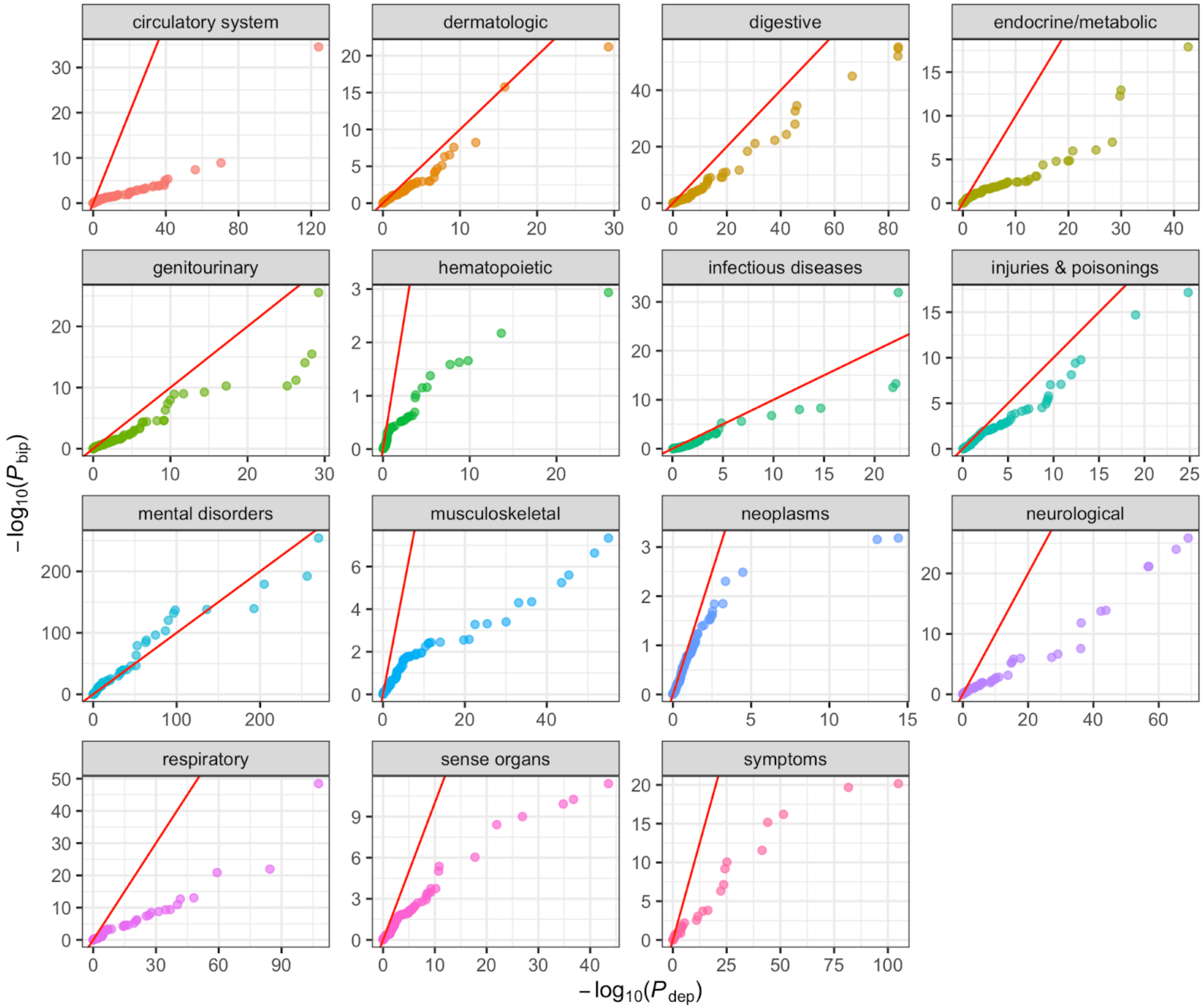

© 2022 Bigdeli TB et al. *JAMA Psychiatry*.

eFigure 8. Odds ratios for top deciles of SCZ PRS in AA participants.
For comparisons of the top PRS decile versus the remaining 90% (circles) and bottom 10% (triangles) displayed; error bars indicate the 95% CI for the estimated effect on selected phecodes. Displayed results are limited to phecodes with p-values < 10^{-5} (in either comparison) or replications ($p<0.05$) of significant EA findings.

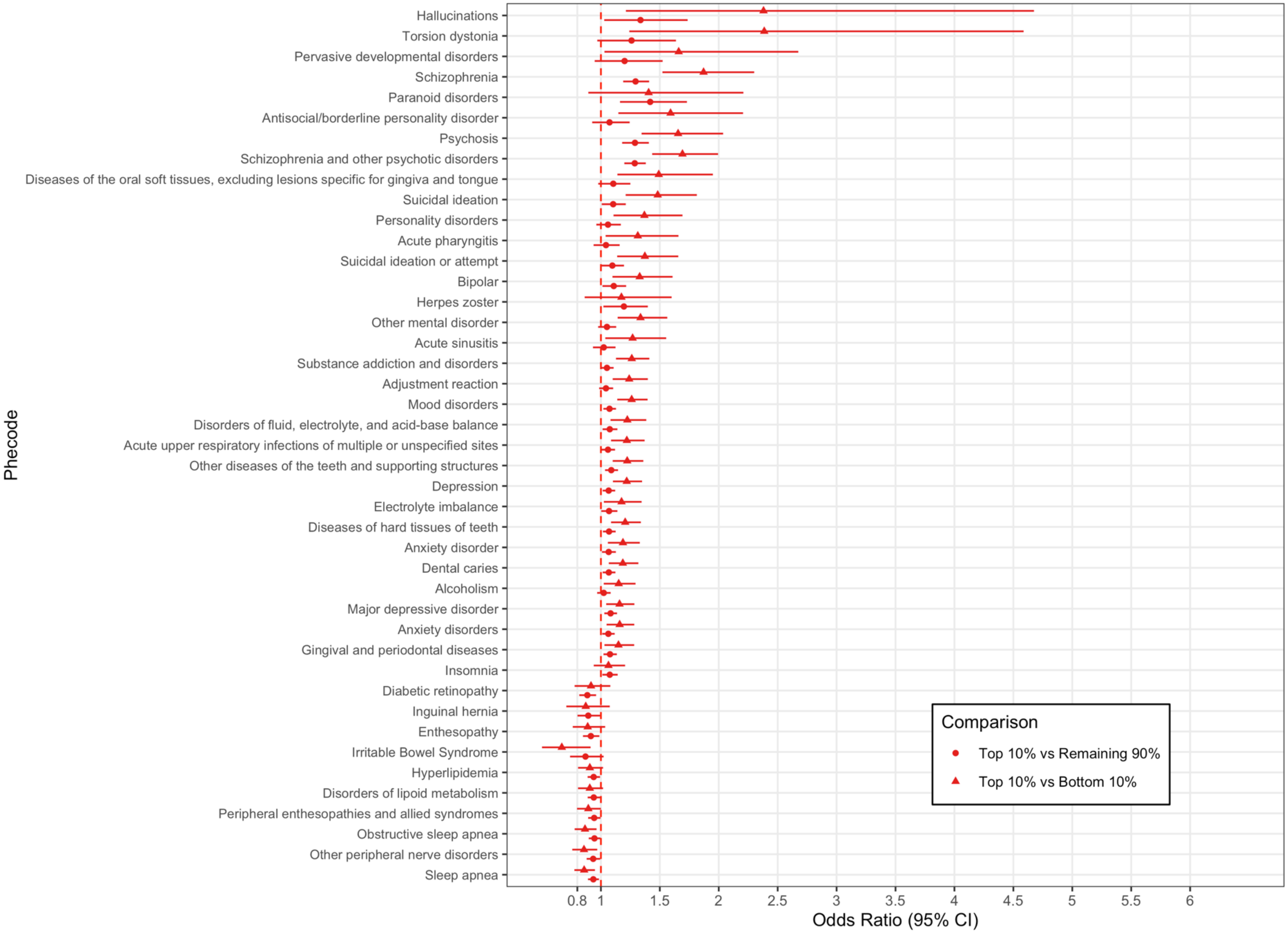

© 2022 Bigdeli TB et al. *JAMA Psychiatry*.

eFigure 9. Odds ratios for top deciles of BIP PRS in AA participants.

For comparisons of the top PRS decile versus the remaining 90% (circles) and bottom 10% (triangles) displayed; error bars indicate the 95% CI for the estimated effect on selected phecodes. Displayed results are limited to phecodes with p-values < 10^{-5} (in either comparison) or replications (p<0.05) of significant EA findings.

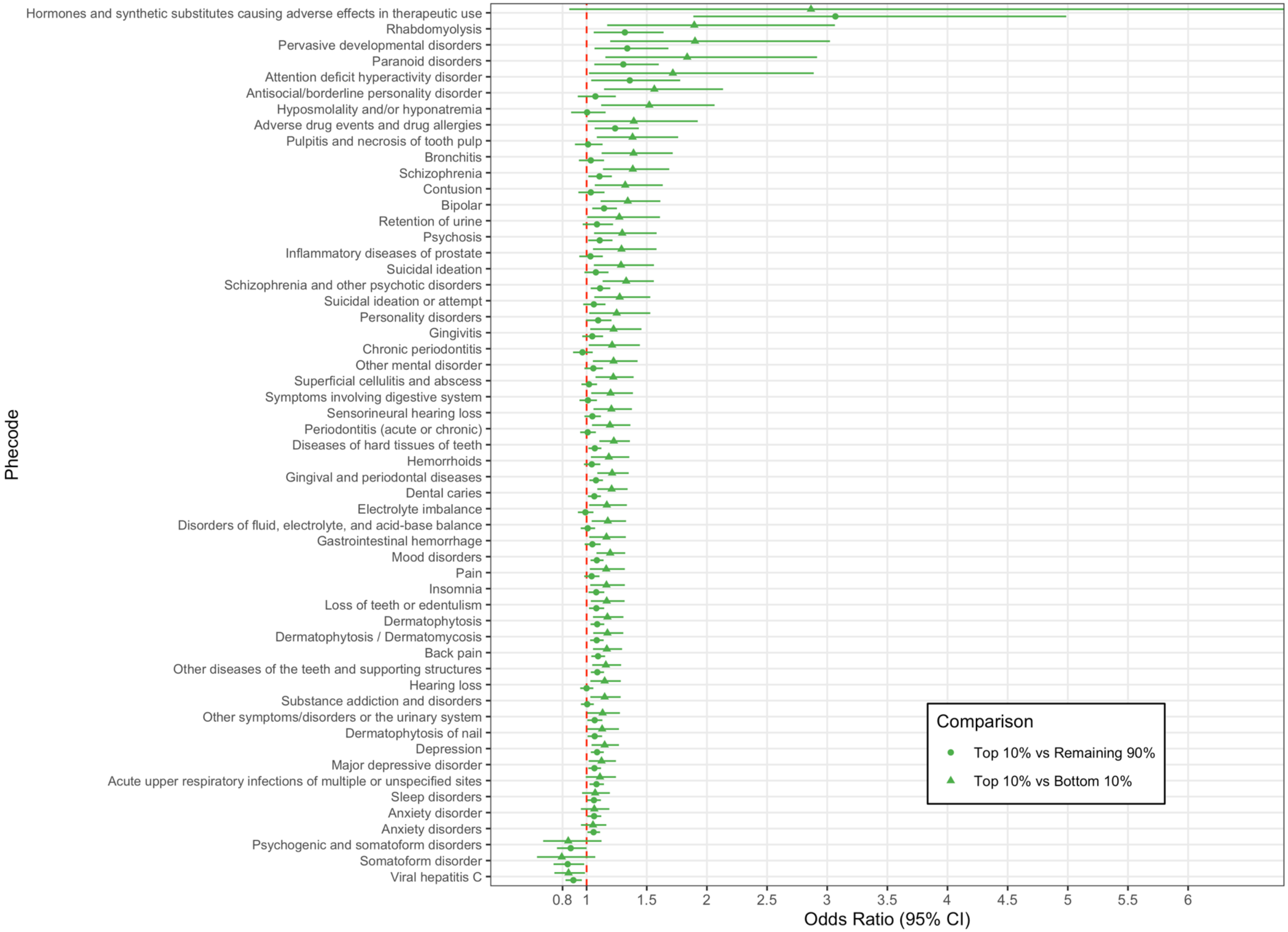

© 2022 Bigdeli TB et al. *JAMA Psychiatry*.

eFigure 10. Odds ratios for top deciles of DEP PRS in AA participants.
For comparisons of the top PRS decile versus the remaining 90% (circles) and bottom 10% (triangles) displayed; error bars indicate the 95% CI for the estimated effect on selected phecodes. Displayed results are limited to phecodes with p-values < 10^{-5} (in either comparison) or replications (p<0.05) of significant EA findings.

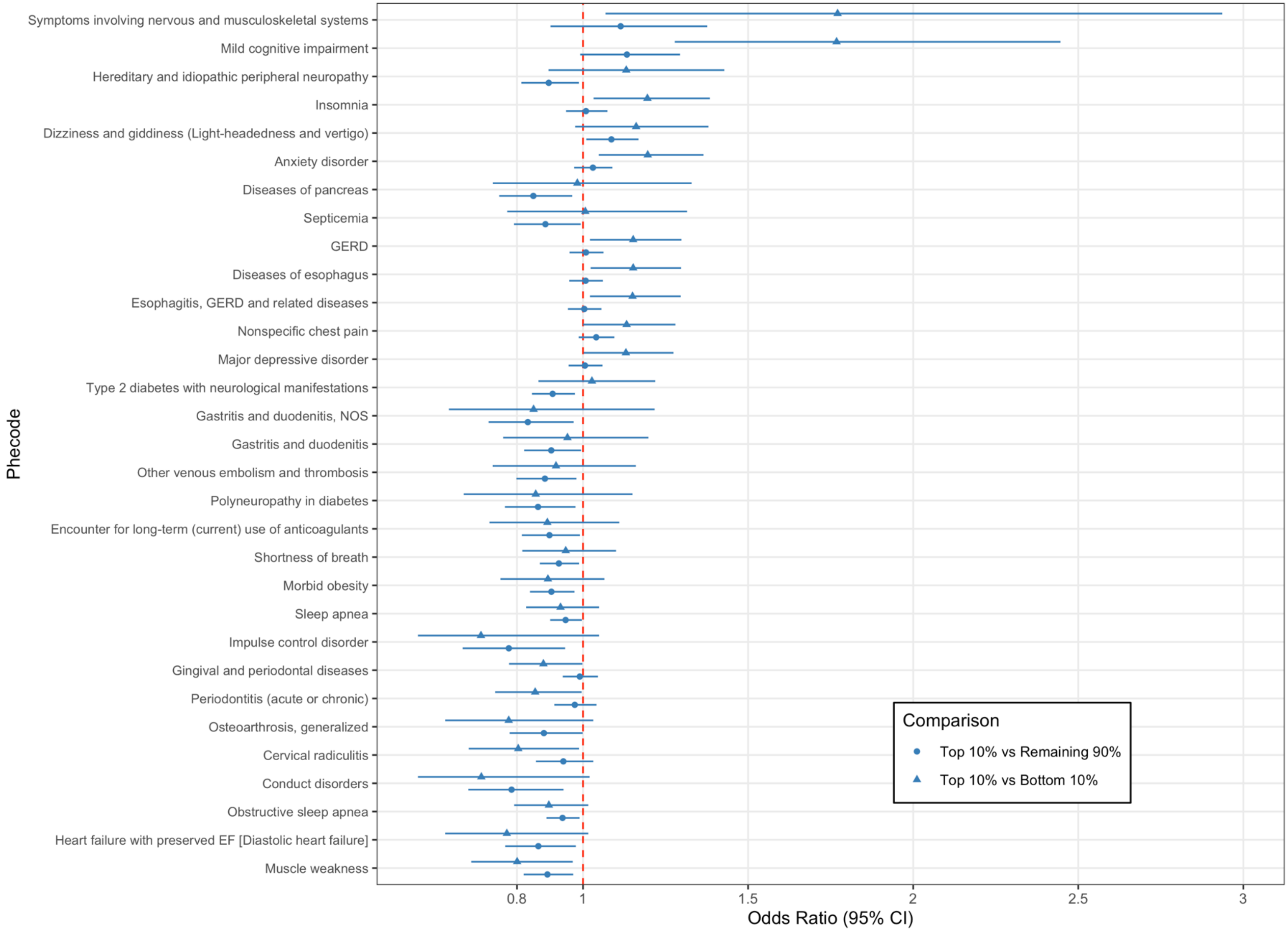

© 2022 Bigdeli TB et al. *JAMA Psychiatry*.
