# Supplementary material for: Penetrance and Pleiotropy of Polygenic Risk Scores for Schizophrenia, Bipolar Disorder, and Depression Among Adults in the US Veterans Affairs Health Care System
Source: JAMA Psychiatry. 2022 Sep 14;79(11):1092–101. doi: 10.1001/jamapsychiatry.2022.2742 (PMC9475441; doi:10.1001/jamapsychiatry.2022.2742)
Supplement: Supplement 2. — Nonauthor collaborators. The Cooperative Studies Program (CSP) #572 and Million Veteran Program (MVP) investigators [file jamapsychiatry-e222742-s002.pdf]

Supplemental Online Content: Nonauthor Collaborators
*First name, last name, and suffix (if applicable) are required and will appear in PubMed.

*Group Name(s): Cooperative Studies Program (CSP) #572 and Million Veteran Program (MVP)							
***First Name and Middle Initial(s)**	***Last Name**	***Suffix (eg, Jr, III)**	Academic Degrees	Institution	Location (city, state/province, country)	Role or Contribution, eg, chair, principal investigator	Group (if more than 1 Group listed in the byline) and/or Subgroup (eg, Steering Committee)
Mihaela	Aslan		PhD	Clinical Epidemiology Research Center (CERC), VA Connecticut Healthcare System, West Haven, CT, Yale University School of Medicine	New Haven, CT		CSP #572 Planning Committee; VA Clinical Epidemiology Research Center (CERC)
M	Antonelli						CSP #572 Planning Committee
M	de Asis						CSP #572 Planning Committee
MS	Bauer						CSP #572 Planning Committee
Mary	Brophy		MD MPH	Massachusetts Area Veterans Epidemiology, Research, and Information Center (MAVERIC), Jamaica Plain, MA, Boston University School of Medicine	Boston, MA		CSP #572 Planning Committee; CSP #572 Executive Committee; Million Veteran Program (MVP) Recruitment and Enrollment, Core Biorespository

Supplemental Online Content: Nonauthor Collaborators
*First name, last name, and suffix (if applicable) are required and will appear in PubMed.

***First Name and Middle Initial(s)**	***Last Name**	***Suffix (eg, Jr, III)**	Academic Degrees	Institution	Location (city, state/province, country)	Role or Contribution, eg, chair, principal investigator	Group (if more than 1 Group listed in the byline) and/or Subgroup (eg, Steering Committee)
John	Concato		MD	Yale University School of Medicine, New Haven, CT, Center for Drug Evaluation and Research, Food and Drug Administration	West Haven, CT		CSP #572 Planning Committee; CSP #572 Executive Committee; VA Clinical Epidemiology Rsearch Center (CERC) Director, Methodological Co-Principal Proponent; Million Veteran Program (MVP) Coordinating Center
F	Cunningham						CSP #572 Planning Committee
R	Freedman						CSP #572 Planning Committee
Michael	Gaziano		MD MPH	Massachusetts Area Veterans Epidemiology, Research, and Information Center (MAVERIC), Jamaica Plain, MA, Harvard Medical School	Boston, MA		CSP #572 Planning Committee; CSP #572 Executive Committee; Massachusetts Veterans Epidemiology Research and Information Center (MAVERIC) Director, Co-Principal Proponent; Million Veteran Program (MVP) Executive Committee (Co-Chair)

*First name, last name, and suffix (if applicable) are required and will appear in PubMed.

***First Name and Middle Initial(s)**	***Last Name**	***Suffix (eg, Jr, III)**	Academic Degrees	Institution	Location (city, state/province, country)	Role or Contribution, eg, chair, principal investigator	Group (if more than 1 Group listed in the byline) and/or Subgroup (eg, Steering Committee)
Theresa	Gleason		PhD	Office of Research and Development, Veterans Health Administration	Washington, D.C.		CSP #572 Planning Committee; VA Office of Research and Development, Director, Clinical Science Research and Development Service
Philip	Harvey		PhD	Bruce W. Carter Miami Veterans Affairs (VA) Medical Center, University of Miami Miller School of Medicine	Miami, FL	Study Co-Chair	CSP #572 Planning Committee; CSP #572 Executive Committee
Grant	Huang		MPH PhD	Office of Research and Development, Veterans Health Administration	Washington, D.C.		CSP #572 Planning Committee; VA Office of Research and Development, Director, Cooperative Studies Program; Million Veteran Program (MVP) Executive Committee
J	Kelsoe						CSP #572 Planning Committee
Thomas	Kosten		MD	Michael E. DeBakey VA Medical Center, Department of Psychiatry, Neuroscience, Pharmacology, and Immunology and Rheumatology, Baylor College of Medicine	Houston, TX		CSP #572 Planning Committee; CSP #572 Executive Committee
T	Lehner						CSP #572 Planning Committee

Supplemental Online Content: Nonauthor Collaborators
*First name, last name, and suffix (if applicable) are required and will appear in PubMed.

***First Name and Middle Initial(s)**	***Last Name**	***Suffix (eg, Jr, III)**	Academic Degrees	Institution	Location (city, state/province, country)	Role or Contribution, eg, chair, principal investigator	Group (if more than 1 Group listed in the byline) and/or Subgroup (eg, Steering Committee)
JB	Lohr						CSP #572 Planning Committee
SR	Marder						CSP #572 Planning Committee
P	Miller						CSP #572 Planning Committee
Timothy	O Leary		MD	Office of Research and Development, Veterans Health Administration	Washington, D.C.		CSP #572 Planning Committee; VA Office Research and Development, Chief Research and Development Officer Emeritus
T	Patterson						CSP #572 Planning Committee
P	Peduzzi						CSP #572 Planning Committee
Ronald	Przygodski		MD	Office of Research and Development, Veterans Health Administration	Washington, D.C.		CSP #572 Planning Committee; VA Office of Research and Development, Associate Director for Genomic Medicine and Acting Director of Biomedical Laboratory Research and Development Service
Larry	Siever			James J. Peters Veterans Affairs Medical Center, Icahn School of Medicine at Mount Sinai	Bronx, NY	Study Co-Chair	CSP #572 Planning Committee; CSP #572 Executive Committee

Supplemental Online Content: Nonauthor Collaborators
*First name, last name, and suffix (if applicable) are required and will appear in PubMed.

***First Name and Middle Initial(s)**	***Last Name**	***Suffix (eg, Jr, III)**	Academic Degrees	Institution	Location (city, state/province, country)	Role or Contribution, eg, chair, principal investigator	Group (if more than 1 Group listed in the byline) and/or Subgroup (eg, Steering Committee)
P	Sklar						CSP #572 Planning Committee; CSP #572 Executive Committee
S	Strakowski						CSP #572 Planning Committee
Hongyu	Zhao		PhD	Clinical Epidemiology Research Center (CERC), VA Connecticut Healthcare System, Yale University School of Medicine	New Haven, CT		CSP #572 Planning Committee; CSP #572 Executive Committee; MVP Science, Statistical Genetics
Ayman	Fanous		MD	VA New York Harbor Healthcare System, University of Arizona College of Medicine-Phoenix	Phoenix, AZ		CSP #572 Executive Committee; CSP #572 Site, Washington D.C.
W	Farwell						CSP #572 Executive Committee
A	Malhorta						CSP #572 Executive Committee
S	Mane						CSP #572 Executive Committee
P	Palacios						CSP #572 Executive Committee
Tim	Bigdeli		PhD	VA New York Harbor Healthcare System, Department of Psychiatry and Behavioral Sciences SUNY Downstate Health Sciences University	Brooklyn, NY		CSP #572 Executive Committee
M	Corsey			VA Healthcare System Bronx	Bronx, NY		CSP #572 Study Chairs' Office
L	Zaluda			VA Healthcare System Bronx	Bronx, NY		CSP #572 Study Chairs' Office

Supplemental Online Content: Nonauthor Collaborators
*First name, last name, and suffix (if applicable) are required and will appear in PubMed.

***First Name and Middle Initial(s)**	***Last Name**	***Suffix (eg, Jr, III)**	Academic Degrees	Institution	Location (city, state/province, country)	Role or Contribution, eg, chair, principal investigator	Group (if more than 1 Group listed in the byline) and/or Subgroup (eg, Steering Committee)
Juanita	Johnson		BSN	Bruce W. Carter Miami Veterans Affairs (VA) Medical Center	Miami, FL		CSP #572 Study Chairs' Office; CSP #572 Study Site, Miami
Melyssa	Sueiro		MS	Bruce W. Carter Miami Veterans Affairs (VA) Medical Center	Miami, FL		CSP #572 Study Chairs' Office
D	Cavaliere			Clinical Epidemiology Research Center (CERC), VA Connecticut Healthcare System	New Haven, CT		VA Clinical Epidemiology Research Center (CERC)
V	Jeanpaul			Clinical Epidemiology Research Center (CERC), VA Connecticut Healthcare System	New Haven, CT		VA Clinical Epidemiology Research Center (CERC)
Alysia	Maffucci		JD	Clinical Epidemiology Research Center (CERC), VA Connecticut Healthcare System	New Haven, CT		VA Clinical Epidemiology Research Center (CERC)
L	Mancini			Clinical Epidemiology Research Center (CERC), VA Connecticut Healthcare System	New Haven, CT		VA Clinical Epidemiology Research Center (CERC)
J	Deen						Massachusetts Veterans Epidemiology Research and Information Center (MAVERIC)
G	Muldoon						Massachusetts Veterans Epidemiology Research and Information Center (MAVERIC)

*First name, last name, and suffix (if applicable) are required and will appear in PubMed.

***First Name and Middle Initial(s)**	***Last Name**	***Suffix (eg, Jr, III)**	Academic Degrees	Institution	Location (city, state/province, country)	Role or Contribution, eg, chair, principal investigator	Group (if more than 1 Group listed in the byline) and/or Subgroup (eg, Steering Committee)
Stacey	Whitbourne		PhD		Boston, MA		Massachusetts Veterans Epidemiology Research and Information Center (MAVERIC); Million Veteran Program (MVP) Recruitment/Enrollment, Director/Deputy Director
J	Canive			Raymond G. Murphy VA Medical Center	Albuequerque, NM		CSP #572 Study Site, Albuquerque
L	Adamson			Raymond G. Murphy VA Medical Center	Albuequerque, NM		CSP #572 Study Site, Albuquerque
L	Calais			Raymond G. Murphy VA Medical Center	Albuequerque, NM		CSP #572 Study Site, Albuquerque
G	Fuldauer			Raymond G. Murphy VA Medical Center	Albuequerque, NM		CSP #572 Study Site, Albuquerque
R	Kushner			Raymond G. Murphy VA Medical Center	Albuequerque, NM		CSP #572 Study Site, Albuquerque
G	Toney			Raymond G. Murphy VA Medical Center	Albuequerque, NM		CSP #572 Study Site, Albuquerque
M	Lackey			Raymond G. Murphy VA Medical Center	Albuequerque, NM		CSP #572 Study Site, Albuquerque
A	Mank			Raymond G. Murphy VA Medical Center	Albuequerque, NM		CSP #572 Study Site, Albuquerque
N	Mahdavi			Raymond G. Murphy VA Medical Center	Albuequerque, NM		CSP #572 Study Site, Albuquerque
G	Villarreal			Raymond G. Murphy VA Medical Center	Albuequerque, NM		CSP #572 Study Site, Albuquerque
EC	Muly			Atlanta VA Medical Center	Atlanta, GA		CSP #572 Study Site, Atlanta

*First name, last name, and suffix (if applicable) are required and will appear in PubMed.

***First Name and Middle Initial(s)**	***Last Name**	***Suffix (eg, Jr, III)**	Academic Degrees	Institution	Location (city, state/province, country)	Role or Contribution, eg, chair, principal investigator	Group (if more than 1 Group listed in the byline) and/or Subgroup (eg, Steering Committee)
F	Amin			Atlanta VA Medical Center	Atlanta, GA		CSP #572 Study Site, Atlanta
M	Dent			Atlanta VA Medical Center	Atlanta, GA		CSP #572 Study Site, Atlanta
J	Wold			Atlanta VA Medical Center	Atlanta, GA		CSP #572 Study Site, Atla+A46:H59nta
B	Fischer			Baltimore VA Medical Center	Baltimore, MD		CSP #572 Study Site, Baltimore
A	Elliott			Baltimore VA Medical Center	Baltimore, MD		CSP #572 Study Site, Baltimore
C	Felix			Baltimore VA Medical Center	Baltimore, MD		CSP #572 Study Site, Baltimore
G	Gill			Baltimore VA Medical Center	Baltimore, MD		CSP #572 Study Site, Baltimore
PE	Parker			Birmingham VA Medical Center	Birmingham, AL		CSP #572 Site, Birmingham
C	Logan			Birmingham VA Medical Center	Birmingham, AL		CSP #572 Site, Birmingham
J	McAlpine			Birmingham VA Medical Center	Birmingham, AL		CSP #572 Site, Birmingham
LE	DeLisi			Brockton VA Medical Center	Brockton, MA		CSP #572 Site, Brockton
SG	Reece			Brockton VA Medical Center	Brockton, MA		CSP #572 Site, Brockton
MB	Hammer			Ralph H. Johnson VA Healthcare System	Charleston, SC		CSP #572 Site, Charleston
D	Agbor-Tabie			Ralph H. Johnson VA Healthcare System	Charleston, SC		CSP #572 Site, Charleston
W	Goodson			Ralph H. Johnson VA Healthcare System	Charleston, SC		CSP #572 Site, Charleston
M	Aslam			VA Cincinnati Healthcare System	Cincinnati, OH		CSP #572 Site, Cincinnati
M	Grainger			VA Cincinnati Healthcare System	Cincinnati, OH		CSP #572 Site, Cincinnati
Neil	Richtand			VA Cincinnati Healthcare System	Cincinnati, OH		CSP #572 Site, Cincinnati

*First name, last name, and suffix (if applicable) are required and will appear in PubMed.

***First Name and Middle Initial(s)**	***Last Name**	***Suffix (eg, Jr, III)**	Academic Degrees	Institution	Location (city, state/province, country)	Role or Contribution, eg, chair, principal investigator	Group (if more than 1 Group listed in the byline) and/or Subgroup (eg, Steering Committee)
Alexander	Rybalsky			VA Cincinnati Healthcare System	Cincinnati, OH		CSP #572 Site, Cincinnati
R	Al Jurdi			Michael E. DeBakey VA Medical Center	Houston, TX		CSP #572 Site, Houston
E	Boeckman			Michael E. DeBakey VA Medical Center	Houston, TX		CSP #572 Site, Houston
T	Natividad			Michael E. DeBakey VA Medical Center	Houston, TX		CSP #572 Site, Houston
D	Smith			Michael E. DeBakey VA Medical Center	Houston, TX		CSP #572 Site, Houston
M	Stewart			Michael E. DeBakey VA Medical Center	Houston, TX		CSP #572 Site, Houston
S	Torres			Michael E. DeBakey VA Medical Center	Houston, TX		CSP #572 Site, Houston
Z	Zhao			Michael E. DeBakey VA Medical Center	Houston, TX		CSP #572 Site, Houston
A	Mayeda			Richard L. Roudebush VA Medical Center	Indianapolois, IN		CSP #572 Site, Indianapolis
A	Green			Richard L. Roudebush VA Medical Center	Indianapolois, IN		CSP #572 Site, Indianapolis
J	Hofstetter			Richard L. Roudebush VA Medical Center	Indianapolois, IN		CSP #572 Site, Indianapolis
S	Ngombu			Richard L. Roudebush VA Medical Center	Indianapolois, IN		CSP #572 Site, Indianapolis
MK	Scott			Richard L. Roudebush VA Medical Center	Indianapolois, IN		CSP #572 Site, Indianapolis
A	Strasburger			Richard L. Roudebush VA Medical Center	Indianapolois, IN		CSP #572 Site, Indianapolis
J	Sumner			Richard L. Roudebush VA Medical Center	Indianapolois, IN		CSP #572 Site, Indianapolis
G	Paschall			John L. McClellan Memorial Veterans' Hospital	Little Rock, AR		CSP #572 Site, Little Rock

Supplemental Online Content: Nonauthor Collaborators
*First name, last name, and suffix (if applicable) are required and will appear in PubMed.

***First Name and Middle Initial(s)**	***Last Name**	***Suffix (eg, Jr, III)**	Academic Degrees	Institution	Location (city, state/province, country)	Role or Contribution, eg, chair, principal investigator	Group (if more than 1 Group listed in the byline) and/or Subgroup (eg, Steering Committee)
J	Mucciarelli			John L. McClellan Memorial Veterans' Hospital	Little Rock, AR		CSP #572 Site, Little Rock
R	Owen			John L. McClellan Memorial Veterans' Hospital	Little Rock, AR		CSP #572 Site, Little Rock
S	Theus			John L. McClellan Memorial Veterans' Hospital	Little Rock, AR		CSP #572 Site, Little Rock
D	Tompkins			John L. McClellan Memorial Veterans' Hospital	Little Rock, AR		CSP #572 Site, Little Rock
SG	Potkin			VA Long Beach Healthcare System	Long Beach, CA		CSP #572 Site, Long Beach
C	Reist			VA Long Beach Healthcare System	Long Beach, CA		CSP #572 Site, Long Beach
M	Novin			VA Long Beach Healthcare System	Long Beach, CA		CSP #572 Site, Long Beach
S	Khalaghizadeh			VA Long Beach Healthcare System	Long Beach, CA		CSP #572 Site, Long Beach
Richard	Douyon		MD	Bruce W. Carter Miami Veterans Affairs (VA) Medical Center	Miami, FL		CSP #572 Site, Miami
Nita	Kumar		MD	Bruce W. Carter Miami Veterans Affairs (VA) Medical Center	Miami, FL		CSP #572 Site, Miami
Becky	Martinez		MSN	Bruce W. Carter Miami Veterans Affairs (VA) Medical Center	Miami, FL		CSP #572 Site, Miami
SR	Sponheim			Minneapolis VA Healthcare System	Minneapolis, MN		CSP #572 Site, Minneapolois
TL	Bender			Minneapolis VA Healthcare System	Minneapolis, MN		CSP #572 Site, Minneapolois
HL	Lucas			Minneapolis VA Healthcare System	Minneapolis, MN		CSP #572 Site, Minneapolois
AM	Lyon			Minneapolis VA Healthcare System	Minneapolis, MN		CSP #572 Site, Minneapolois

Supplemental Online Content: Nonauthor Collaborators
*First name, last name, and suffix (if applicable) are required and will appear in PubMed.

*First Name and Middle Initial(s)	*Last Name	*Suffix (eg, Jr, III)	Academic Degrees	Institution	Location (city, state/province, country)	Role or Contribution, eg, chair, principal investigator	Group (if more than 1 Group listed in the byline) and/or Subgroup (eg, Steering Committee)
MP	Marggraf			Minneapolis VA Healthcare System	Minneapolis, MN		CSP #572 Site, Minneapolois
LH	Sorensen			Minneapolis VA Healthcare System	Minneapolis, MN		CSP #572 Site, Minneapolois
CR	Surerus			Minneapolis VA Healthcare System	Minneapolis, MN		CSP #572 Site, Minneapolois
C	Sison			Montrose VA Medical Center	Montrose, NY		CSP #572 Site, Montrose
J	Amato			Montrose VA Medical Center	Montrose, NY		CSP #572 Site, Montrose
DR	Johnson			Montrose VA Medical Center	Montrose, NY		CSP #572 Site, Montrose
N	Pagan-Howard			Montrose VA Medical Center	Montrose, NY		CSP #572 Site, Montrose
LA	Adler			VA New York Harbor Healthcare System	New York, NY		CSP #572 Site, New York Harbor
S	Alerpin			VA New York Harbor Healthcare System	New York, NY		CSP #572 Site, New York Harbor
T	Leon			VA New York Harbor Healthcare System	New York, NY		CSP #572 Site, New York Harbor
KM	Mattocks			Northampton VA Medical Center	Leeds, MA		CSP #572 Site, Northampton
N	Araeva			Northampton VA Medical Center	Leeds, MA		CSP #572 Site, Northampton
JC	Sullivan			Northampton VA Medical Center	Leeds, MA		CSP #572 Site, Northampton
T	Suppes			VA Palo Alto Health Care System	Palo Alto, CA		CSP #572 Site, Palo Alto
K	Bratcher			VA Palo Alto Health Care System	Palo Alto, CA		CSP #572 Site, Palo Alto
L	Drag			VA Palo Alto Health Care System	Palo Alto, CA		CSP #572 Site, Palo Alto
EG	Fischer			VA Palo Alto Health Care System	Palo Alto, CA		CSP #572 Site, Palo Alto
L	Fujitani			VA Palo Alto Health Care System	Palo Alto, CA		CSP #572 Site, Palo Alto
S	Gill			VA Palo Alto Health Care System	Palo Alto, CA		CSP #572 Site, Palo Alto
D	Grimm			VA Palo Alto Health Care System	Palo Alto, CA		CSP #572 Site, Palo Alto
J	Hoblyn			VA Palo Alto Health Care System	Palo Alto, CA		CSP #572 Site, Palo Alto
T	Nguyen			VA Palo Alto Health Care System	Palo Alto, CA		CSP #572 Site, Palo Alto

Supplemental Online Content: Nonauthor Collaborators
*First name, last name, and suffix (if applicable) are required and will appear in PubMed.

***First Name and Middle Initial(s)**	***Last Name**	***Suffix (eg, Jr, III)**	Academic Degrees	Institution	Location (city, state/province, country)	Role or Contribution, eg, chair, principal investigator	Group (if more than 1 Group listed in the byline) and/or Subgroup (eg, Steering Committee)
E	Nikolaev			VA Palo Alto Health Care System	Palo Alto, CA		CSP #572 Site, Palo Alto
L	Shere			VA Palo Alto Health Care System	Palo Alto, CA		CSP #572 Site, Palo Alto
R	Relova			VA Palo Alto Health Care System	Palo Alto, CA		CSP #572 Site, Palo Alto
A	Vicencio			VA Palo Alto Health Care System	Palo Alto, CA		CSP #572 Site, Palo Alto
M	Yip			VA Palo Alto Health Care System	Palo Alto, CA		CSP #572 Site, Palo Alto
I	Hurford			Corporal Michael J. Crescenz VA Medical Center	Philadelphia, PA		CSP #572 Site, Philadelphia
S	Acheampong			Corporal Michael J. Crescenz VA Medical Center	Philadelphia, PA		CSP #572 Site, Philadelphia
G	Carfagno			Corporal Michael J. Crescenz VA Medical Center	Philadelphia, PA		CSP #572 Site, Philadelphia
GL	Haas			VA Pittsburgh Healthcare System	Pittsburgh, PA		CSP #572 Site, Pittsburgh
C	Appelt			VA Pittsburgh Healthcare System	Pittsburgh, PA		CSP #572 Site, Pittsburgh
E	Brown			VA Pittsburgh Healthcare System	Pittsburgh, PA		CSP #572 Site, Pittsburgh
B	Chakraborty			VA Pittsburgh Healthcare System	Pittsburgh, PA		CSP #572 Site, Pittsburgh
E	Kelly			VA Pittsburgh Healthcare System	Pittsburgh, PA		CSP #572 Site, Pittsburgh
G	Klima			VA Pittsburgh Healthcare System	Pittsburgh, PA		CSP #572 Site, Pittsburgh
S	Steinhauer			VA Pittsburgh Healthcare System	Pittsburgh, PA		CSP #572 Site, Pittsburgh
RA	Hurley			W.G. (Bill) Hefner VA Medical Center	Salisbury, NC		CSP #572 Site, Salisbury
R	Belle			W.G. (Bill) Hefner VA Medical Center	Salisbury, NC		CSP #572 Site, Salisbury
D	Eknoyan			W.G. (Bill) Hefner VA Medical Center	Salisbury, NC		CSP #572 Site, Salisbury
K	Johnson			W.G. (Bill) Hefner VA Medical Center	Salisbury, NC		CSP #572 Site, Salisbury
J	Lamotte			W.G. (Bill) Hefner VA Medical Center	Salisbury, NC		CSP #572 Site, Salisbury
E	Granholm			San Diego VA Medical Center	San Diego, CA		CSP #572 Site, San Diego
K	Bradshaw			San Diego VA Medical Center	San Diego, CA		CSP #572 Site, San Diego
J	Holden			San Diego VA Medical Center	San Diego, CA		CSP #572 Site, San Diego

Supplemental Online Content: Nonauthor Collaborators
*First name, last name, and suffix (if applicable) are required and will appear in PubMed.

***First Name and Middle Initial(s)**	***Last Name**	***Suffix (eg, Jr, III)**	Academic Degrees	Institution	Location (city, state/province, country)	Role or Contribution, eg, chair, principal investigator	Group (if more than 1 Group listed in the byline) and/or Subgroup (eg, Steering Committee)
RH	Jones			San Diego VA Medical Center	San Diego, CA		CSP #572 Site, San Diego
T	Le			San Diego VA Medical Center	San Diego, CA		CSP #572 Site, San Diego
IG	Molina			San Diego VA Medical Center	San Diego, CA		CSP #572 Site, San Diego
M	Peyton			San Diego VA Medical Center	San Diego, CA		CSP #572 Site, San Diego
I	Ruiz			San Diego VA Medical Center	San Diego, CA		CSP #572 Site, San Diego
L	Sally			San Diego VA Medical Center	San Diego, CA		CSP #572 Site, San Diego
A	Tapp			VA Medical Center- Tacoma	Tacoma, WA		CSP #572 Site, Tacoma
S	Devroy			VA Medical Center- Tacoma	Tacoma, WA		CSP #572 Site, Tacoma
V	Jain			VA Medical Center- Tacoma	Tacoma, WA		CSP #572 Site, Tacoma
N	Kilzieh			VA Medical Center- Tacoma	Tacoma, WA		CSP #572 Site, Tacoma
L	Maus			VA Medical Center- Tacoma	Tacoma, WA		CSP #572 Site, Tacoma
K	Miller			VA Medical Center- Tacoma	Tacoma, WA		CSP #572 Site, Tacoma
H	Pope			VA Medical Center- Tacoma	Tacoma, WA		CSP #572 Site, Tacoma
A	Wood			VA Medical Center- Tacoma	Tacoma, WA		CSP #572 Site, Tacoma
E	Meyer			Olin E. Teague Veterans' Medical Center (Temple)	Temple, TX		CSP #572 Site, Temple
P	Givens			Olin E. Teague Veterans' Medical Center (Temple)	Temple, TX		CSP #572 Site, Temple
PB	Hicks			Olin E. Teague Veterans' Medical Center (Temple)	Temple, TX		CSP #572 Site, Temple
S	Justice			Olin E. Teague Veterans' Medical Center (Temple)	Temple, TX		CSP #572 Site, Temple
K	McNair			Olin E. Teague Veterans' Medical Center (Temple)	Temple, TX		CSP #572 Site, Temple
JL	Pena			Olin E. Teague Veterans' Medical Center (Temple)	Temple, TX		CSP #572 Site, Temple
DF	Tharp			Olin E. Teague Veterans' Medical Center (Temple)	Temple, TX		CSP #572 Site, Temple
L	Davis			Tuscaloosa VA Medical Center	Tuscaloosa, AL		CSP #572 Site, Tuscaloosa

*First name, last name, and suffix (if applicable) are required and will appear in PubMed.

***First Name and Middle Initial(s)**	***Last Name**	***Suffix (eg, Jr, III)**	Academic Degrees	Institution	Location (city, state/province, country)	Role or Contribution, eg, chair, principal investigator	Group (if more than 1 Group listed in the byline) and/or Subgroup (eg, Steering Committee)
M	Ban			Tuscaloosa VA Medical Center	Tuscaloosa, AL		CSP #572 Site, Tuscaloosa
L	Cheatum			Tuscaloosa VA Medical Center	Tuscaloosa, AL		CSP #572 Site, Tuscaloosa
P	Darr			Tuscaloosa VA Medical Center	Tuscaloosa, AL		CSP #572 Site, Tuscaloosa
W	Grayson			Tuscaloosa VA Medical Center	Tuscaloosa, AL		CSP #572 Site, Tuscaloosa
J	Munford			Tuscaloosa VA Medical Center	Tuscaloosa, AL		CSP #572 Site, Tuscaloosa
D	Smith			Tuscaloosa VA Medical Center	Tuscaloosa, AL		CSP #572 Site, Tuscaloosa
B	Whitfield			Tuscaloosa VA Medical Center	Tuscaloosa, AL		CSP #572 Site, Tuscaloosa
E	Wilson			Tuscaloosa VA Medical Center	Tuscaloosa, AL		CSP #572 Site, Tuscaloosa
SE	Melnikoff			VA Washington DC Healthcare System	Washington, D.C.		CSP #572 Site, Washington, D.C.
BL	Schwartz			VA Washington DC Healthcare System	Washington, D.C.		CSP #572 Site, Washington, D.C.
MA	Tureson			VA Washington DC Healthcare System	Washington, D.C.		CSP #572 Site, Washington, D.C.
D	D Souza			West Haven VA Medical Center	West Haven, CT		CSP #572 Site, West Haven
K	Forselius			West Haven VA Medical Center	West Haven, CT		CSP #572 Site, West Haven
M	Ranganathan			West Haven VA Medical Center	West Haven, CT		CSP #572 Site, West Haven
L	Rispoli			West Haven VA Medical Center	West Haven, CT		CSP #572 Site, West Haven

Supplemental Online Content: Nonauthor Collaborators
*First name, last name, and suffix (if applicable) are required and will appear in PubMed.

***First Name and Middle Initial(s)**	***Last Name**	***Suffix (eg, Jr, III)**	Academic Degrees	Institution	Location (city, state/province, country)	Role or Contribution, eg, chair, principal investigator	Group (if more than 1 Group listed in the byline) and/or Subgroup (eg, Steering Committee)
M	Sather			New Mexico VA Healthcare System	Albuequerque, NM		CSP #572 CSP Coordinating Center (for monitoring), Director
C	Colling			New Mexico VA Healthcare System	Albuequerque, NM		CSP #572 CSP Coordinating Center (for monitoring)
C	Haakenson			New Mexico VA Healthcare System	Albuequerque, NM		CSP #572 CSP Coordinating Center (for monitoring)
D	Kruegar			New Mexico VA Healthcare System	Albuequerque, NM		CSP #572 CSP Coordinating Center (for monitoring)
Sumitra	Muralidhar		PhD	Office of Research and Development, Veterans Health Administration	Washington, D.C.		VA Office Research and Development, Senior Scientific Program Mnaager Genomic Medicine Program, Biomedical and Clinical R&D Services; Million Veteran Program (MVP) Executive Committee; MVP Program Office
Rachel	Ramoni		DMD ScD	Office of Research and Development, Veterans Health Administration	Washington, D.C.		Million Veteran Program (MVP) Executive Committee (Co-Chair)
Jim	Breeling		MD				Million Veteran Program (MVP) Executive Committee

Supplemental Online Content: Nonauthor Collaborators
*First name, last name, and suffix (if applicable) are required and will appear in PubMed.

***First Name and Middle Initial(s)**	***Last Name**	***Suffix (eg, Jr, III)**	Academic Degrees	Institution	Location (city, state/province, country)	Role or Contribution, eg, chair, principal investigator	Group (if more than 1 Group listed in the byline) and/or Subgroup (eg, Steering Committee)
Kyong-Mi	Chang		MD	Corporal Michael J. Crescenz VA Medical Center	Philadelphia, PA		Million Veteran Program (MVP) Executive Committee
Christopher	O Donnell		MD MPH	VA Boston Healthcare System	Boston, MA		Million Veteran Program (MVP) Executive Committee; MVP Science, Genomics
Philip	Tsao		PhD	VA Palo Alto Health Care System	Palo Alto, CA		Million Veteran Program (MVP) Executive Committee; MVP Coordinating Center, Genomics Coordinating Center; MVP Science, Genomics
Jennifer	Moser		PhD	Office of Research and Development, Veterans Health Administration	Washington, D.C.		Million Veteran Program (MVP) Office
Jessica	Brewer		MPH	VA Boston Healthcare System	Boston, MA		Million Veteran Program (MVP) Recruitment and Enrollment
Stuart	Warren		JD PharmD	New Mexico VA Healthcare System	Albuequerque, NM		Million Veteran Program (MVP) Recruitment and Enrollment, MVP Coordinating Center, Cooperative Studies Program Clinical Research Pharmacy Coordinating Center

Supplemental Online Content: Nonauthor Collaborators
*First name, last name, and suffix (if applicable) are required and will appear in PubMed.

***First Name and Middle Initial(s)**	***Last Name**	***Suffix (eg, Jr, III)**	Academic Degrees	Institution	Location (city, state/province, country)	Role or Contribution, eg, chair, principal investigator	Group (if more than 1 Group listed in the byline) and/or Subgroup (eg, Steering Committee)
Dean	Argyres		MS	New Mexico VA Healthcare System	Albuequerque, NM		Million Veteran Program (MVP) Recruitment and Enrollment, MVP Coordinating Center, Cooperative Studies Program Clinical Research Pharmacy Coordinating Center
Brady	Stevens		MS				Million Veteran Program (MVP) Recruitment and Enrollment, MVP Information Center-Canandaiggua
Donald	Humphries		PhD	VA Boston Healthcare System	Boston, MA		Million Veteran Program (MVP) Recruitment and Enrollment, Core Biorespository
Nhan	Do		MD	VA Boston Healthcare System	Boston, MA		Million Veteran Program (MVP) Recruitment and Enrollment, Informatics
Shahpoor	Shayan			VA Boston Healthcare System	Boston, MA		Million Veteran Program (MVP) Recruitment and Enrollment, Informatics

Supplemental Online Content: Nonauthor Collaborators
*First name, last name, and suffix (if applicable) are required and will appear in PubMed.

***First Name and Middle Initial(s)**	***Last Name**	***Suffix (eg, Jr, III)**	Academic Degrees	Institution	Location (city, state/province, country)	Role or Contribution, eg, chair, principal investigator	Group (if more than 1 Group listed in the byline) and/or Subgroup (eg, Steering Committee)
Xuan-Mai	Nguyen		PhD	VA Boston Healthcare System	Boston, MA		Million Veteran Program (MVP) Recruitment and Enrollment, Data Operations/Analytics
Saiju	Pyarajan		PhD	VA Boston Healthcare System	Boston, MA		Million Veteran Program (MVP) Science, Genomics; MVP Science, Data and Computational Sciences
Kelly	Cho		MPH PhD	VA Boston Healthcare System	Boston, MA		Million Veteran Program (MVP) Science, Phenomics
Elizabeth	Hauser		PhD	Duke University School of Medicine	Durham, NC		Million Veteran Program (MVP) Science, Statistical Genetics
Yan	Sun		PhD				Million Veteran Program (MVP) Science, Statistical Genetics
Peter	Wilson		MD	Atlanta VA Medical Center	Atlanta, GA	Local Site Investigator	Million Veteran Program (MVP)
Rachel	McArdle		PhD	Bay Pines VA Healthcare System	Bay Pines, FL	Local Site Investigator	Million Veteran Program (MVP)
Louis	Dellitalia		MD	Birmingham VA Medical Center	Birmingham, AL	Local Site Investigator	Million Veteran Program (MVP)
John	Harley		MD PhD	Cincinnati VA Healthcare System	Cincinnati, OH	Local Site Investigator	Million Veteran Program (MVP)
Jeffrey	Whittle		MD MPH	Clement J. Zablocki VA Medical Center	Milwaukee, WI	Local Site Investigator	Million Veteran Program (MVP)
